# Comparison of Published Estimates of the National Prevalence of Iron, Vitamin A, and Zinc Deficiency and Sources of Inconsistencies

**DOI:** 10.1016/j.advnut.2023.08.011

**Published:** 2023-08-25

**Authors:** Sonja Y. Hess, K Ryan Wessells, Demewoz Haile, Lisa M. Rogers, Xiuping Tan, Jonathan G. Barros, Megan W. Bourassa, Jonathan Gorstein, Kenneth H. Brown

**Affiliations:** 1 Institute for Global Nutrition and Department of Nutrition, University of California Davis, Davis, CA, United States; 2 Micronutrient Forum, Washington, DC, United States; 3 Institute for Health Metrics and Evaluation, University of Washington, Seattle, WA, United States; 4 World Health Organization, Geneva, Switzerland; 5 Bill & Melinda Gates Foundation, Seattle, WA, United States

**Keywords:** iron, vitamin A, zinc, micronutrient deficiency, prevalence

## Abstract

Micronutrient deficiencies result in a broad range of adverse health and functional consequences, but the true prevalence of specific deficiencies remains uncertain because limited information is available from nationally representative surveys using recommended biomarkers. The present review compares various reported national deficiency prevalence estimates for nutrients and years where the estimates overlap for individual countries that conducted nationally representative surveys and explores possible reasons for any discrepancies discovered. Nationally representative micronutrient status surveys that were conducted since 2000 among preschool-aged children or women of reproductive age and included assessment of iron, vitamin A, or zinc status based on recognized biomarkers were considered eligible for inclusion, along with any modeled deficiency prevalence estimates for these same countries and years. There was considerable variation across different published prevalence estimates, with larger inconsistencies when the prevalence estimate was based on proxies, such as hemoglobin for iron deficiency and dietary zinc availability for zinc deficiency. Numerous additional methodological issues affected the prevalence estimates, such as which biomarker and what cutoff was used to define deficiency, whether the biomarker was adjusted for inflammation, and what adjustment method was used. For some country-years, the various approaches resulted in fairly consistent prevalence estimates. For other country-years, however, the results differed markedly and changed the conclusions regarding the existence and severity of the micronutrient deficiency as a public health concern. In conclusion, to determine micronutrient status, we consider the assessment of one of the recommended biomarkers in a population representative survey as the best available information. If indicated, results should be adjusted for inflammation and generally acceptable cutoffs should be applied to facilitate comparisons, although individual countries may also apply nationally defined cutoffs to determine when and where to intervene. Global consensus is needed on best practices for presenting survey results and defining the prevalence of deficiency.

**Statement of Significance**

The present review compares and visualizes publicly available estimates of the national prevalence of deficiencies in iron, vitamin A, and zinc among preschool-aged children and women of reproductive age. Inconsistencies between estimates are particularly striking when proxy indicators were used to fill gaps in available biomarker information.

## Introduction

Vitamin and mineral deficiencies result in a broad range of adverse health and functional consequences, and these micronutrient deficiencies continue to be a public health concern globally, especially in low- and middle-income countries [1,2]. However, the true prevalence of specific micronutrient deficiencies remains uncertain because of the lack of reliable information obtained from nationally representative surveys using widely recognized biomarkers of micronutrient status [3]. Vitamin and mineral status and the presence of deficiency are best defined in relation to *1*) total body stores of the nutrient, *2*) concentrations of the nutrient in specific tissues that represent major storage sites, or *3*) total or metabolically active pool sizes, as measured using tracer dilution methodology. For example, iron deficiency can be defined as the absence of stainable iron in bone marrow, which is the level of depletion at which erythropoiesis begins to be impaired [4]; vitamin A deficiency can be defined as total body stores or hepatic concentration of vitamin A below the threshold at which the likelihood of xerophthalmia increases [5]; and zinc deficiency can be defined as depletion of the rapidly exchangeable zinc pool size [6]. However, these assessment methods are overly invasive and costly for routine clinical application or population status assessment. Thus, biomarkers of micronutrient status that are associated with the aforementioned reference standards and are measurable in peripheral blood are used instead. For example, serum ferritin concentration is associated with iron reserves in bone marrow [4,7], soluble transferrin receptor (sTfR) is an indicator of the severity of iron insufficiency only when iron stores are depleted and no other causes of abnormal erythropoiesis are known [4], serum retinol concentration is associated with hepatic vitamin A concentration when the liver is depleted of vitamin A [8], and serum zinc concentration is associated with the rapidly exchangeable zinc pool size and clinical signs of zinc deficiency [6,9]. Despite some limitations, these blood-derived biomarkers have been used to assess both individual and population micronutrient status.

Several international data archives and research groups report estimates of national or global prevalence of selected micronutrient deficiencies considered to be of possible public health importance. These estimates are based on biomarkers of micronutrient status or possible proxies of biomarker information. The Micronutrients Database in the Vitamin and Mineral Nutrition Information System (VMNIS) collated by the WHO posts prevalence of deficiency of various vitamins and minerals in populations, primarily obtained from nationally representative nutrition surveys [10]. The Biomarkers Reflecting Inflammation and Nutritional Determinants of Anemia (BRINDA) project has also assembled nationally representative data sets with individual-level data from a number of countries and has reanalyzed the data to adjust for the effects of inflammation on the blood-based biomarkers [11,12]. Through its Global Burden of Disease (GBD) study, the GBD Collaboration has published regional and country-specific estimates of selected micronutrient deficiencies for each year since 1990. Because nationally representative biomarker data is limited, modelers such as the Institute for Health Metrics and Evaluation (IHME) sometimes rely on proxy indicators when estimating the prevalence of iron and zinc deficiencies for the GBD Study [13]. Examples include the use of hemoglobin as a proxy for iron deficiency anemia [14,15] and dietary availability of zinc in the national food supply based on supply utilization accounts (SUAs) prepared by the FAO [15] as a proxy for zinc deficiency. In addition, because of the limited data available, the GBD Collaboration uses complex statistical modeling to estimate country-level prevalence of deficiency, applying numerous assumptions [16].

Because of the different methods used by the foregoing data archives, there are likely inconsistencies in the estimated prevalence of deficiencies. The present review paper focuses on deficiencies of iron, vitamin A, and zinc because of their relative public health significance, the greater availability of information on these nutrients, and the fact that several methodological considerations can influence the respective deficiency prevalence estimates. Factors that may affect these estimates are: *1*) the choice of biomarker for a particular nutrient, *2*) the cutoff used to define deficiency, *3*) whether or not and how adjustments were made for the effects of inflammation, and *4*) the use of proxy indicators in situations where limited information is available on the biomarkers of interest. The objectives of this paper are *1*) to compare the prevalence estimates reported by various institutions for selected micronutrients and years where the estimates overlap for individual countries that conducted nationally representative surveys, and *2*) to explore possible reasons for any discrepancies discovered.

## Methods

Nationally representative micronutrient surveys that were conducted since 2000 and included assessment of iron, vitamin A, or zinc status based on biomarkers were considered eligible for inclusion in the present review if they were published either in the VMNIS Micronutrients Database [10] and/or by the BRINDA project [[17], [18], [19], [20], [21]]. Eligibility criteria for surveys to be included in VMNIS were: explicit sampling frame of defined population, sample representative at national, regional, and first administrative level (i.e., state, canton, province), population-, household- or facility-based sample, cross-sectional sample, and standard validated data collection techniques and laboratory methods of recommended biomarkers [22,23]. Inclusion criteria for BRINDA were similar except that the BRINDA investigators considered only household-based surveys and required that C-reactive protein (CRP) and/or α-1-acid glycoprotein (AGP) was measured [24]. For the present review, population groups of interest were children 6 to 59 mo of age (preschool-aged children, PSC) and nonpregnant women 15 to 49 y of age (women of reproductive age, WRA). If survey results were reported for various age ranges, the results for the population group closest to the target age range was recorded. First, relevant data were downloaded from VMNIS into Excel files. This process was repeated twice to confirm eligibility of selected surveys. The downloaded records are available online [25]. Methodological details of all selected surveys were summarized by biomarker and population group based on the information available from VMNIS (Supplemental Tables 1–6). The VMINS reports the deficiency prevalence results as presented in the original country reports and provides information in comment boxes on whether the prevalence was adjusted for inflammation and how the adjustments were done. For the present study, the VMNIS’ descriptions of inflammation adjustments were summarized independently by 2 reviewers (SYH, KRW) based on notes in the VMNIS database, and in case of discrepancies between the 2 reviewers, summaries were compared, and a consensus summary was included in the online Supplemental Tables 1–6. The VMNIS includes surveys that had applied various methods to adjust for the presence of inflammation, such as *1*) increasing or decreasing (depending on which marker) the biomarker cutoff in populations with a high burden of infection, *2*) excluding results of individuals with inflammation, and *3*) statistical approaches using CRP and AGP, such as the methods suggested by Thurnham et al. and BRINDA [[7], [11], [26]]. Briefly, the method by Thurnham et al., which we henceforth refer to as “categorical,” relies on internal correction factors and quantifies 3 different phases of inflammation (acute phase with only CRP elevated; early convalescence with both CRP and AGP elevated; and late convalescence with just AGP elevated) compared with a reference group with neither CRP nor AGP elevated [[27], [26], [28]]. The BRINDA approach relies on linear regression to adjust biomarkers for inflammation using the maximum values of the lowest decile category for CRP and AGP as the reference group [11,12].

The prevalence of deficiencies in iron, vitamin A, and zinc was summarized as reported in the WHO’s VMNIS. The estimated prevalence of deficiency before and after correction for inflammation as reported by BRINDA was retrieved for surveys included in the BRINDA project [[17], [18], [19], [20], [21]]. For all thus identified surveys with nationally representative biomarker data, we searched for other published estimates of micronutrient deficiency for the same country and year in the GBD database [29]. Eligible data included modeled estimates based on biomarker results, proxy data and/or estimates of dietary inadequacy. Specifically, the prevalence estimates for dietary iron deficiency as a cause of anemia, as modeled based on hemoglobin concentration in the GBD 2019 Study [14,15], were downloaded from the GBD result viz tool [29]. The GBD 2019 estimates of prevalence of vitamin A and zinc deficiency were compiled by IHME from the IHME database and provided for inclusion in the present paper. The detailed methods of GBD 2019 Study were reported elsewhere [14,15]. Briefly, the prevalence of vitamin A deficiency was estimated based on serum retinol concentration from VMNIS, and prevalence estimates for each year and location were modeled using spatiotemporal Gaussian process regression [15]. The prevalence of zinc deficiency was estimated based on inadequate dietary zinc availability and “cross-walked” with 24-h recall dietary surveys (i.e., the national dietary zinc availability was translated into mean 24-h zinc intake using GBD’s Bayesian meta-regression tool).

We summarized the abovementioned deficiency prevalence estimates in overview tables separately for children and women. To visualize these prevalence estimates side-by-side, we generated heatmaps categorizing the prevalence as <5%, 5% to 19.9%, 20% to 39.9%, and ≥40%, categories that have been suggested for defining the severity of the public health concern for anemia and iron deficiency [7,30]. We further created paired data comparisons for those surveys in which more than one result was available for the same country-year, such as the prevalence based on 2 different biomarkers, unadjusted and adjusted for inflammation, adjusted using different approaches, and estimated based on proxies for the GBD 2019 Study.

In an effort to compare biomarker-estimated prevalence of deficiency with the prevalence of dietary inadequacy, we also searched for estimates of inadequate dietary iron, vitamin A, and zinc intakes based on 24-h recalls assessed in nationally representative surveys for the same country-year as the biomarker data. We reached out to various experts in the field of dietary surveys to identify relevant data. We identified published results for Cameroon-2009, Kenya-2011, and Mexico-2012 and summarized characteristics of study populations, sample sizes, and results.

Estimates for micronutrient intakes based on food availability using food balance sheets and SUAs were also considered for the present study. However, dietary zinc availability using food balance sheets estimated the dietary zinc inadequacy for the whole population at the national level and not by the population subgroups of interest (i.e., PSC and WRA) [31]. Thus, we decided against including these in the overview tables.

## Results

Of the identified nationally representative surveys among young children, 70 included one or more biomarkers of iron status (ferritin, *n* = 64; sTfR, *n* = 27), 82 included one or more indicators of vitamin A status (retinol, *n* = 57; retinol binding protein [RBP], *n* = 27), and 28 measured plasma zinc concentration. Among women, nationally representative surveys included ferritin in 74, sTfR in 26, retinol in 35, RBP in 21, and plasma zinc in 22. Altogether, 59 countries collected information on iron status, 59 on vitamin A status, and 26 on zinc status.

### Prevalence of iron deficiency

**FIGURE 1:**
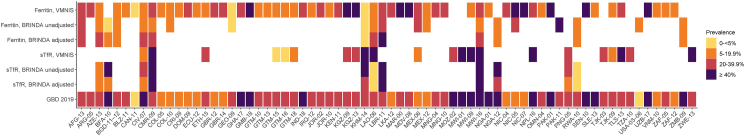
Prevalence of iron deficiency among young children in countries with nationally representative survey results as reported in VMNIS and by BRINDA and prevalence of inadequate dietary iron intake estimated for the GBD 2019 Study.^1^ BRINDA, Biomarkers Reflecting Inflammation and Nutritional Determinants of Anemia; GBD, Global Burden of Disease Study; sTfR, soluble transferrin receptor; VMNIS, Vitamin and Mineral Nutrition Information System. ^1^ Country survey codes, detailed results, and methods for adjustment of inflammation are reported in Supplemental Table S7.

**FIGURE 2:**
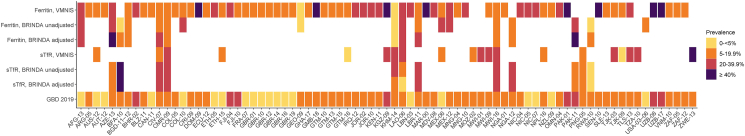
Prevalence of iron deficiency among women of reproductive age in countries with nationally representative survey results as reported in VMNIS and by BRINDA and prevalence of inadequate dietary iron intake estimated for the GBD 2019 Study.^1^ BRINDA, Biomarkers Reflecting Inflammation and Nutritional Determinants of Anemia; GBD, Global Burden of Disease Study; sTfR, soluble transferrin receptor; VMNIS, Vitamin and Mineral Nutrition Information System. ^1^ Country survey codes, detailed results, and methods for adjustment of inflammation are reported in Supplemental Table S8.

**FIGURE 3:**
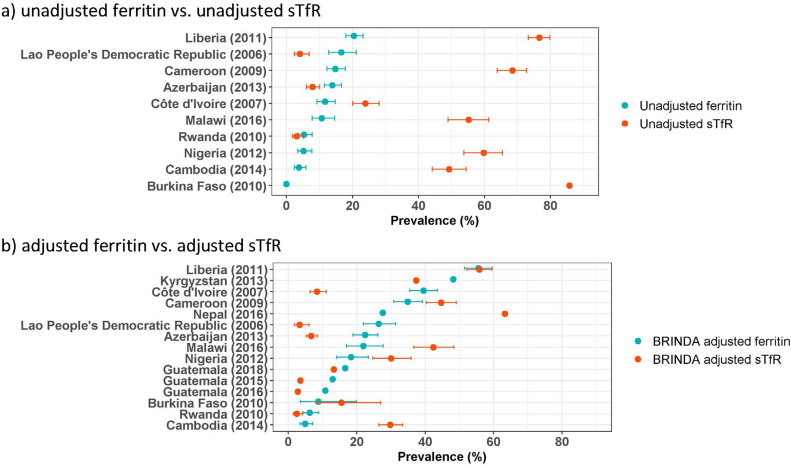
Comparison of iron deficiency prevalence among preschool-aged children from nationally representative surveys, estimated based on serum concentrations of ferritin or soluble transferrin receptor unadjusted or adjusted for inflammation.^1^ BRINDA, Biomarkers Reflecting Inflammation and Nutritional Determinants of Anemia; sTfR, soluble transferrin receptor. ^1^ Results shown as prevalence (%) with 95% confidence interval, where available. Detailed results and methods for adjustment of inflammation are reported in Supplemental Table S7.

The prevalence estimates for iron deficiency among PSC and WRA derived using different methods indicated similar public health severity for some countries but differed for others (FIGURE 1, FIGURE 2). To assess iron status, ferritin and sTfR were frequently measured in the same surveys. With few exceptions, the prevalence of low unadjusted ferritin among PSC and WRA was substantially lower than the prevalence of elevated unadjusted sTfR in the same survey (Figure 3A; Supplemental Tables 7, 8). In contrast, when comparing iron deficiency prevalence estimates based on BRINDA-adjusted ferritin and sTfR concentrations among PSC, there was more inconsistency between the 2 indicators (Figure 3B; Supplemental Table 7). For example, the 2 estimates were similar in Liberia-2011 (55.6% compared with 55.9%). However, in Côte d’Ivoire-2007 and Lao-2006, the prevalence based on adjusted ferritin was much higher than that based on adjusted sTfR (39.5% compared with 8.6% and 26.4% compared with 3.3%, respectively). In contrast, in Nepal-2016, the prevalence based on adjusted ferritin was lower (27.6%) than that based on adjusted sTfR (63.3%) (Supplemental Table 7). The 2 estimates tended to be more aligned among women in the few surveys in which both ferritin and sTfR were reported (Figure 2; Supplemental Table 8).

An additional methodological issue among WRA is that varying cutoffs were used by different national surveys to define low ferritin concentration ranging from <10 μg/L in Austria-2012 to <20 μg/L in Maldives-2008 (Supplemental Table 2a), with the majority of surveys using <15 μg/L, the cutoff recommended by the WHO for WRA [7]. There was less variation in the ferritin cutoffs used in surveys among PSC. Most used the recommended cutoff of <12 μg/L [7] with the exception of Nigeria-2001 and Maldives-2008, which used <10 and <20 μg/L, respectively (Supplemental Table 1a). The cutoff used to define elevated sTfR concentration also varied by survey ranging from >3.3 to >8.5 mg/L (Supplemental Tables 1b and 2b), which can be specific to the analytical method used in each survey. The majority of surveys and BRINDA used >8.3 mg/L to define iron-deficient erythropoiesis.

**FIGURE 4:**
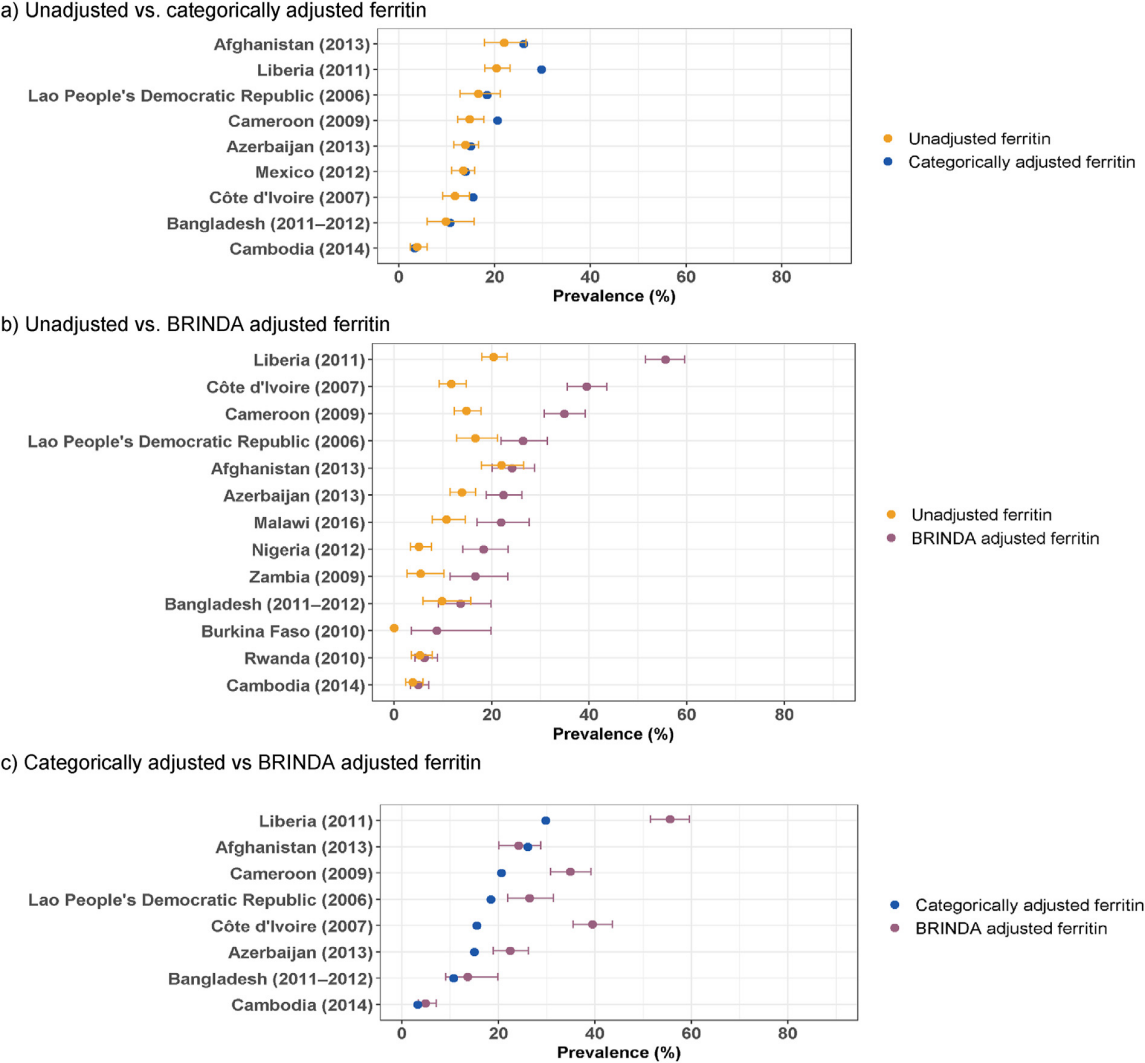
Comparison of iron deficiency prevalence among preschool-aged children from nationally representative surveys, estimated based on serum concentrations of ferritin adjusted for inflammation using different adjustment methods.^1^ BRINDA, Biomarkers Reflecting Inflammation and Nutritional Determinants of Anemia. ^1^ Results shown as prevalence (%) with 95% confidence interval, where available. Detailed results and methods for adjustment of inflammation are reported in Supplemental Table S7.

The micronutrient deficiency prevalence estimates reported in the WHO VMNIS Micronutrient Database [10] include a variety of approaches for dealing with inflammation, as summarized in Supplemental Tables 1–6. Older surveys often did not adjust for inflammation, excluded individuals with elevated indicators of inflammation, or used the categorical adjustments based on internal correction factors proposed by Thurnham et al. [26,27]. More recent surveys generally used the regression correction method proposed by BRINDA [[17], [18], [19], [20], [21]]. Whereas the typical downward adjustment of ferritin for inflammation increased the estimated prevalence of iron deficiency, adjustment of sTfR tended to reduce the estimated deficiency prevalence, both among PSC and WRA (Supplemental Tables 7 and 8). Among surveys for which data were available using more than one inflammation adjustment approach (e.g., categorical and BRINDA), it was possible to assess the effect of the different inflammation adjustment methods (Figure 4A–C). In some cases, the prevalence estimates were reasonably consistent regardless of the method used to adjust for inflammation. For example, in Afghanistan-2013, the prevalence of iron deficiency based on ferritin concentrations among PSC was 22.0% unadjusted, 24.2% when BRINDA-adjusted, and 26.1% when categorically adjusted. Similarly, the various approaches in Bangladesh-2011-2012 resulted in prevalence estimates among PSC ranging from 9.8% to 13.6% (Figure 4A–C; Supplemental Table 7). In contrast, in other surveys, the adjustment methods resulted in larger differences. For example, in Liberia-2011, the prevalence of iron deficiency among PSC based on ferritin concentration was 20.4% unadjusted, 29.8% adjusted categorically, and 55.6% adjusted using the BRINDA method. In this latter example, the extent to which iron deficiency would be considered a public health concern among PSC in Liberia would vary markedly depending on which result was applied. Similarly, inflammation adjustments of ferritin among WRA led to differences in the prevalence estimates of iron deficiency in some countries but not in others (Figure 2; Supplemental Table 8).

**FIGURE 5:**
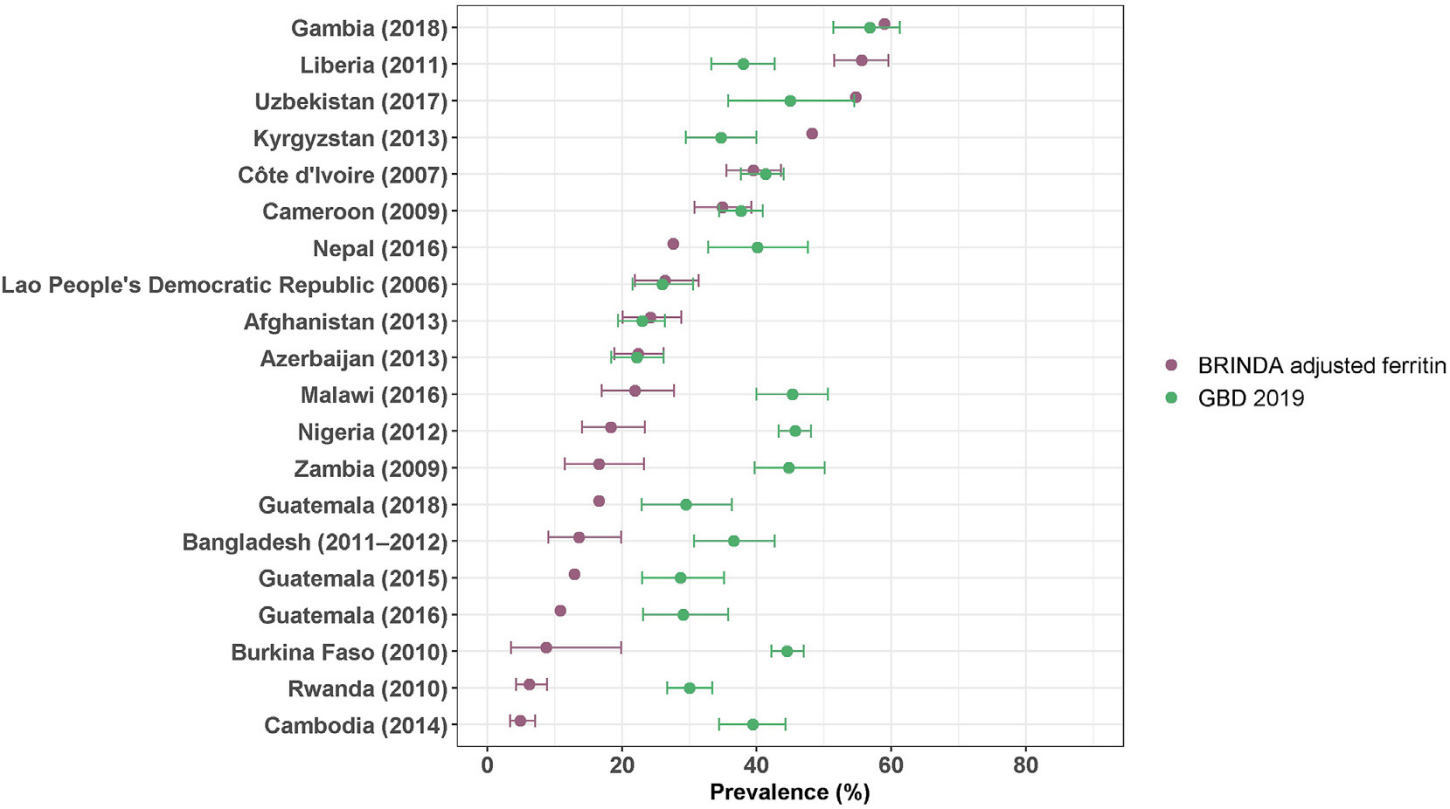
Comparison of iron deficiency prevalence among preschool-aged children from nationally representative surveys, estimated by BRINDA and for Global Burden of Disease 2019 Study.^1,2^ BRINDA, Biomarkers Reflecting Inflammation and Nutritional Determinants of Anemia; GBD, Global Burden of Disease Study. ^1^ Results shown as prevalence (%) with 95% confidence interval, where available. Detailed results and methods for adjustment of inflammation are reported in Supplemental Table S7. ^2^ Dietary iron deficiency estimated in the GBD 2019 Study for children 1 to 4 y of age as a cause of anemia, modeled based on hemoglobin concentration in the GBD 2019 Study [14]. These estimates represent only dietary iron deficiency associated with anemia and do not include iron deficiency without anemia.

In the GBD Study, the estimated prevalence of dietary iron deficiency is based on counterfactual modeling using hemoglobin as a proxy indicator modeled as a cause of anemia [14,16]. In other words, the GBD estimates for the prevalence of dietary iron deficiency are not based on dietary intake nor on ferritin or sTfR biomarker results and do not include iron deficiency without anemia. The dietary iron deficiency prevalence among children estimated for the GBD Study tended to be higher than the iron deficiency prevalence based on ferritin recorded in VMNIS, which had various inflammation adjustments applied (Figure 1). When comparing the dietary iron deficiency prevalence estimated in the GBD 2019 Study and the prevalence based on BRINDA-adjusted ferritin among children, there seems to be no consistent pattern (Figure 5; Supplemental Table 7). There is also inconsistency when comparing the estimated iron deficiency prevalence among WRA, unadjusted or adjusted (Figure 2; Supplemental Table 8), but in general, the GBD estimates for dietary iron deficiency are lower in relation to the VMNIS and BRINDA estimates among WRA than PSC.

### Prevalence of vitamin A deficiency

**FIGURE 6:**
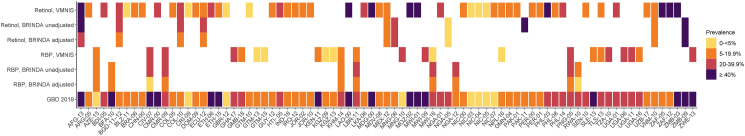
Prevalence of vitamin A deficiency among preschool-aged children in countries with nationally representative survey results as reported in VMNIS and by BRINDA and estimated for the GBD 2019 Study.^1^ BRINDA, Biomarkers Reflecting Inflammation and Nutritional Determinants of Anemia; GBD, Global Burden of Disease Study; RBP, retinol binding protein; VMNIS, Vitamin and Mineral Nutrition Information System. ^1^ Country survey codes, detailed results, and methods for adjustment of inflammation are reported in Supplemental Table S9.

**FIGURE 7:**
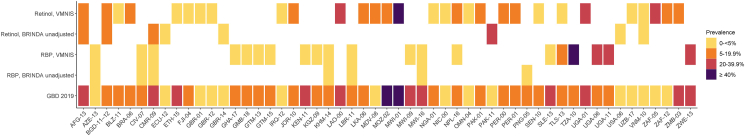
Prevalence of vitamin A deficiency among women of reproductive age in countries with nationally representative survey results as reported in VMNIS and by BRINDA and estimated for the GBD 2019 Study.^1^ BRINDA, Biomarkers Reflecting Inflammation and Nutritional Determinants of Anemia; GBD, Global Burden of Disease Study; RBP, retinol binding protein; VMNIS, Vitamin and Mineral Nutrition Information System. ^1^ Country survey codes, detailed results, and methods for adjustment of inflammation are reported in Supplemental Table S10.

Depending on the method used to determine the vitamin A prevalence, the prevalence estimates varied in many countries for both PSC and WRA (FIGURE 6, FIGURE 7). To assess vitamin A status, retinol and RBP were both commonly used in nationally representative surveys. Some surveys assessed RBP in all survey participants, and retinol in a small subsample to allow for adjustments of RBP in relation to recognized retinol cutoffs. However, unlike with ferritin and sTfR, both of which are often available for the full sample in the same survey, vitamin A is more commonly assessed by either retinol or RBP, and thus the prevalence of vitamin A deficiency was estimated based on only one of these biomarkers.

The cutoff used to define vitamin A deficiency among PSC and WRA was consistent at <0.7 μmol/L for retinol [8] (Supplemental Tables 3a and S4a), with a bit more variation for RBP ranging from <0.46 to <0.825 μmol/L among PSC (Supplemental Table 3b) and <0.46 to <1.24 μmol/L among WRA (Supplemental Table 4b), likely because some surveys determined the RBP equivalent of a retinol concentration of 0.7 μmol/L by analyzing both in a subsample.

**FIGURE 8:**
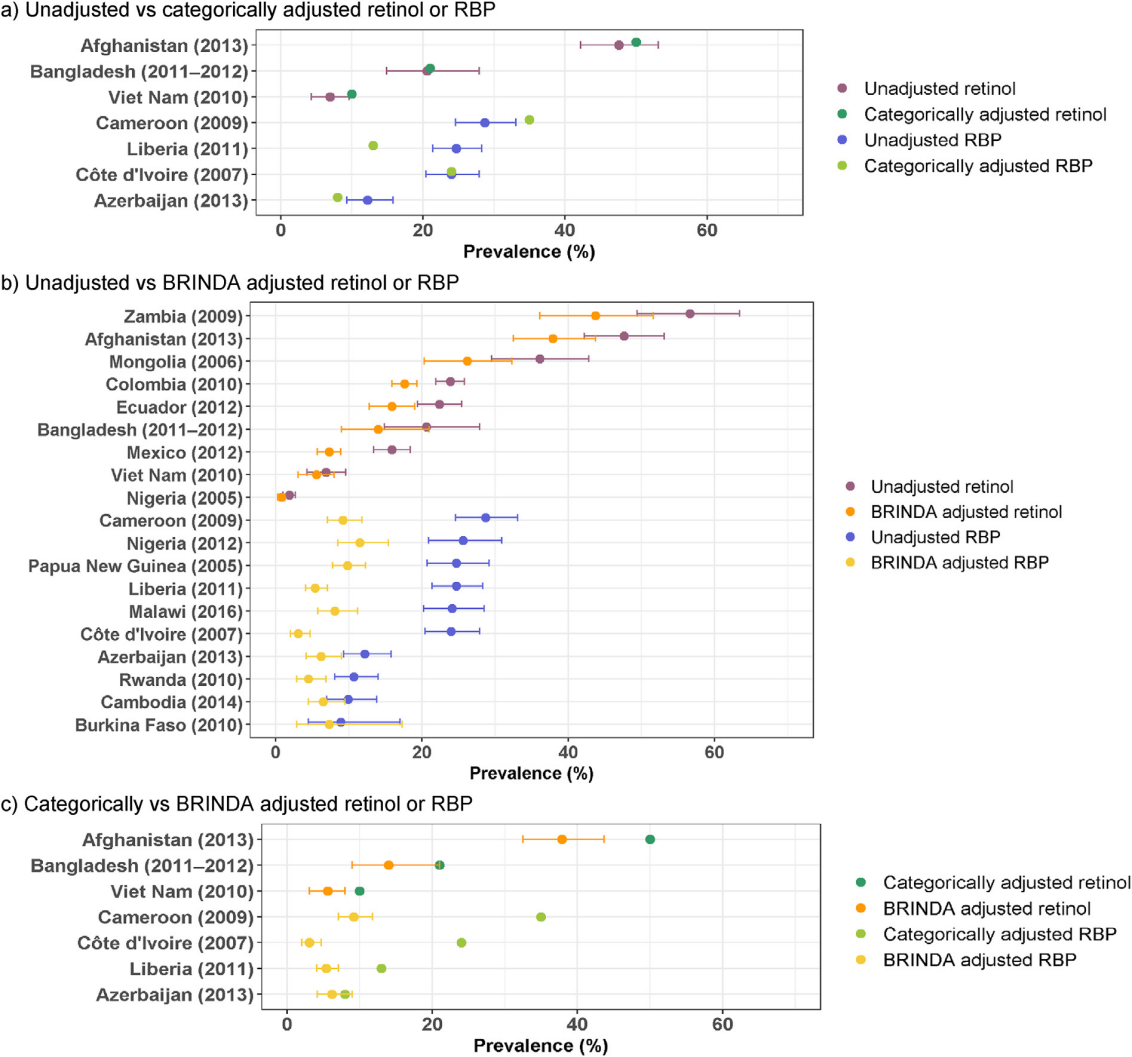
Comparison of vitamin A deficiency prevalence among preschool-aged children from nationally representative surveys, estimated by using different biomarkers of vitamin A status and different methods to adjust for inflammation.^1^ BRINDA, Biomarkers Reflecting Inflammation and Nutritional Determinants of Anemia. ^1^ Results shown as prevalence (%) with 95% confidence interval, where available. Detailed results and methods for adjustment of inflammation are reported in Supplemental Table S9.

Similar to iron deficiency, the prevalence of vitamin A deficiency reported in the VMNIS was either unadjusted or adjusted for inflammation using various strategies (Supplemental Tables 3a–4b). As retinol and RBP are lower during inflammation, adjustments for inflammation result in a lower prevalence of vitamin A deficiency (Figure 8A, B), and this is more apparent with the BRINDA adjustments compared to categorical adjustments (Figure 8C). For example, in the Mexico-2012 survey, the unadjusted prevalence of vitamin A deficiency among PSC was 16% (Supplemental Table 9). After BRINDA adjustment of the retinol concentration, the estimated prevalence of vitamin A deficiency fell to 7.3%, although both prevalence estimates suggest that vitamin A deficiency was not a severe public health problem in Mexico in 2012 (Figure 6). BRINDA recommends adjustments of vitamin A markers for inflammation only for PSC and not WRA [18,21]. Among WRA, the vitamin A prevalence reported in VMNIS (unadjusted or adjusted) and the unadjusted prevalence reported in BRINDA are similar (Figure 7; Supplemental Table 10).

**FIGURE 9:**
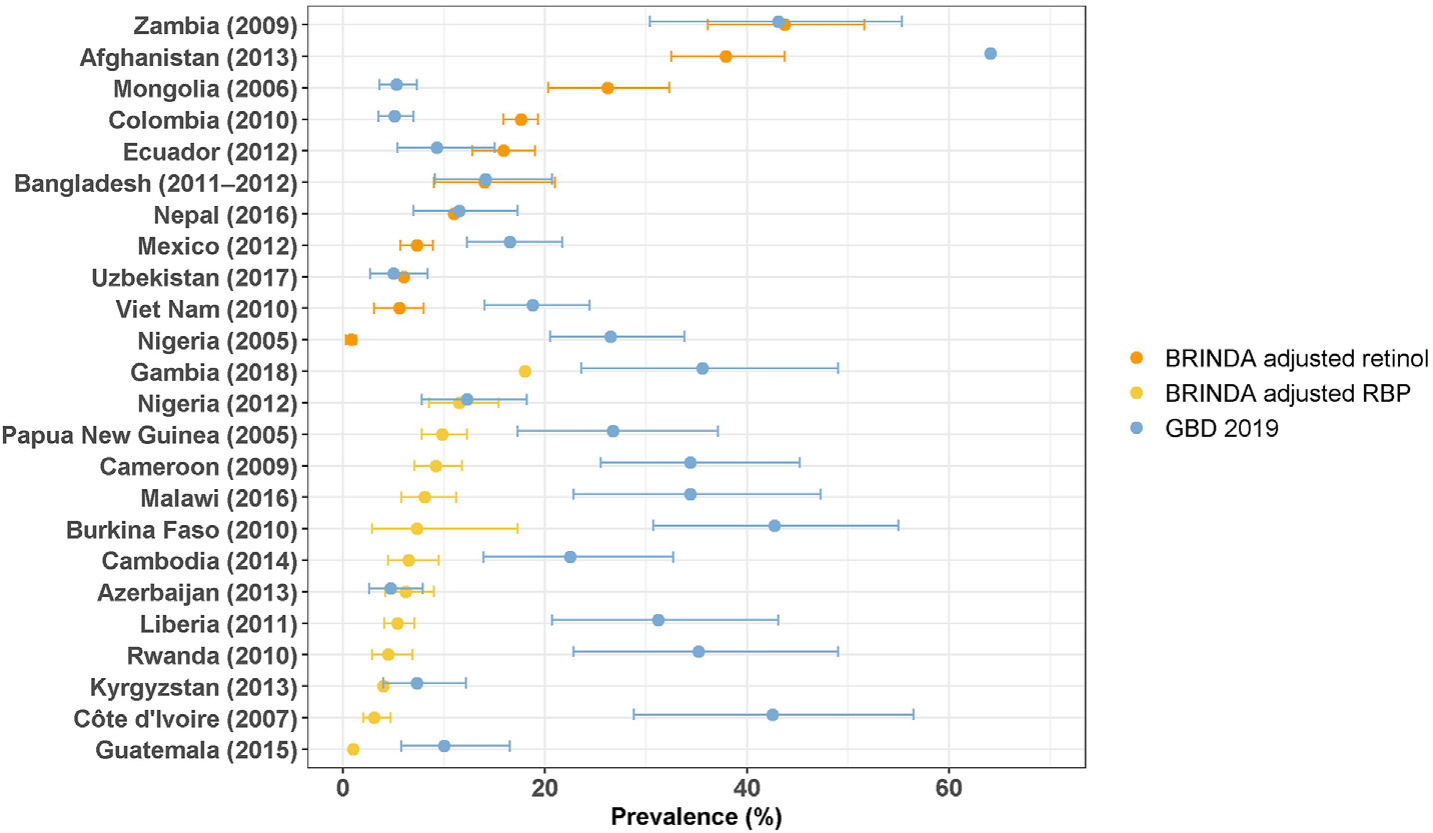
Comparison of vitamin A deficiency prevalence among preschool-aged children from nationally representative surveys, estimated by BRINDA and the Global Burden of Disease Study 2019.^1,2^ ^1^ Results shown as prevalence (%) with 95% confidence interval, where available. Detailed results and methods for adjustment of inflammation are reported in Supplemental Table S9. ^2^ The primary source of vitamin A deficiency data for the GBD 2019 Study was from the WHO VMNIS database. The GBD 2019 Study used the spatiotemporal Gaussian process regression (ST-GPR) model to estimate prevalence for each year and location [15].

The GBD 2019 Study uses retinol concentration reported in VMNIS for estimating the prevalence of vitamin A deficiency and thus reports a similar prevalence for some country-years with retinol concentration (Figure 9). However, because GBD uses retinol and not RBP data in their models, when RBP was the biomarker assessed in the nationally representative survey, there is no consistency in the prevalence of vitamin A deficiency reported in VMNIS or by BRINDA and estimated in the GBD 2019 Study among PSC and WRA (Figure 9; Supplemental Tables 9 and 10).

### Prevalence of zinc deficiency

**FIGURE 10:**
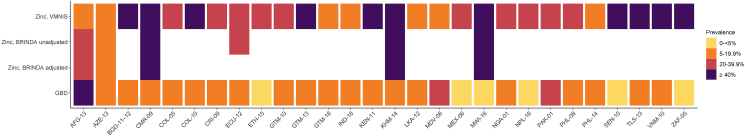
Prevalence of zinc deficiency among preschool-aged children in countries with nationally representative survey results as reported in VMNIS and by BRINDA and estimated for the GBD 2019 Study.^1^ BRINDA, Biomarkers Reflecting Inflammation and Nutritional Determinants of Anemia; GBD, Global Burden of Disease Study; VMNIS, Vitamin and Mineral Nutrition Information System. ^1^ Country survey codes, detailed results, and methods for adjustment of inflammation are reported in Supplemental Table S11.

With few exceptions, the prevalence estimates for zinc deficiency varied markedly for most country-years depending on the methods used to estimate the prevalence (Figure 10). For zinc deficiency, a potential cause of discrepancy was the use of different cutoffs, although most but not all surveys used cutoffs recommended by the International Zinc Nutrition Consultative Group (IZiNCG) [32] (Supplemental Tables 5 and 6). The only survey among PSC in which a direct comparison of the cutoff effect is possible is Afghanistan-2013, which used a cutoff <9.2 μmol/L, as compared with <9.9 μmol/L as recommended by IZiNCG and used by BRINDA. Consequently, the prevalence of zinc deficiency among PSC based on the lower cutoff in the VMNIS is lower (15.1%, adjusted categorically) compared with the prevalence estimates by BRINDA (25.5% unadjusted: 21.4% BRINDA-adjusted).

**FIGURE 11:**
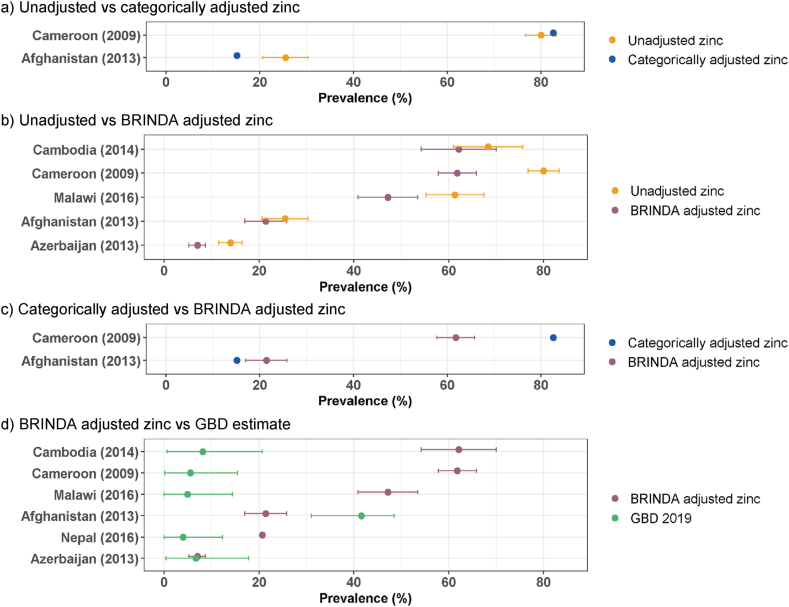
Comparison of zinc deficiency prevalence among preschool-aged children from nationally representative surveys, estimated by using different methodological approaches.^1,2^ BRINDA, Biomarkers Reflecting Inflammation and Nutritional Determinants of Anemia; GBD, Global Burden of Disease Study. ^1^ Results shown as prevalence (%) with 95% confidence interval, where available. Detailed results and methods for adjustment of inflammation are reported in Supplemental Table S11 ^2^ The prevalence of zinc deficiency among children 1 to 4 y of age in the GBD 2019 Study is estimated based on dietary intake data from nationally and subnationally representative nutrition surveys and from food availability data obtained from FAO supply utilization accounts (after adjusting for food waste) [15]. This information was then used to predict the mean zinc intake at the population level, and to characterize the distribution of zinc intake, as a proxy for zinc status. GBD 2019 Study used the spatiotemporal Gaussian process regression (ST-GPR) model to estimate for each year and location [15].

As with the other biomarkers reported in VMNIS, the estimated prevalence of zinc deficiency in VMNIS is based on unadjusted and adjusted plasma zinc concentrations, depending on how the results were reported in individual survey documents and using different approaches for the adjustments. Adjusting for inflammation typically reduced the prevalence of zinc deficiency. For example, among PSC in Cameroon-2009, the unadjusted prevalence was 80.0%, the categorically-adjusted prevalence was 82.6%, and the BRINDA-adjusted prevalence was 61.8% (Figure 11A–C; Supplemental Table 11). In the case of Cameroon-2009, all estimates were so high that the public health concern would be considered severe regardless of the adjustment strategy (Figure 10).

The GBD 2019 Study modeled the prevalence of zinc deficiency among PSC based on zinc availability in the national food supply after cross-walking it into 24-h dietary intake using "meta-regression-Bayesian, regularized, trimmed", and there seems no consistent pattern between the GBD estimated prevalence and the prevalence based on plasma zinc concentration. In Afghanistan-2013, the prevalence of low plasma zinc concentration ranged from 15.1% to 25.5% depending on cutoffs and inflammation adjustments, whereas the GBD estimate was much higher at 41.6% (Figure 11A–D; Supplemental Table 11). However, for most other country-years, the modeled zinc deficiency prevalence in the GBD 2019 Study was substantially lower than the prevalence based on plasma zinc concentration (FIGURE 10, FIGURE 11D).

Twenty-two surveys reported plasma zinc concentration among WRA (Supplemental Table 12), but statistical adjustments for inflammation are not recommended for WRA [20], and the GBD Study does not model the global burden of zinc deficiency among women. Thus, there are no other published national prevalence estimates for zinc deficiency specific to WRA.

We identified only 3 nationally representative surveys that reported on iron, vitamin A, and zinc status using biomarkers and also assessed dietary intakes of these same micronutrients (Supplemental Table 13). The respective prevalence estimates of deficiency versus dietary inadequacy varied greatly (Supplemental Tables 7–12). For example, in Kenya, the prevalence of low ferritin concentration (categorically inflammation-adjusted) among PSC was 21.8%, and the prevalence of inadequate dietary iron intake was 67%. In Mexico-2012, the prevalence estimates were more comparable with 13.9% of PSC having low categorically-adjusted ferritin and 4.8% low dietary iron intake. As previously reported by Engle-Stone et al. [33] for Cameroon-2009, the prevalence of low zinc deficiency was very high among PSC compared with a much lower prevalence of inadequate dietary zinc intake (82.6% compared with 19.1%), although the latter varies greatly depending on the nutrient reference value applied and the assumed bioavailability [34]. Similar differences in results were noted among WRA in Cameroon.

## Discussion

In the present review, we summarize published prevalence estimates from nationally representative surveys for iron, vitamin A, and zinc deficiency for PSC and WRA as reported in different publicly available data archives. We found that the estimates vary, depending on numerous methodological factors, such as *1*) whether the prevalence was estimated based on the assessment of a biomarker, dietary intake, or proxy indicators, *2*) which biomarker and what cutoffs were used, *3*) whether the biomarker was adjusted for inflammation and what adjustment method was applied. For some country-years, the various approaches result in fairly consistent prevalence estimates. For other country-years, however, the results differ markedly and change conclusions regarding the existence and extent of a deficiency as a public health concern. Inconsistencies between estimates are particularly striking when proxy indicators were used to fill gaps in available biomarker information. Because the different approaches currently being used provide inconsistent prevalence estimates, efforts are needed to develop consensus on best practices for analyzing and interpreting available information and to harmonize the results.

Many national surveys included in the present review used ferritin alone or ferritin and sTfR to determine the prevalence of iron deficiency. While ferritin is an indicator of storage iron depletion, sTfR is a biomarker of iron-deficient erythropoiesis [4]. In cases of true nutritional iron deficiency, one would expect the prevalence of low iron stores to be greater than the prevalence of iron-deficient erythropoiesis. However, iron metabolism and erythropoiesis are affected by factors other than just iron deficiency (e.g., malaria, other micronutrients, hemoglobinopathies), and thus among the surveys that assessed both ferritin and sTfR, the prevalence estimates based on the 2 indicators were inconsistent. More recently, WHO recommended ferritin as the preferred method to assess iron deficiency, along with adjustments for inflammation [7].

WHO recommends using serum retinol concentration to assess vitamin A deficiency [8]. Retinol is released from the liver with its carrier protein RBP, thus serum RBP correlates closely with serum retinol concentration [5]. Because RBP is the less costly biomarker, many national nutrition surveys used RBP concentration as their vitamin A indicator. However, there is variability in the molar ratio of retinol and RBP across populations [5]. Thus, when using RBP to determine the prevalence of vitamin A deficiency, it is recommended to analyze serum retinol concentration in a subset of samples and to predict retinol from RBP [5]. Although some national surveys have used this approach, it was not consistently reported.

Since the biomarkers of iron, vitamin A and zinc status are affected by the presence of infection, injury, or systemic inflammation [[4], [5], [6], [7], [8]], we further consider that adjustment for inflammation is desirable, when present. As previously reported by BRINDA, not adjusting for inflammation may result in underestimation of the prevalence of iron deficiency and overestimation of the prevalence of vitamin A and zinc deficiencies, especially among PSC and to a lesser extent among WRA [20,21]. The use of one approach across surveys has the advantage of allowing comparison of prevalence estimates across countries and years. In recent years, most surveys adopted the regression correction, also known as the BRINDA approach [12], the strengths and weaknesses of which have been discussed in depth elsewhere [11,[17], [18], [19], [20], [21],^35^]. BRINDA has developed a simple, publicly available tool to adjust datasets for inflammation [12], which will help harmonize result presentation and interpretation across surveys. As recommended by WHO, unadjusted results should also be reported to allow for comparison with unadjusted values from surveys that did not measure markers of inflammation [7].

An important factor in the interpretation of biomarker results is consideration of the potential effects of preanalytical factors [35]. Special precautions are needed to avoid contamination of serum or plasma samples with zinc [36] and degradation of retinol by ultraviolet light. Although the majority of surveys analyzed serum or plasma samples for ferritin, sTfR, RBP, CRP, and AGP at the VitMin Lab (Willstaet, Germany) [10], various methods were used to determine plasma zinc. This could contribute to some of the variation, as a recent comparison of plasma zinc analyses in specialized laboratories found considerable interlaboratory differences despite good within-laboratory precision [37]. External quality assessment is available for status indicators for vitamin A, iron, and other micronutrients but not zinc [38,39]. The lack of an external quality assurance program for zinc has previously been identified as a need to improve standardization and accuracy [3].

The selection of biomarkers in a national survey is a complex process involving considerations of potential public health concern and weighing that against available resources [40]. Over the 20-y period from 2000 to 2020, we found that 59 countries measured biomarkers of iron and/or vitamin A status for PSC and WRA, and only 26 countries collected information on zinc status. We are reaffirming previous calls urging for more data collection on micronutrient status [3,16]. We searched for published survey results, so more recent national surveys may not yet have been published and were therefore not included in the present review. This is unlikely to have changed the conclusions of this manuscript as the focus was on methodological considerations, and we believe we achieved the primary objective of the present review, which was to provide a side-by-side comparison of various published estimates and indicate possible sources of inconsistencies in these estimates. We have attempted to review the main considerations that may affect the prevalence estimates. Additional variations may derive from different sampling weights used and different considerations in inclusion/exclusion criteria for samples in the analysis of the results presented in VMNIS compared with BRINDA.

Because of the lack of nationally representative micronutrient data and a desire to provide deficiency prevalence estimates for all countries, the GBD Study relies on proxy indicators to estimate the prevalence of the key micronutrient deficiencies and extrapolate data across countries and time. For iron deficiency, the GBD Study uses counterfactual modeling based on hemoglobin with an attempt to model iron deficiency anemia that is due only to inadequate iron intake [14,16]. The estimated prevalence of dietary iron deficiency in the GBD 2019 Study seems to be higher for PSC and lower for WRA compared to the prevalence reported in VMNIS, although there is some inconsistency. Lastly, the estimated prevalence of zinc deficiency among young children modeled in the GBD 2019 Study based on inadequate zinc availability in the national food supply after cross-walking it into 24-h dietary intake was substantially lower than the prevalence of zinc deficiency based on plasma zinc concentration for most country-years. This is in agreement with a previous report, which found that the prevalence of inadequate dietary zinc availability based on food balance sheets underestimates the prevalence of zinc deficiency compared to plasma zinc concentration [41]. Our overall conclusion remains that estimates based on biomarkers of micronutrient status are more reliable than those relying on proxies.

Despite reaching out to various dietary assessment expert groups, we were able to identify only limited information on inadequate dietary intake of iron, vitamin A, and zinc intake from nationally representative surveys that also included concurrent biomarker assessment of these micronutrients. Despite the limited overlap in available results, the surveys that were identified confirmed that the prevalence of deficiency and the prevalence of inadequate dietary intake are not equivalent, as has been shown previously for the NHANES survey [42,43]. Although micronutrient deficiencies are in part caused by inadequate dietary intake, biomarkers are under homeostatic control and are affected by inflammation and many other factors [[4], [5], [6],^44^]. Dietary intake of micronutrients may vary by season, may be consumed from unmeasured sources such as supplements, ambient water, and soil contamination of foods, or produced by gut flora or fermentation of food [16]. Moreover, estimating dietary intake faces several methodological challenges [34,45,46].

The severity of a public health problem related to micronutrient deficiencies can be defined by 2 distinct parameters: the percentage of individuals who are affected and the degree of the deficiency. There is no consensus on how to classify the severity of the public health problem for each key nutrient based on these parameters. For zinc, IZiNCG suggests that when ≥20% of the population have low plasma zinc concentration and/or ≥25% of the population have zinc intakes less than the estimated average requirement, risk of zinc deficiency is elevated and of public health concern [32,47]. To determine the extent of vitamin A deficiency as a public health concern, retinol concentration should be used in conjunction with another biological indicator or in consideration of other risk factors such as infant mortality [5]. For anemia and iron deficiency, the WHO proposes to consider <5% as no public health problem, 5% to 19.9% as mild, 20% to 39.9% as moderate, and ≥40% as severe public health problem [7,30]. Thus, for the present review, we have chosen these categories for visualization purposes and not to suggest a degree of public health concern across all micronutrient deficiencies presented here. However, even without formal recommendations to define public health burden of vitamin A and zinc deficiencies, large variation in the prevalence estimates can cause confusion, which may hinder action to prevent the deficiency.

In conclusion, the lack of micronutrient status data from representative surveys is a major limitation to determine the extent of micronutrient deficiencies. Because of the limited available information from nationally representative surveys, proxies may have a purpose to fill the gap, but more research is needed to improve prevalence estimates relying on proxies. To determine micronutrient status, we consider the assessment of one or more of the recommended biomarkers in a representative population survey as the best available information. If indicated, results should be adjusted for inflammation. Although multiple approaches are available to adjust for inflammation, the use of the BRINDA approach across surveys would have the advantage to allow comparison of prevalence estimates across countries and years. Consensus exists on appropriate biomarkers (serum or plasma ferritin, retinol, and zinc, respectively, for iron, vitamin A, and zinc deficiency), but there is a need for consensus on best practices with regard to biomarker cutoffs to define deficiency, methods to adjust for inflammation, and defining both the severity and extent of individual micronutrient deficiencies.

### Author contributions

The authors’ responsibilities were as follows—SYH: designed and conducted review; DH: provided GBD results; XT: visualized results; SYH, KHB: wrote the paper; and all authors: read, critically edited, and approved the final manuscript.

### Conflict of interest

JG works for the Bill & Melinda Gates Foundation, MB works for the Micronutrient Forum, and KHB, the spouse of SYH, works as a consultant for the Micronutrient Forum and the Bill & Melinda Gates Foundation. All other authors report no conflicts of interest.

### Funding

This work was supported, in part, by the Bill & Melinda Gates Foundation with a grant to the Micronutrient Forum (INV-003021). Under the grant conditions of the Foundation, a Creative Commons Attribution 4.0 Generic License has already been assigned to the Author Accepted Manuscript version that might arise from this submission. The funder had no role in the present review, except for JG, an employee of the Bill & Melinda Gates Foundation, who contributed to editing the manuscript.

### Disclaimer

The authors alone are responsible for the views expressed in this article and they do not necessarily represent the views, decisions or policies of the institutions with which they are affiliated.

## Appendix A Supplementary data

Multimedia component1 Multimedia component1

Multimedia component2 Multimedia component2

Multimedia component3 Multimedia component3
