# Supplementary material for: Comparison of Published Estimates of the National Prevalence of Iron, Vitamin A, and Zinc Deficiency and Sources of Inconsistencies
Source: Adv Nutr. 2023 Aug 25;14(6):1466–78. doi: 10.1016/j.advnut.2023.08.011 (PMC10721533; doi:10.1016/j.advnut.2023.08.011)
Supplement: Multimedia component1 [file mmc1.pdf]

Comparison of published estimates of the national prevalence of iron, vitamin A and zinc deficiency and sources of inconsistencies

Supplemental Tables S1 – S6

by Hess SY, Wessells KR, Haile D, Rogers LM, Tan X, Barros JG, Bourassa MW, Gorstein J, Brown KH

	Page
**Supplemental Table S1a.** Overview of methods used in nationally representative surveys with data on iron deficiency based on serum or plasma ferritin among young children implemented between 2000 to 2019 and reported in the WHO's micronutrients database VMNIS	2
**Supplemental Table S1b.** Overview of nationally representative surveys with data on iron deficiency based on soluble transferrin receptor among young children implemented between 2000 to 2019 and reported in the WHO's micronutrients database VMNIS	6
**Supplemental Table S2a.** Overview of nationally representative surveys with data on iron deficiency based on serum or plasma ferritin among women of reproductive age implemented between 2000 to 2019 and reported in the WHO's micronutrients database VMNIS	8
**Supplemental Table S2b.** Overview of nationally representative surveys with data on iron deficiency based on soluble transferrin receptor among women of reproductive age implemented between 2000 to 2019 and reported in the WHO's micronutrients database VMNIS	12
**Supplemental Table S3a.** Overview of nationally representative surveys with data on vitamin A deficiency based on serum or plasma retinol among young children implemented between 2000 to 2019 and reported in the WHO's micronutrients database VMNIS	14
**Supplemental Table S3b.** Overview of nationally representative surveys with data on vitamin A deficiency based on serum or plasma retinol binding protein among young children implemented between 2000 to 2019 and reported in the WHO's micronutrients database VMNIS	17
**Supplemental Table S4a.** Overview of nationally representative surveys with data on vitamin A deficiency based on serum or plasma retinol among women of reproductive age implemented between 2000 to 2019 and reported in the WHO's micronutrients database VMNIS	19
**Supplemental Table S4b.** Overview of nationally representative surveys with data on vitamin A deficiency based on serum or plasma retinol binding protein among women of reproductive age implemented between 2000 to 2019 and reported in the WHO's micronutrients database VMNIS	21
**Supplemental Table S5.** Overview of nationally representative surveys with data on zinc deficiency based on serum or plasma zinc among young children implemented between 2000 to 2019 and reported in the WHO's micronutrients database VMNIS	23
**Supplemental Table S6.** Overview of nationally representative surveys with data on zinc deficiency based on serum or plasma zinc among women of reproductive age implemented between 2000 to 2019 and reported in the WHO's micronutrients database VMNIS	25

Supplemental Table S1a. Overview of methods used in nationally representative surveys with data on iron deficiency based on serum or plasma ferritin among young children implemented between 2000 to 2019 and reported in the WHO's micronutrients database VMNIS[1]

Survey (Year)[2]	Population	Age Range (mo)	*n*	Depleted Iron Stores Cut-Off (μg/L)	Depleted Iron Stores Prevalence (%)	Sample Type	Method of Analysis	Adjustment for Inflammation[3]
Afghanistan (2013)	PSC	6–59	Not Specified	<12	26.1	Serum	Not Specified	Categorical for elevated CRP and AGP
Argentina (2005)	PSC	6–23	Not Specified	<12	35.3	Serum	EIA, ELISA	Ferritin cutoff < 30 μg/L when leukocyte count > 15 x 1000 mm^3
Azerbaijan (2013)	PSC	6–59	1111	<12	15	Plasma	EIA, ELISA	Categorical for elevated CRP and AGP
Bangladesh (2011)	PSC	6–59	468	<12	10.7	Serum	EIA, ELISA	Categorical for elevated CRP and AGP
Belize (2011)	PSC	6–59	636	<12	13.2	Plasma	EIA, ELISA	Samples with AGP > 1 g/L excluded
Cambodia (2014)	PSC	6–71	793	<12	3.3	Plasma	EIA, ELISA	Categorical for elevated CRP and AGP
Cameroon (2009)	PSC	12–59	838	<12	20.6	Plasma	EIA, ELISA	Categorical for elevated CRP and AGP
Canada (2011)	PSC	36–60	487	<12	3.2	Serum	ECLIA	Not specified
Colombia (2005)	PSC	12–48	4188	<12	12.5	Plasma	ECLIA	Samples with CRP 12 mg/L were excluded
Colombia (2010)	PSC	12–48	3542	<12	10.6	Plasma	ECLIA	Samples with CRP 12 mg/L were excluded
Costa Rica (2009)	PSC	12–72	399	<12	5.9	Serum	EIA, ELISA	Not specified
Côte d'Ivoire (2007)	PSC	6–59	783	<12	15.5	Plasma	EIA, ELISA	Categorical for elevated CRP and AGP
Dominican Republic (2009)	PSC	6–59	330	<12	27.4	Plasma	EIA, ELISA	Samples with AGP > 1 g/L excluded
Ecuador (2012)	PSC	6–59	2045	<12	9.9	Serum	ECLIA	Unadjusted, also presented w/o inflammation
Ethiopia (2015)	PSC	6–59	1140	<12	17.8	Serum	ITA	Categorical for elevated CRP and AGP

Supplemental Table S1a, continued

Survey (Year)[2]	Population	Age Range (mo)	*n*	Depleted Iron Stores Cut-Off (µg/L)	Depleted Iron Stores Prevalence (%)	Sample Type	Method of Analysis	Adjustment for Inflammation[3]
Gambia (2018)	PSC	6–59	1012	<12	59	Serum	EIA, ELISA	BRINDA using CRP and AGP
Georgia (2009)	PSC	12–59	1648	<12	0.1	Serum	EIA, ELISA	Samples with CRP > 5 mg/L excluded
Ghana (2017)	PSC	6–59	1165	<12	21.5	Serum	EIA, ELISA	Categorical for elevated CRP and AGP
Guatemala (2010)	PSC	6–59	985	<12	18.6	Serum	EIA, ELISA	Unadjusted, also presented with ferritin cutoff > 30 µg/L when AGP > 1 g/L
Guatemala (2013)	PSC	6–59	858	<12	8.9	Plasma	EIA, ELISA	Categorical for elevated CRP and AGP
Guatemala (2015)	PSC	6–59	682	<12	12.9	Plasma	EIA, ELISA	BRINDA using CRP and AGP
Guatemala (2016)	PSC	6–59	569	<12	10.8	Plasma	EIA, ELISA	BRINDA using CRP and AGP
Guatemala (2018)	PSC	6–59	551	<12	16.6	Plasma	EIA, ELISA	BRINDA using CRP and AGP
India (2018)	PSC	12–48	7838	<12	31.9	Serum	EIA, ELISA	Samples with CRP > 5 mg/L excluded
Iraq (2012)	PSC	12–59	2087	<12	14.4	Serum	RIA	Not specified
Jordan (2002)	PSC	12–59	1066	<12	26.1	Serum	ECLIA	Not specified
Jordan (2010)	PSC	12–59	940	<12	13.7	Serum	ECLIA	Unadjusted
Kenya (2011)	PSC	6–59	918	<12	21.8	Serum	Not Specified	Categorical for elevated CRP and AGP
Kyrgyzstan (2009)	PSC	6–59	1413	<12	44.6	Plasma	EIA, ELISA	Excluded samples with elevated CRP and/or AGP
Kyrgyzstan (2013)	PSC	6–29	2148	<12	48.2	Plasma	EIA, ELISA	BRINDA using CRP and AGP
Lao People's Democratic Republic (2006)	PSC	6–59	483	<12	18.4	Plasma	EIA, ELISA	Categorical for elevated CRP and AGP
Liberia (2011)	PSC	6–35	1416	<12	29.8	Serum	EIA, ELISA	Categorical for elevated CRP and AGP

Supplemental Table S1a, continued

Survey (Year)[2]	Population	Age Range (mo)	*n*	Depleted Iron Stores Cut-Off (µg/L)	Depleted Iron Stores Prevalence (%)	Sample Type	Method of Analysis	Adjustment for Inflammation[3]
Malawi (2009)	PSC	6–59	455	<12	10.3	Serum	EIA, ELISA	Samples with CRP > 5 mg/L or AGP > 1 g/L excluded
Malawi (2016)	PSC	6–59	1102	<12	21.7	Serum	EIA, ELISA	BRINDA using CRP and AGP
Maldives (2008)	PSC	0–60	1268	<20	57.3	Serum	EIA, ELISA	Unadjusted, also presented adjusted for CRP > 65 mg/L
Mexico (2006)	PSC	12–48	1710	<12	26	Serum	EIA, ELISA	Not specified
Mexico (2012)	PSC	12–59	2591	<12	13.9	Serum	ECLIA	Categorical for elevated CRP
Mongolia (2004)	PSC	6–59	386	<12	22.3	Serum	Not Specified	Not specified
Mongolia (2010)	PSC	6–59	433	<12	21.4	Serum	EIA, ELISA	Samples with elevated CRP excluded
Morocco (2000)	PSC	6–59	644	<12	50.8	Serum	EIA, ELISA	Not specified
Nepal (2016)	PSC	6–59	1651	<12	27.6	Plasma	EIA, ELISA	BRINDA using CRP and AGP
Nicaragua (2004)	PSC	6–59	345	<12	32.6	Serum	EIA, ELISA	Samples with AGP > 1 g/L excluded
Nicaragua (2005)	PSC	6–59	368	<12	42.2	Serum	EIA, ELISA	Samples with AGP > 1 g/L excluded
Nicaragua (2007)	PSC	6–59	Not Specified	<12	45.1	Serum	EIA, ELISA	Samples with AGP > 1 g/L excluded
Nigeria (2001)	PSC	6–59	3091	<10	19.4	Serum	RIA	Not specified
Oman (2004)	PSC	6–59	199	<12	18.5	Serum	ECLIA	Samples with CRP > 10 mg/L excluded
Pakistan (2001)	PSC	6–59	5594	<12	66.5	Serum	ECLIA	Not specified
Senegal (2010)	PSC	12–59	1431	<12	63.2	Plasma	EIA, ELISA	Categorical for elevated CRP and AGP
Sierra Leone (2013)	PSC	6–59	654	<12	5.2	Plasma	EIA, ELISA	Categorical for elevated CRP and AGP
South Africa (2005)	PSC	12–60	821	<12	19.7	Serum	PENIA	Not specified
South Africa (2012)	PSC	0–59	454	<12	10	Serum	EIA, ELISA	Not specified
Sri Lanka (2012)	PSC	6–59	5766	<12	33.6	Serum	EIA, ELISA	Samples with CRP > 5 mg/L excluded

Supplemental Table S1a, continued

Survey (Year)[2]	Population	Age Range (mo)	*n*	Depleted Iron Stores Cut-Off (µg/L)	Depleted Iron Stores Prevalence (%)	Sample Type	Method of Analysis	Adjustment for Inflammation[3]
Tajikistan (2009)	PSC	6–59	1805	<12	9.7	Serum	EIA, ELISA	Samples with CRP > 5 mg/L excluded
Timor-Leste (2013)	PSC	6–59	547	<12	20.5	Serum	EIA, ELISA	Categorical for elevated CRP and AGP
United Kingdom of Great Britain and Northern (2012)	PSC	12–36	43	<12	35.1	Plasma	PENIA	Not specified
United Kingdom of Great Britain and Northern (2014)	PSC	12–36	31	<12	31	Plasma	PENIA	Not specified
Uzbekistan (2017)	PSC	6–59	1736	<12	54.7	Serum	ECLIA	BRINDA using CRP and AGP
Viet Nam (2010)	PSC	10–75	568	<12	12.9	Plasma	EIA, ELISA	Categorical for elevated CRP

Abbreviations: AGP, α 1-acid-glycoprotein; CRP, C-reactive protein; ECLIA, electrochemiluminescence immunoassay; EIA, ELISA; enzyme immunoassay, enzyme-linked immunosorbent assay; ITA, immunoturbidimetric assay; PENIA, particle-enhanced nephelometric immunoassay; PSC, preschool-age children; RIA, radioimmunoassay; VMNIS, Vitamin and Mineral Nutrition Information System; WHO, World Health Organization

[1] Downloaded from VMNIS on March 29, 2022
[2] Reported from the 'Date' column of the VMNIS database, ranging from 2000–2019.
[3] Adjustment for inflammation summarized based on information under 'Indicator Comments' in the VMNIS database. Categorical adjustments refer to the method using internal correction factor(s) suggested by Thurnham et al. (2005; 2010) and BRINDA refers to adjustments using linear regression proposed by the Biomarkers Reflecting Inflammation and Nutritional Determinants of Anemia (BRINDA) project (Namaste et al., 2017).

Supplemental Table S1b. Overview of nationally representative surveys with data on iron deficiency based on soluble transferrin receptor among young children implemented between 2000 to 2019 and reported in the WHO's micronutrients database VMNIS[1]

Survey (Year)[2]	Population	Age Range (mo)	*n*	Inadequacy Threshold (mg/L)	Prevalence of Elevated sTfR (%)	Sample Type	Method of Analysis	Adjustment for Inflammation[3]
Cambodia (2014)	PSC	6–71	793	>8.3	47.5	Plasma	EIA, ELISA	Not specified
Cameroon (2009)	PSC	12–59	838	>8.3	68.4	Plasma	EIA, ELISA	Unadjusted
Côte d'Ivoire (2007)	PSC	6–59	783	>8.3	11.2	Plasma	EIA, ELISA	Not specified
Ethiopia (2015)	PSC	6–59	1138	>4.4	29.6	Serum	EIA, ELISA	Not specified
Guatemala (2015)	PSC	6–59	682	>8.3	3.5	Plasma	EIA, ELISA	BRINDA using CRP and AGP
Guatemala (2016)	PSC	6–59	569	>8.3	2.8	Plasma	EIA, ELISA	BRINDA using CRP and AGP
Guatemala (2018)	PSC	6–59	538	>8.3	13.3	Plasma	EIA, ELISA	BRINDA using CRP and AGP
Kyrgyzstan (2009)	PSC	6–59	1413	>8.3	33.6	Plasma	EIA, ELISA	Excluded samples with elevated CRP and/or AGP
Kyrgyzstan (2013)	PSC	6–29	2148	>8.3	37.4	Plasma	EIA, ELISA	BRINDA using AGP
Lao People's Democratic Republic (2006)	PSC	6–59	483	>4.4	44.7	Plasma	EIA, ELISA	Not specified
Malawi (2001)	PSC	6–36	365	>8.3	61.5	Serum	EIA, ELISA	Not specified
Malawi (2009)	PSC	6–59	980	>8.3	50.9	Serum	EIA, ELISA	Not specified
Malawi (2016)	PSC	6–59	1102	>8.3	55.2	Serum	EIA, ELISA	Not specified
Mexico (2006)	PSC	12–48	1707	>6.0	15.7	Serum	EIA, ELISA	Not specified
Mozambique (2002)	PSC	6–59	706	>8.5	36	Dried Blood Spot (Whole Blood)	EIA, ELISA	Not specified
Nepal (2016)	PSC	6–59	1651	>8.3	63.3	Plasma	EIA, ELISA	BRINDA using CRP and AGP
Papua New Guinea (2005)	PSC	6–59	872	>8.0	27.8	Dried Blood Spot (Whole Blood)	EIA, ELISA	Not specified
Tajikistan (2003)	PSC	6–59	1252	>8.5	38.8	Serum	EIA, ELISA	Not specified
Tajikistan (2009)	PSC	6–59	2104	≥3.3	8.6	Serum	EIA, ELISA	Not specified
Timor-Leste (2013)	PSC	6–59	547	>8.3	51.7	Serum	EIA, ELISA	Not specified
United Republic of Tanzania (2010)	PSC	6–59	6397	>8.3	35.3	Dried Blood Spot (Whole Blood)	EIA, ELISA	Not specified
Zimbabwe (2013)	PSC	6–59	1701	>8.3	72.2	Dried Blood Spot (Serum)	EIA, ELISA	Not specified

Abbreviations: AGP, α 1-acid-glycoprotein; CRP, C-reactive protein; EIA, ELISA; enzyme immunoassay, enzyme-linked immunosorbent assay; PSC, preschool-age children; sTfR, serum transferrin receptor; VMNIS, Vitamin and Mineral Nutrition Information System; WHO, World Health Organization

[1] Downloaded from VMNIS on March 29, 2022

[2] Reported from the 'Date' column of the VMNIS ranging from 2000–2019.
[3] Adjustment for inflammation summarized based on information under 'Indicator Comments' in the VMNIS database. Categorical adjustments refer to the method using internal correction factor(s) suggested by Thurnham et al. (2005; 2010) and BRINDA refers to adjustments using linear regression proposed by the Biomarkers Reflecting Inflammation and Nutritional Determinants of Anemia (BRINDA) project (Rohner et al., 2017).

Supplemental Table S2a. Overview of nationally representative surveys with data on iron deficiency based on serum or plasma ferritin among women of reproductive age implemented between 2000 to 2019 and reported in the WHO's micronutrients database VMNIS[1]

Survey (Year)[2]	Population	Age Range (yr)	*n*	Depleted Iron Stores Cut-Off (µg/L)	Depleted Iron Stores Prevalence (%)	Sample Type	Method of Analysis	Adjustment for Inflammation[3]
Afghanistan (2013)	WRA	15–49	Not Specified	<12	24	Serum	Not Specified	Categorical for elevated CRP and AGP
Argentina (2005)	WRA	10–49	5322	<15	18.7	Serum	EIA, ELISA	Not specified
Australia (2012)	WRA	16–44	1884	<15	12.1	Serum	ECLIA	Samples with CRP > 10 mg/L excluded
Austria (2012)	Women	18–64	204	<10	17.2	Plasma	EIA, ELISA	Not specified
Azerbaijan (2013)	NPW	15–49	2706	<15	34.1	Plasma	EIA, ELISA	Categorical for elevated CRP and AGP
Bahrain (2002)	WRA	14–49	384	<15	35.4	Serum	Not Specified	Not specified
Bangladesh (2011)	NPNLW	15–49	882	<15	7.1	Serum	EIA, ELISA	Categorical for elevated CRP and AGP
Belize (2011)	NPW	15–49	485	<15	17.8	Plasma	EIA, ELISA	Samples with AGP > 1 g/L excluded
Cambodia (2014)	WRA	15–49	738	<15	2.55	Plasma	EIA, ELISA	Categorical for elevated CRP and AGP
Cameroon (2009)	WRA	15–49	872	<15	15.3	Plasma	EIA, ELISA	Categorical for elevated CRP and AGP
Canada (2011)	WRA	20–49	Not Specified	<15	9.1	Serum	ECLIA	Not specified
Colombia (2005)	NPW	13–49	3483	<12	15.9	Plasma	ECLIA	Samples with CRP > 12 mg/L excluded
Colombia (2010)	WRA	13–49	9600	<12	17.1	Plasma	ECLIA	Samples with CRP > 12 mg/L excluded
Costa Rica (2009)	WRA	15–44	873	<12	8.7	Serum	EIA, ELISA	Not specified
Côte d'Ivoire (2007)	NPW	15–49	906	<15	16.7	Plasma	EIA, ELISA	Categorical for elevated CRP and AGP
Dominican Republic (2009)	NPW	15–49	541	<15	49.4	Plasma	EIA, ELISA	Samples with AGP > 1 g/L excluded
Ecuador (2012)	NPW	20–49	6957	<15	14.6	Serum	ECLIA	Not specified
Ethiopia (2005)	WRA	15–49	970	<15	29.4	Serum	EIA, ELISA	Unadjusted
Ethiopia (2015)	NPW	15–49	1700	<15	10	Serum	Not Specified	Categorical for elevated CRP and AGP
Fiji (2004)	NPW	15–44	738	<15	22.9	Serum	ECLIA	Unadjusted
Fiji (2010)	NPW	15–45	869	<15	7.9	Serum	ECLIA	Not specified

Survey (Year)[2]	Population	Age Range (yr)	*n*	Depleted Iron Stores Cut-Off (µg/L)	Depleted Iron Stores Prevalence (%)	Sample Type	Method of Analysis	Adjustment for Inflammation[3]
France (2007)	WRA	18–49	Not Specified	<15	13.5	Plasma	EIA, ELISA	Not specified
Gambia (2018)	NPW	15–49	1401	<15	41.4	Serum	EIA, ELISA	BRINDA using CRP and AGP
Georgia (2009)	NPW	15–49	Not Specified	<15	1.6	Serum	EIA, ELISA	Samples with CRP > 5 mg/L excluded
Ghana (2017)	NPW	15–49	987	<15	13.7	Serum	EIA, ELISA	Categorical for elevated CRP and AGP
Guatemala (2010)	NPW	15–49	1418	<15	11.2	Serum	EIA, ELISA	Unadjusted, also presented with ferritin cutoff > 30 µg/L when AGP > 1 g/L
Guatemala (2013)	NPW	15–49	1621	<15	9.9	Plasma	EIA, ELISA	Categorical for elevated CRP and AGP
Guatemala (2015)	NPW	15–49	1515	<15	14.7	Plasma	EIA, ELISA	BRINDA using CRP and AGP
Guatemala (2016)	NPW	15–49	1485	<15	13.8	Plasma	EIA, ELISA	BRINDA using CRP and AGP
Iraq (2012)	NPW	15–49	1152	<15	24.5	Serum	RIA	Not specified
Jordan (2002)	NPW	15–49	1303	<15	38.7	Serum	ECLIA	Not specified
Jordan (2010)	NPW	15–49	2035	<15	35.1	Serum	ECLIA	Unadjusted
Kenya (2011)	NPW	15–49	633	<15	21.3	Serum	Not Specified	Categorical for elevated CRP and AGP
Kyrgyzstan (2009)	NPW	17–	1026	<15	51	Plasma	EIA, ELISA	Excluded samples with elevated CRP and/or AGP
Lao People's Democratic Republic (2006)	NPW	15–49	818	<15	23.2	Plasma	EIA, ELISA	Categorical for elevated CRP and AGP
Lebanon (2003)	NPNLW	15–45	470	<15	27.2	Plasma	EIA, ELISA	Not specified
Liberia (2011)	NPW	15–49	1911	<15	19.6	Serum	EIA, ELISA	Categorical for elevated CRP and AGP
Malawi (2009)	NPW	15–49	431	<15	10.4	Serum	EIA, ELISA	Samples with CRP > 5 mg/L or AGP > 1 g/L excluded
Malawi (2016)	NPW	15–49	752	<15	15.1	Serum	EIA, ELISA	BRINDA using CRP and AGP

Supplemental Table S2a, continued

Survey (Year)[2]	Population	Age Range (yr)	*n*	Depleted Iron Stores Cut-Off (µg/L)	Depleted Iron Stores Prevalence (%)	Sample Type	Method of Analysis	Adjustment for Inflammation[3]
Maldives (2008)	WRA	15–49	1304	<20	38.4	Serum	EIA, ELISA	Unadjusted, also presented adjusted for CRP > 65 mg/L
Mexico (2006)	NPW	20–	3399	<12	18.1	Serum	EIA, ELISA	Not specified
Mexico (2012)	WRA	20–49	4136	<15	29.4	Serum	ECLIA	Categorical for elevated CRP
Mongolia (2004)	NPW	15–49	408	<12	13.7	Serum	Not Specified	Not specified
Mongolia (2010)	NPW	15–49	767	<12	28.2	Serum	EIA, ELISA	Samples with elevated CRP excluded
Morocco (2000)	NPW	15–49	445	<12	56.9	Serum	EIA, ELISA	Not specified
Nepal (2016)	NPW	15–49	2129	<15	18.7	Plasma	EIA, ELISA	BRINDA using CRP and AGP
New Zealand (2009)	Women	15–	1878	<12	8.4	Serum	ECLIA	Samples with CRP > 8 mg/L excluded
Nicaragua (2004)	NPW	15–49	310	<15	30.3	Serum	EIA, ELISA	Samples with AGP > 1 g/L excluded
Nicaragua (2005)	NPW	15–49	363	<15	31.2	Serum	EIA, ELISA	Samples with AGP > 1 g/L excluded
Nicaragua (2007)	WRA	15–49	Not Specified	<12	33	Serum	EIA, ELISA	Samples with AGP > 1 g/L excluded
Nigeria (2001)	WRA	Not Specified	3949	<12	12.7	Serum	RIA	Not specified
Oman (2004)	NPW	15–49	307	<15	33.9	Serum	ECLIA	Samples with CRP > 10 mg/L excluded
Pakistan (2001)	NPW	15–49	447	<12	48.3	Serum	ECLIA	Not specified
Republic of Korea (2010)	Pre-Menopausal Women	Not Specified	1640	Not Specified	Not Specified	Serum	RIA	Not specified
Senegal (2010)	NPW	15–49	983	<15	44.3	Plasma	EIA, ELISA	Categorical for elevated CRP and AGP
Sierra Leone (2013)	NPW	15–49	774	<15	8.3	Plasma	EIA, ELISA	Categorical for elevated CRP and AGP
South Africa (2005)	WRA	16–35	1906	<15	18.6	Serum	PENIA	Not specified
South Africa (2012)	WRA	16–35	1223	<15	15.3	Serum	EIA, ELISA	Not specified
Tajikistan (2003)	NPW	15–49	1195	<12	12.8	Serum	EIA, ELISA	Samples with CRP > 5 mg/L excluded
Tajikistan (2009)	NPW	15–49	2036	<12	9.3	Serum	EIA, ELISA	Samples with CRP > 5 mg/L excluded
Timor-Leste (2013)	NPW	14–60	592	<15	21.3	Serum	EIA, ELISA	Categorical for elevated CRP and AGP

Supplemental Table S2a, continued

Survey (Year)[2]	Population	Age Range (yr)	*n*	Depleted Iron Stores Cut-Off (µg/L)	Depleted Iron Stores Prevalence (%)	Sample Type	Method of Analysis	Adjustment for Inflammation[3]
United Kingdom of Great Britain and Northern (2001)	NPNLW	19–64	670	<15	11	Serum	EIA, ELISA	Not specified
United Kingdom of Great Britain and Northern (2010)	Women	19–64	348	<15	16	Plasma	Not Specified	Not specified
United Kingdom of Great Britain and Northern (2012)	Women	19–64	775	<15	15.5	Plasma	Not Specified	Not specified
United Kingdom of Great Britain and Northern (2014)	Women	19–64	323	<15	10	Plasma	Not Specified	Not specified
United Kingdom of Great Britain and Northern (2016)	Women	19–64	297	<15	12	Plasma	Not Specified	Not specified
United Kingdom of Great Britain and Northern (2019)	Women	19–64	427	<15	15	Plasma	Not Specified	Not specified
Uzbekistan (2008)	NPW	15–49	2582	<15	47.5	Serum	ITA	Categorical for elevated CRP
Uzbekistan (2017)	NPW	15–49	2077	<15	48.1	Serum	ECLIA	BRINDA using CRP and AGP
Viet Nam (2010)	NPW	15–49	1523	<15	13.7	Plasma	EIA, ELISA	Categorical for elevated CRP

Abbreviations: AGP, α 1-acid-glycoprotein; CRP, C-reactive protein; ECLIA, electrochemiluminescence immunoassay; EIA, ELISA; enzyme immunoassay, enzyme-linked immunosorbent assay; ITA, immunoturbidimetric assay; NPNLW, non-pregnant, non-lactating women; NPW, non-pregnant women; PENIA, particle-enhanced nephelometric immunoassay; RIA, radioimmunoassay; VMNIS, Vitamin and Mineral Nutrition Information System; WHO, World Health Organization; WRA, women of reproductive age

[1] Downloaded from VMNIS on March 29, 2022
[2] Reported from the 'Date' column of the VMNIS database, ranging from 2000–2019.
[3] Adjustment for inflammation summarized based on information under 'Indicator Comments' in the VMNIS database. Categorical adjustments refer to the method using internal correction factor(s) suggested by Thurnham et al. (2005; 2010) and BRINDA refers to adjustments using linear regression proposed by the Biomarkers Reflecting Inflammation and Nutritional Determinants of Anemia (BRINDA) project (Namaste et al., 2017).

Supplemental Table S2b. Overview of nationally representative surveys with data on iron deficiency based on soluble transferrin receptor among women of reproductive age implemented between 2000 to 2019 and reported in the WHO's micronutrients database (Ref VMNIS)[1]

Survey (Year)[2]	Population	Age Range (yr)	*n*	Inadequacy Threshold (mg/L)	Prevalence of Elevated sTfR (%)	Sample Type	Method of Analysis	Adjustment for Inflammation[3]
Australia (2012)	WRA	16–44	2099	>4.0	7.2	Serum	EIA, ELISA	Not specified
Cambodia (2014)	WRA	15–49	738	>8.3	33.89	Plasma	EIA, ELISA	Not specified
Cameroon (2009)	WRA	15–49	872	>8.3	31.9	Plasma	EIA, ELISA	Unadjusted
Côte d'Ivoire (2007)	NPW	15–49	907	>8.3	6.7	Plasma	EIA, ELISA	Not specified
Ethiopia (2015)	NPW	15–49	1726	>4.4	16.4	Serum	EIA, ELISA	Not specified
Guatemala (2016)	NPW	15–49	1484	>8.3	1.6	Plasma	EIA, ELISA	BRINDA using CRP and AGP
Kyrgyzstan (2009)	NPW	17–	1026	>8.3	23.9	Plasma	EIA, ELISA	Samples with elevated CRP or AGP excluded
Lao People's Democratic Republic (2006)	NPW	15–49	818	>4.4	28	Plasma	EIA, ELISA	Not specified
Malawi (2001)	NPW	15–45	346	>8.3	32.4	Serum	EIA, ELISA	Not specified
Malawi (2009)	NPW	15–49	509	>8.3	26.1	Serum	EIA, ELISA	Not specified
Malawi (2016)	NPW	15–49	752	>8.3	24.6	Serum	EIA, ELISA	Not specified
Mexico (2006)	NPW	20–	3390	>6.0	9.5	Serum	EIA, ELISA	Not specified
Mozambique (2002)	NPW	Not Specified	557	>8.5	12.7	Dried Blood Spot (Whole Blood)	EIA, ELISA	Not specified
Nepal (2016)	NPW	15–49	2129	>8.3	13.4	Plasma	EIA, ELISA	BRINDA using CRP and AGP
Papua New Guinea (2005)	NPW	15–49	753	>8.0	19.5	Dried Blood Spot (Whole Blood)	EIA, ELISA	Not specified
Tajikistan (2003)	NPW	15–49	1415	>8.5	29.2	Serum	EIA, ELISA	Not specified
Tajikistan (2009)	NPW	15–49	2073	≥3.3	4.8	Serum	EIA, ELISA	Not specified
Timor-Leste (2013)	NPW	14–60	592	>8.3	29.4	Serum	EIA, ELISA	Not specified
United Kingdom of Great Britain and Northern (2012)	Women	19–64	761	Not Specified	Not Specified	Plasma	EIA, ELISA	Not specified
United Republic of Tanzania (2010)	NPNLW	15–49	5871	>8.3	28.9	Dried Blood Spot (Whole Blood)	EIA, ELISA	Not specified
Zimbabwe (2013)	WRA	15–49	891	>8.3	61.8	Dried Blood Spot (Serum)	EIA, ELISA	Not specified

Abbreviations: AGP, α 1-acid-glycoprotein; CRP, C-reactive protein; EIA, ELISA; enzyme immunoassay, enzyme-linked immunosorbent assay; NPNLW, non-pregnant, non-lactating women; NPW, non-pregnant women; sTfR, serum transferrin receptor; VMNIS, Vitamin and Mineral Nutrition Information System; WHO, World Health Organization; WRA, women of reproductive age

[1] Downloaded from VMNIS on March 29, 2022
[2] Reported from the 'Date' column of the VMNIS ranging from 2000–2019.
[3] Adjustment for inflammation summarized based on information under 'Indicator Comments' in the VMNIS database. Categorical adjustments refer to the method using internal correction factor(s)

suggested by Thurnham et al. (2005; 2010) and BRINDA refers to adjustments using linear regression proposed by the Biomarkers Reflecting Inflammation and Nutritional Determinants of Anemia (BRINDA) project (Rohner et al., 2017).

Supplemental Table S3a. Overview of nationally representative surveys with data on vitamin A deficiency based on serum or plasma retinol among young children implemented between 2000 to 2019 and reported in the WHO's micronutrients database VMNIS[1]

Survey (Year)[2]	Population	Age Range (mo)	*n*	Vitamin A Deficiency Cut-Off (µmol/L)	Prevalence of Vitamin A Deficiency (%)	Sample Type	Method of Analysis	Adjustment for Inflammation[3]
Afghanistan (2013)	PSC	6–59	Not Specified	<0.70	50	Serum	HPLC	Categorical for elevated CRP and AGP
Argentina (2005)	PSC	24–60	Not Specified	<0.70	14	Serum	HPLC	Not specified
Bangladesh (2011)	PSC	6–59	873	<0.70	21	Serum	HPLC	Categorical for elevated CRP and AGP
Belize (2011)	PSC	6–59	971	<0.70	1	Plasma	HPLC	Unadjusted
Brazil (2006)	PSC	6–59	3499	<0.70	17	Dried Blood Spot (Whole Blood)	HPLC	Not specified
Burundi (2005)	PSC	6–59	587	<0.70	28	Dried Blood Spot (Serum)	HPLC	Samples with AGP > 1 g/L excluded
Cameroon (2000)	PSC	12–71	2375	≤0.70	39	Serum	HPLC	Not specified
China (2000)	PSC	0–72	7826	<0.70	12	Serum	FL	Not specified
Colombia (2005)	PSC	12–48	4409	<0.70	6	Plasma	HPLC	Not specified
Colombia (2010)	PSC	12–48	4394	<0.70	24	Serum	HPLC	Not specified
Costa Rica (2009)	PSC	12–72	377	<0.70	3	Serum	HPLC	Not specified
Dominican Republic (2009)	PSC	6–59	330	<0.70	12	Plasma	HPLC	Samples with AGP > 1 g/L excluded
Ecuador (2012)	PSC	6–59	2044	<0.70	18	Serum	HPLC	Unadjusted, also presented w/o inflammation
Ethiopia (2006)	PSC	6–71	996	<0.70	38	Serum	HPLC	Not specified
Ethiopia (2015)	PSC	6–59	1148	<0.70	14	Serum	HPLC	Categorical for elevated CRP and AGP
Guatemala (2010)	PSC	6–59	1198	≤0.70	0	Serum	HPLC	Unadjusted, also presented w/o inflammation
Guyana (2012)	PSC	–59	212	<0.70	11	Serum	LC-MS	Not specified
Haiti (2005)	PSC	6–59	780	≤0.70	32	Dried Blood Spot (Whole Blood)	HPLC	Not specified
India (2018)	PSC	12–48	6694	<0.70	18	Serum	HPLC	Samples with CRP > 5 mg/L excluded
Iran (Islamic Republic of) (2018)	PSC	10–36	4261	Not Specified	Not Specified	Serum	HPLC	Not specified
Iraq (2012)	PSC	12–59	2024	<0.70	15	Serum	HPLC	Not specified
Jordan (2002)	PSC	12–59	1036	<0.70	15	Serum	HPLC	Not specified
Jordan (2010)	PSC	12–59	915	<0.70	18	Serum	LC-MS	Unadjusted

Supplemental Table S3a, continued

Survey (Year)[2]	Population	Age Range (mo)	*n*	Vitamin A Deficiency Cut-Off (µmol/L)	Prevalence of Vitamin A Deficiency (%)	Sample Type	Method of Analysis	Adjustment for Inflammation[3]
Lao People's Democratic Republic (2000)	PSC	6–59	419	<0.70	45	Dried Blood Spot (Serum)	HPLC	Not specified
Madagascar (2000)	PSC	6–59	589	<0.70	42	Serum	HPLC	Not specified
Malawi (2001)	PSC	6–36	476	<0.70	59	Serum	HPLC	Not specified
Maldives (2008)	PSC	0–60	1247	<0.70	5	Serum	HPLC	Unadjusted
Mexico (2012)	PSC	12–59	2593	<0.70	16	Serum	HPLC	Unadjusted
Mozambique (2002)	PSC	6–59	705	<0.70	69	Dried Blood Spot (Whole Blood)	HPLC	Not specified
Nepal (2016)	PSC	6–59	657	<0.70	11	Serum	HPLC	BRINDA using CRP and AGP
Nicaragua (2000)	PSC	6–59	2381	<0.70	9	Plasma	HPLC	Unadjusted, also presented w/o inflammation
Nicaragua (2003)	PSC	6–59	470	<0.70	0	Plasma	HPLC	Not specified
Nicaragua (2004)	PSC	6–59	344	<0.70	1	Plasma	HPLC	Samples with AGP > 1 g/L excluded
Nicaragua (2005)	PSC	6–59	365	<0.70	2	Plasma	HPLC	Samples with AGP > 1 g/L excluded
Nicaragua (2007)	PSC	6–59	Not Specified	<0.70	10	Plasma	HPLC	Not specified
Nigeria (2001)	PSC	0–59	3099	<0.70	30	Serum	HPLC	Not specified
Oman (2004)	PSC	6–59	152	<0.70	6	Serum	HPLC	Samples with CRP >10 mg/L excluded
Pakistan (2001)	PSC	6–59	5682	<0.70	13	Serum	HPLC	Not specified
Peru (2000)	PSC	0–48	657	<0.70	13	Plasma	HPLC	Not specified
Peru (2001)	PSC	0–48	734	<0.70	15	Plasma	HPLC	Not specified
Philippines (2003)	PSC	6–59	3544	<0.70	40	Plasma	HPLC	Not specified
Philippines (2008)	PSC	0–60	2408	<0.70	15	Serum	HPLC	Not specified
Philippines (2014)	PSC	6–71	3139	<0.70	20	Serum	HPLC	Not specified
Senegal (2010)	PSC	12–59	1418	<0.70	18	Plasma	HPLC	Categorical for elevated CRP and AGP
South Africa (2005)	PSC	12–71	833	<0.70	65	Serum	HPLC	Categorical for elevated CRP
South Africa (2012)	PSC	0–59	438	<0.70	44	Serum	HPLC	Unadjusted
Sri Lanka (2006)	PSC	6–60	768	<0.70	29	Serum	HPLC	Not specified
Timor-Leste (2013)	PSC	6–59	546	<0.70	8	Serum	HPLC	Categorical for elevated CRP and AGP
Uganda (2001)	PSC	6–59	859	<0.70	28	Dried Blood Spot (Whole Blood)	HPLC	Not specified

Supplemental Table S3a, continued

Survey (Year)[2]	Population	Age Range (mo)	*n*	Vitamin A Deficiency Cut-Off (µmol/L)	Prevalence of Vitamin A Deficiency (%)	Sample Type	Method of Analysis	Adjustment for Inflammation[3]
United Kingdom of Great Britain and Northern (2012)	PSC	12–36	33	≤0.70	0	Plasma	HPLC	Not specified
Uzbekistan (2017)	PSC	6–59	1665	<0.70	6	Serum	HPLC	BRINDA for CRP and AGP
Viet Nam (2010)	PSC	10–75	546	<0.70	10	Plasma	HPLC	Categorical for elevated CRP
Zambia (2003)	PSC	6–59	659	≤0.70	54	Plasma	HPLC	Unadjusted

Abbreviations: AGP, α 1-acid-glycoprotein; CRP, C-reactive protein; FL, fluorometric; HPLC, high performance liquid chromatography; LC-MS, liquid chromatography–mass spectrometry; PSC, preschool-age children; VMNIS, Vitamin and Mineral Nutrition Information System; WHO, World Health Organization

[1] Downloaded from VMNIS on March 29, 2022
[2] Reported from the 'Date' column of the VMNIS ranging from 2000–2019.
[3] Adjustment for inflammation summarized based on information under 'Indicator Comments' in the VMNIS database. Categorical adjustments refer to the method using internal correction factor(s) suggested by Thurnham et al. (2005; 2010) and BRINDA refers to adjustments using linear regression proposed by the Biomarkers Reflecting Inflammation and Nutritional Determinants of Anemia (BRINDA) project (Larson et al., 2017; Namaste et al, 2020).

Supplemental Table 3b. Overview of nationally representative surveys with data on vitamin A deficiency based on serum or plasma retinol binding protein among young children implemented between 2000 to 2019 and reported in the WHO's micronutrients database VMNIS[1]

Survey (Year)[2]	Population	Age Range (mo)	*n*	Inadequacy Cut-Off (µmol/L)	Inadequacy Prevalence (%)	Sample Type	Method of Analysis	Adjustment for Inflammation[3]
Azerbaijan (2013)	PSC	6–59	1075	<0.70	8	Plasma	EIA, ELISA	Categorical for elevated CRP and AGP
Cambodia (2014)	PSC	6–71	775	<0.70	9	Plasma	EIA, ELISA	Not specified
Cameroon (2009)	PSC	12–59	838	<0.83	35	Plasma	EIA, ELISA	Categorical for elevated CRP and AGP
Côte d'Ivoire (2007)	PSC	6–59	782	<0.70	24	Plasma	EIA, ELISA	Categorical for elevated CRP and AGP
Gambia (2018)	PSC	6–59	1012	<0.70	18	Serum	EIA, ELISA	BRINDA for CRP and AGP
Ghana (2017)	PSC	6–59	1165	<0.70	21	Serum	EIA, ELISA	Categorical for elevated CRP and AGP
Guatemala (2013)	PSC	6–59	858	<0.70	3	Plasma	EIA, ELISA	Categorical for elevated CRP and AGP
Guatemala (2015)	PSC	6–59	682	<0.63	1	Plasma	EIA, ELISA	BRINDA for CRP and AGP
Kenya (2011)	PSC	6–59	918	<0.70	9	Serum	EIA, ELISA	Categorical for elevated CRP and AGP
Kyrgyzstan (2009)	PSC	6–59	1413	<0.71	2	Plasma	EIA, ELISA	Samples with elevated CRP and/or AGP excluded
Kyrgyzstan (2013)	PSC	6–29	2148	<0.71	4	Plasma	EIA, ELISA	BRINDA for CRP and AGP
Liberia (2011)	PSC	6–35	1416	<0.70	13	Serum	Not Specified	Categorical for elevated CRP and AGP
Malawi (2009)	PSC	6–59	981	<0.78	22	Serum	EIA, ELISA	Unadjusted
Malawi (2016)	PSC	6–59	1102	<0.46	4	Serum	EIA, ELISA	Unadjusted
Mongolia (2010)	PSC	6–59	433	<0.70	32	Serum	EIA, ELISA	Samples with elevated CRP excluded
Nepal (2016)	PSC	6–59	1651	<0.69	3	Plasma	EIA, ELISA	BRINDA for CRP and AGP

Supplemental Table S3b, continued

Survey (Year)[2]	Population	Age Range (mo)	*n*	Inadequacy Cut-Off (µmol/L)	Inadequacy Prevalence (%)	Sample Type	Method of Analysis	Adjustment for Inflammation[3]
Papua New Guinea (2005)	PSC	6–59	875	<0.70	26	Dried Blood Spot (Whole Blood)	EIA, ELISA	Unadjusted, also presented w/o inflammation
Sierra Leone (2013)	PSC	6–59	654	<0.70	17	Plasma	EIA, ELISA	Categorical for elevated CRP and AGP
Timor-Leste (2013)	PSC	6–59	547	<0.70	10	Serum	EIA, ELISA	Categorical for elevated CRP and AGP
Uganda (2006)	PSC	6–59	2460	<0.825	20	Dried Blood Spot (Whole Blood)	EIA, ELISA	Not specified
Uganda (2011)	PSC	6–59	2091	<0.825	38	Dried Blood Spot (Whole Blood)	EIA, ELISA	Unadjusted, also presented categorical for elevated CRP
Uganda (2016)	PSC	6–59	4694	<0.825	9	Dried Blood Spot (Whole Blood)	EIA, ELISA	Categorical for elevated CRP
United Republic of Tanzania (2010)	PSC	6–59	6294	<0.825	38	Dried Blood Spot (Whole Blood)	EIA, ELISA	Unadjusted, also presented categorical for elevated CRP
Zimbabwe (2013)	PSC	6–59	1727	<0.825	21	Dried Blood Spot (Whole Blood)	EIA, ELISA	Categorical for elevated CRP

Abbreviations: AGP, α 1-acid-glycoprotein; CRP, C-reactive protein; EIA, ELISA; enzyme immunoassay, enzyme-linked immunosorbent assay; PSC, preschool-age children; RBP, retinol binding protein; VMNIS, Vitamin and Mineral Nutrition Information System; WHO, World Health Organization

[1] Downloaded from VMNIS on March 29, 2022
[2] Reported from the 'Date' column of the VMNIS ranging from 2000–2019.
[3] Adjustment for inflammation summarized based on information under 'Indicator Comments' in the VMNIS database. Categorical adjustments refer to the method using internal correction factor(s) suggested by Thurnham et al. (2005; 2010) and BRINDA refers to adjustments using linear regression proposed by the Biomarkers Reflecting Inflammation and Nutritional Determinants of Anemia (BRINDA) project (Larson et al., 2017; Namaste et al, 2020).

Supplemental Table S4a. Overview of nationally representative surveys with data on vitamin A deficiency based on serum or plasma retinol among women of reproductive age implemented between 2000 to 2019 and reported in the WHO's micronutrients database VMNIS[1]

Survey (Year)[2]	Population	Age Range (yr)	*n*	Vitamin A Deficiency Cut-Off (µmol/L)	Prevalence of Vitamin A Deficiency (%)	Sample Type	Method of Analysis	Adjustment for Inflammation[3]
Afghanistan (2013)	WRA	15–49	Not Specified	<0.70	11	Serum	HPLC	Categorical for elevated CRP and AGP
Austria (2012)	Women	18–64	210	Not Specified	Not Specified	Plasma	HPLC	Not specified
Bangladesh (2011)	NPNLW	15–49	918	<0.70	5	Serum	HPLC	Categorical for elevated CRP and AGP
Belize (2011)	NPW	15–49	937	<0.70	1	Plasma	HPLC	Unadjusted
Brazil (2006)	NPW	15–49	5698	<0.70	12	Dried Blood Spot (Whole Blood)	HPLC	Not specified
Ethiopia (2015)	NPW	15–49	1619	<0.70	3	Serum	HPLC	Categorical for elevated CRP and AGP
Fiji (2004)	NPW	15–44	731	<0.70	3	Serum	HPLC	Not specified
Iraq (2012)	NPW	15–49	1066	<0.70	4	Serum	HPLC	Not specified
Jordan (2010)	NPW	15–49	2032	<0.70	5	Serum	LC-MS	Unadjusted
Lao People's Democratic Republic (2000)	WRA	19–25	172	<0.70	22	Dried Blood Spot (Serum)	HPLC	Not specified
Madagascar (2000)	WRA	15–49	296	Not Specified	Not Specified	Serum	HPLC	Not specified
Malawi (2001)	WRA	15–45	464	<0.70	57	Serum	HPLC	Not specified
Maldives (2008)	WRA	15–49	1297	<0.70	5	Serum	HPLC	Unadjusted
Mozambique (2002)	NPW	Not Specified	557	<0.70	11	Dried Blood Spot (Whole Blood)	HPLC	Not specified
Nepal (2016)	NPW	15–49	527	<0.70	5	Serum	HPLC	BRINDA for CRP and AGP
Nicaragua (2000)	WRA	Not Specified	2221	<0.70	1	Plasma	HPLC	Unadjusted
Nigeria (2001)	WRA	Not Specified	3148	<0.70	4	Serum	HPLC	Unadjusted
Oman (2004)	NPW	15–49	341	<0.70	0	Serum	HPLC	Samples with CRP > 10 mg/L excluded
Pakistan (2001)	NPW	15–49	484	<0.70	5	Serum	HPLC	Not specified
Peru (2000)	WRA	15–49	968	<0.70	10	Plasma	HPLC	Not specified
Peru (2001)	NPW	15–49	1228	<0.70	9	Plasma	HPLC	Not specified
Senegal (2010)	NPW	15–49	983	<0.70	2	Plasma	HPLC	Categorical for elevated CRP and AGP
South Africa (2005)	WRA	16–35	1834	<0.70	27	Serum	HPLC	Categorical for elevated CRP
South Africa (2012)	WRA	16–35	1158	<0.70	13	Serum	HPLC	Unadjusted

Supplemental Table S4a, continued

Survey (Year)[2]	Population	Age Range (yr)	*n*	Vitamin A Deficiency Cut-Off (µmol/L)	Prevalence of Vitamin A Deficiency (%)	Sample Type	Method of Analysis	Adjustment for Inflammation[3]
Sri Lanka (2006)	NPW	–49	290	<0.70	15	Serum	HPLC	Not specified
Timor-Leste (2013)	NPW	14–60	616	<0.70	3	Serum	HPLC	Categorical for elevated CRP and AGP
Uganda (2001)	NPNLW	15–49	461	<0.70	22	Dried Blood Spot (Whole Blood)	HPLC	Not specified
United Kingdom of Great Britain and Northern (2001)	NPNLW	19–64	616	<0.70	0	Plasma	HPLC	Not specified
United Kingdom of Great Britain and Northern (2012)	Women	19–64	756	≤0.70	1	Plasma	HPLC	Not specified
Uzbekistan (2017)	NPW	15–49	1990	<0.70	3	Serum	HPLC	BRINDA for CRP and AGP
Viet Nam (2010)	NPW	15–49	1475	<0.70	2	Plasma	HPLC	Categorical for elevated CRP
Zambia (2003)	NPW	15–49	Not Specified	≤0.70	13	Plasma	HPLC	Unadjusted, also presented categorical for elevated CRP and AGP

Abbreviations: AGP, α 1-acid-glycoprotein; CRP, C-reactive protein; HPLC, high performance liquid chromatography; LC-MS, liquid chromatography–mass spectrometry; NPNLW, non-pregnant, non-lactating women; NPW, non-pregnant women; VMNIS, Vitamin and Mineral Nutrition Information System; WHO, World Health Organization; WRA, women of reproductive age

[1] Downloaded from VMNIS on March 29, 2022
[2] Reported from the 'Date' column of the VMNIS ranging from 2000–2019.
[3] Adjustment for inflammation summarized based on information under 'Indicator Comments' in the VMNIS database. Categorical adjustments refer to the method using internal correction factor(s) suggested by Thurnham et al. (2005; 2010) and BRINDA refers to adjustments using linear regression proposed by the Biomarkers Reflecting Inflammation and Nutritional Determinants of Anemia (BRINDA) project (Larson et al., 2017; Namaste et al, 2020).

Supplemental Table S4b. Overview of nationally representative surveys with data on vitamin A deficiency based on serum or plasma retinol binding protein among women of reproductive age implemented between 2000 to 2019 and reported in the WHO's micronutrients database VMNIS[1]

Survey (Year)[2]	Population	Age Range (yr)	*n*	Inadequacy Cut-Off (µmol/L)	Prevalence of Vitamin A Deficiency (%)	Sample Type	Method of Analysis	Adjustment for Inflammation[3]
Austria (2012)	Women	18–64	210	Not Specified	Not Specified	Plasma	EIA, ELISA	Not specified
Azerbaijan (2013)	NPW	15–49	Not Specified	<0.70	1	Plasma	EIA, ELISA	Categorical for elevated CRP and AGP
Cambodia (2014)	WRA	15–49	739	<0.70	3	Plasma	EIA, ELISA	Not specified
Cameroon (2009)	WRA	15–49	872	<0.78	3	Plasma	EIA, ELISA	Categorical for elevated CRP and AGP
Côte d'Ivoire (2007)	NPW	15–49	907	<0.70	1	Plasma	EIA, ELISA	Categorical for elevated CRP and AGP
Gambia (2018)	NPW	15–49	1391	<0.70	2	Serum	EIA, ELISA	Unadjusted
Ghana (2017)	NPW	15–49	987	<0.70	2	Serum	EIA, ELISA	Unadjusted
Guatemala (2013)	NPW	15–49	1621	<0.70	0	Plasma	EIA, ELISA	Categorical for elevated CRP and AGP
Guatemala (2015)	NPW	15–49	1515	<0.63	0	Plasma	EIA, ELISA	Unadjusted
Kenya (2011)	NPW	15–49	632	<0.70	1	Serum	EIA, ELISA	Categorical for elevated CRP and AGP
Kyrgyzstan (2009)	NPW	17–	1026	<0.71	1	Plasma	EIA, ELISA	Samples with elevated CRP and/or AGP excluded
Liberia (2011)	NPW	15–49	1911	<0.70	2	Serum	Not Specified	Categorical for elevated CRP and AGP
Malawi (2009)	NPW	15–49	509	<0.78	2	Serum	EIA, ELISA	Unadjusted
Malawi (2016)	NPW	15–49	752	<0.46	0	Serum	EIA, ELISA	Unadjusted
Nepal (2016)	NPW	15–49	2129	<0.64	1	Plasma	EIA, ELISA	BRINDA for CRP and AGP
Sierra Leone (2013)	NPW	15–49	817	<0.70	2	Plasma	EIA, ELISA	Categorical for elevated CRP and AGP
Timor-Leste (2013)	NPW	14–60	592	<1.05	5	Serum	EIA, ELISA	Categorical for elevated CRP and AGP
Uganda (2006)	NPNLW	15–49	1542	<1.24	21	Dried Blood Spot (Whole Blood)	EIA, ELISA	Not specified

Supplemental Table S4b, continued

Survey (Year)[2]	Population	Age Range (yr)	*n*	Inadequacy Cut-Off (µmol/L)	Prevalence of Vitamin A Deficiency (%)	Sample Type	Method of Analysis	Adjustment for Inflammation[3]
Uganda (2011)	NPNLW	15–49	1547	<1.24	37	Dried Blood Spot (Whole Blood)	EIA, ELISA	Unadjusted, also presented categorical for elevated CRP
United Republic of Tanzania (2010)	NPNLW	15–49	5707	<1.24	42	Dried Blood Spot (Whole Blood)	EIA, ELISA	Unadjusted, also presented categorical for elevated CRP
Zimbabwe (2013)	NPW	15–49	838	<1.24	23	Dried Blood Spot (Whole Blood)	EIA, ELISA	Categorical for elevated CRP

Abbreviations: AGP, α 1-acid-glycoprotein; CRP, C-reactive protein; EIA, ELISA; enzyme immunoassay, enzyme-linked immunosorbent assay; NPNLW, non-pregnant, non-lactating women; NPW, non-pregnant women; VMNIS, Vitamin and Mineral Nutrition Information System; WHO, World Health Organization; WRA, women of reproductive age

[1] Downloaded from VMNIS on March 29, 2022
[2] Reported from the 'Date' column of the VMNIS ranging from 2000–2019.
[3] Adjustment for inflammation summarized based on information under 'Indicator Comments' in the VMNIS database. Categorical adjustments refer to the method using internal correction factor(s) suggested by Thurnham et al. (2005; 2010) and BRINDA refers to adjustments using linear regression proposed by the Biomarkers Reflecting Inflammation and Nutritional Determinants of Anemia (BRINDA) project (Larson et al., 2017; Namaste et al, 2020).

Supplemental Table S5. Overview of nationally representative surveys with data on zinc deficiency based on serum or plasma zinc among young children implemented between 2000 to 2019 and reported in the WHO's micronutrients database VMNIS[1]

Survey (Year)[2]	Population	Age Range (mo)	*n*	Deficiency Cut-Off (µmol/L)	Prevalence of Zinc Deficiency (%)	Sample Type	Fasting	Method of Analysis	Adjustment for Inflammation[3]
Afghanistan (2013)	PSC	6–59	728	<9.2	15.1	Serum	Not Specified	AAS	Categorical for elevated CRP and AGP
Azerbaijan (2013)	PSC	6–59	1040	<9.9	10.7	Plasma	Casual	ICP-OES	Unadjusted
Bangladesh (2011)	PSC	6–59	662	<9.9	44.6	Serum	Casual	AAS	Categorical for elevated CRP and AGP
Cambodia (2014)	PSC	6–72	656	<9.9	67.5	Plasma	Not Specified	AAS	Not specified
Cameroon (2009)	PSC	12–59	680	<9.9	82.6	Plasma	Casual	ICP-AES	Categorical for elevated CRP and AGP
Colombia (2005)	PSC	12–48	3974	<9.9	26.9	Serum	Casual	AAS	Not specified
Colombia (2010)	PSC	12–48	4279	<9.9	43.3	Serum	Casual	AAS	Not specified
Costa Rica (2009)	PSC	12–72	224	<10.7	23.9	Serum	Casual	AAS	Not specified
Ecuador (2012)	PSC	6–59	2045	<9.9	27.5	Serum	Fasting/First morning sample	AAS	Unadjusted, also presented w/o inflammation
Ethiopia (2015)	PSC	6–59	1143	<10.7	35	Serum	Casual	AAS	Not specified
Guatemala (2010)	PSC	6–59	1196	<10.7	34.9	Serum	Casual	AAS	Not specified
Guatemala (2013)	PSC	6–59	65	<9.9	40	Serum	Not Specified	AAS	Not specified
Guatemala (2016)	PSC	6–59	63	<9.9	13.3	Plasma	Not Specified	Not Specified	Not specified
India (2018)	PSC	12–48	8662	<9.9	18.9	Serum	Fasting/First morning sample	AAS	Samples with CRP > 5 mg/L excluded
Iran (Islamic Republic of) (2018)	PSC	10–36	4261	Not Specified	Not Specified	Serum	Not Specified	AAS	Not specified
Kenya (2011)	PSC	6–59	768	<9.9	83.3	Plasma	Not Specified	Not Specified	Categorical for elevated CRP and AGP
Malawi (2016)	PSC	6–59	1086	<9.9	60.4	Serum	Casual	AAS	Unadjusted
Maldives (2008)	PSC	0–60	1255	<9.2	16	Plasma	Not Specified	AAS	Not specified

Supplemental Table S5, continued

Survey (Year)[2]	Population	Age Range (mo)	*n*	Deficiency Cut-Off (μmol/L)	Prevalence of Zinc Deficiency (%)	Sample Type	Fasting	Method of Analysis	Adjustment for Inflammation[3]
Mexico (2006)	PSC	12–48	1550	<9.9	28.1	Serum	Fasting/First morning sample	ICP-AES	Not specified
Nepal (2016)	PSC	6–59	1647	<9.9	20.7	Serum	Casual	AAS	BRINDA using CRP and AGP
Nigeria (2001)	PSC	0–59	2725	<12.2	20	Serum	Not Specified	Not Specified	Not specified
Pakistan (2001)	PSC	6–59	5644	<9.1	37.1	Serum	Not Specified	AAS	Not specified
Philippines (2008)	PSC	6–59	2370	<9.9	21.6	Serum	Casual	AAS	Not specified
Philippines (2014)	PSC	6–59	3124	<9.9	17.9	Serum	Not Specified	AAS	Not specified
Senegal (2010)	PSC	12–59	1151	<9.9	40.7	Plasma	Not Specified	AAS	Categorical for elevated CRP and AGP
South Africa (2005)	PSC	12–36	154	<9.9	51.3	Serum	Not Specified	AAS	Not specified
Sri Lanka (2012)	PSC	6–59	4463	<9.9	5.1	Serum	Not Specified	AAS	Samples with CRP > 5 g/L excluded
Timor-Leste (2013)	PSC	6–59	580	<9.9	60.3	Serum	Not Specified	AAS	Not specified
Viet Nam (2010)	PSC	10–75	563	<9.9	51.9	Serum	Casual	AAS	Unadjusted

Abbreviations: AAS, atomic absorption spectroscopy; AGP, α 1-acid-glycoprotein; CRP, C-reactive protein; ICP-AES, inductively coupled plasma atomic emission spectroscopy; ICP-MS, inductively coupled plasma mass spectrometer; ICP-OES, inductively coupled plasma optical emission spectrometry; PSC, preschool-age children; VMNIS, Vitamin and Mineral Nutrition Information System; WHO, World Health Organization

[1] Downloaded from VMNIS on March 29, 2022
[2] Reported from the 'Date' column of the VMNIS ranging from 2000–2019.
[3] Adjustment for inflammation summarized based on information under 'Indicator Comments' in the VMNIS database. Categorical adjustments refer to the method using internal correction factor(s) suggested by Thurnham et al. (2005; 2010) and BRINDA refers to adjustments using linear regression proposed by the Biomarkers Reflecting Inflammation and Nutritional Determinants of Anemia (BRINDA) project (McDonald et al., 2020).

Supplemental Table S6. Overview of nationally representative surveys with data on zinc deficiency based on serum or plasma zinc among women of reproductive age implemented between 2000 to 2019 and reported in the WHO's micronutrients database VMNIS[1]

Survey (Year)[2]	Population	Age Range (yr)	*n*	Deficiency Cut-Off (µmol/L)	Prevalence of Zinc Deficiency (%)	Sample Type	Fasting	Method of Analysis	Adjustment for Inflammation [3]
Afghanistan (2013)	WRA	15–49	1187	<9.2	23.4	Serum	Not Specified	AAS	Categorical for elevated CRP and AGP
Austria (2012)	Women	18–64	229	<10.7	18.1	Plasma	Fasting/First Morning Sample	AAS	Not specified
Bangladesh (2011)	NPNLW	15–49	1073	<10.1	57.3	Serum	Casual	AAS	Categorical for elevated CRP and AGP
Cambodia (2014)	WRA	15–49	720	<9.9	62.8	Plasma	Not Specified	AAS	Not specified
Cameroon (2009)	NPNLW	15–49	446	<10.1	83.2	Plasma	Casual	ICP-AES	Categorical for elevated CRP and AGP
Ecuador (2012)	NPW	12–49	8186	<10.7	56.1	Serum	Fasting/First Morning Sample	AAS	Unadjusted, also presented w/o inflammation
Ethiopia (2015)	NPW	15–49	1625	<10.7	33.8	Serum	Casual	AAS	Not specified
Fiji (2004)	NPW	15–44	717	<10.1	39.2	Serum	Casual	AAS	Not specified
Fiji (2010)	NPW	15–45	869	<10.1	0	Serum	Not Specified	AAS	Not specified
Guatemala (2013)	NPW	15–49	85	<10.1	34.1	Serum	Not Specified	AAS	Not specified
Guatemala (2016)	NPW	15–49	130	<10.1	18.3	Plasma	Not Specified	Not Specified	Not specified
Kenya (2011)	NPW	15–49	653	<10.1	82.3	Plasma	Not Specified	Not Specified	Categorical for elevated CRP and AGP
Kuwait (2003)	NPW	15–80	322	Not Specified	Not Specified	Serum	Fasting/First Morning Sample	AAS	Not specified
Malawi (2016)	NPW	15–49	757	<10.7	62.5	Serum	Casual	AAS	Unadjusted
Maldives (2008)	WRA	15–49	1282	<9.2	26.8	Plasma	Not Specified	AAS	Not specified
Mexico (2006)	NPW	30-39	601	<9.9	30.6	Serum	Fasting/First morning sample	ICP-AES	Not specified
Nepal (2016)	NPW	15–49	2132	<10.1	24.3	Serum	Casual	AAS	Unadjusted
Nigeria (2001)	WRA	Not Specified	3779	<12.2	28.1	Serum	Not Specified	Not Specified	Not specified
Pakistan (2001)	NPW	15–49	451	<9.1	40.1	Serum	Not Specified	AAS	Not specified
Philippines (2008)	Women	20–59	2892	<10.7	31.2	Serum	Casual	AAS	Not specified
Philippines (2014)	Women	20–59	7139	<10.1	28.4	Serum	Not Specified	AAS	Not specified
Senegal (2010)	NPW	15–49	983	<10.1	67.2	Plasma	Not Specified	AAS	Unadjusted
United Kingdom of Great Britain and Northern (2012)	Women	19–64	745	Not Specified	Not Specified	Plasma	Fasting/First morning sample	ICP-MS	Not specified
Viet Nam (2010)	NPW	15–49	1522	<10.1	67.2	Serum	Casual	AAS	Unadjusted

Abbreviations: AAS, atomic absorption spectroscopy; AGP, α 1-acid-glycoprotein; CRP, C-reactive protein; ICP-AES, inductively coupled plasma atomic emission spectroscopy; ICP-MS, inductively coupled plasma mass spectrometer; NPNLW, non-pregnant, non-lactating women; NPW, non-pregnant women; VMNIS, Vitamin and Mineral Nutrition Information System; WHO, World Health Organization; WRA, women of reproductive age

[1] Downloaded from VMNIS on March 29, 2022
[2] Reported from the 'Date' column of the VMNIS ranging from 2000–2019.
[3] Adjustment for inflammation summarized based on information under 'Indicator Comments' in the VMNIS database. Categorical adjustments refer to the method using internal correction factor(s) suggested by Thurnham et al. (2005; 2010) and BRINDA refers to adjustments using linear regression proposed by the Biomarkers Reflecting Inflammation and Nutritional Determinants of Anemia (BRINDA) project (McDonald et al., 2020).
