# Supplementary material for: Comparison of Published Estimates of the National Prevalence of Iron, Vitamin A, and Zinc Deficiency and Sources of Inconsistencies
Source: Adv Nutr. 2023 Aug 25;14(6):1466–78. doi: 10.1016/j.advnut.2023.08.011 (PMC10721533; doi:10.1016/j.advnut.2023.08.011)
Supplement: Multimedia component2 [file mmc2.pdf]

Comparison of published estimates of the national prevalence of iron, vitamin A and zinc deficiency and sources of inconsistencies

Supplemental Tables S7 – S12

by Hess SY, Wessells KR, Haile D, Rogers LM, Tan X, Barros JG, Bourassa MW, Gorstein J, Brown KH

	Page
**Supplemental Table S7.** Prevalence estimates of iron deficiency or inadequate dietary iron intake among young children in countries with nationally representative survey results	2
**Supplemental Table S8.** Prevalence estimates of iron deficiency or inadequate dietary iron intake among women of reproductive age in countries with nationally representative survey results	8
**Supplemental Table S9.** Prevalence estimates of vitamin A deficiency or inadequate dietary vitamin A intake among young children in countries with nationally representative survey results	15
**Supplemental Table S10.** Prevalence estimates of vitamin A deficiency or inadequate dietary vitamin A intake among women of reproductive age in countries with nationally representative survey results	22
**Supplemental Table S11.** Prevalence estimates of zinc deficiency or inadequate dietary zinc intake among young children in countries with nationally representative survey results	27
**Supplemental Table S12.** Prevalence estimates of zinc deficiency among women of reproductive age in countries with nationally representative survey results	29

Supplemental Table S7. Prevalence estimates of iron deficiency or inadequate dietary iron intake among young children in countries with nationally representative survey results[1]

Country survey code	Survey (Year)[2]	Age Range (mo)	Ferritin						sTfR						GBD 2019 Study[9]	Dietary iron inadequacy[10]
			VMNIS			BRINDA			VMNIS			BRINDA				
			n	Prevalence (%)	Adjustment[8]	*n*	Prevalence, unadjusted (%)	Prevalence adjusted IRC – CRP + AGP (%)	*n*	Prevalence (%)	Adjustment[8]	*n*	Prevalence, unadjusted (%)	Prevalence adjusted IRC – AGP (%)	Prevalence (%)	Prevalence (%)
AFG-13	Afghanistan (2013)	6–59	–	26.1	Categorical for elevated CRP and AGP	665[3]	22.0 (17.9, 26.6)[3]	24.2 (20.1, 28.8)[3]	–	–	–	–	–	–	23.0 (19.4, 26.4)	–
ARG-05	Argentina (2005)	6–23	–	35.3	Ferritin cut-off <30 µg/L when leukocyte count >15 x 1000 mm^3	–	–	–	–	–	–	–	–	–	26.2 (19.4, 33.7)	–
AZE-13	Azerbaijan (2013)	6–59	1111	15	Categorical for elevated CRP and AGP	1053[3]	13.9 (11.5, 16.7)[3]	22.4 (18.9, 26.2)[3]	–	–	–	1053[3]	7.9 (6.1, 10.1)[3]	6.7 (5.3, 8.5)[3]	22.2 (18.4, 26.2)	–
BFA-10	Burkina Faso (2010)	6–59	–	–	–	125[3]	0 (0, 0)[3]	8.7 (3.5, 19.9)[3]	–	–	–	125[3]	85.8 (77.4, 91.4)[3]	15.5 (8.4, 27.0)[3]	44.5 (42.2, 47.0)	–
BGD-11-12	Bangladesh (2011–2012)	6–59	468	10.7	Categorical for elevated CRP and AGP	467[3]	9.8 (5.9, 15.7)[3]	13.6 (9.1, 19.9)[3]	–	–	–	–	–	–	36.6 (30.7, 42.7)[6]	–
BLZ-11	Belize (2011)	6–59	636	13.2	Samples with AGP >1 g/L excluded	–	–	–	–	–	–	–	–	–	31.9 (21.0, 44.0)	–
CAN-11	Canada (2011)	36–60	487	3.2	Not specified	–	–	–	–	–	–	–	–	–	4.4 (1.8, 8.2)	–
CIV-07	Côte d'Ivoire (2007)	6–59	783	15.5	Categorical for elevated CRP and AGP	746[3]; 733[4]	11.7 (9.2, 14.8)[3]; 11.7 (8.9, 14.5)[4]	39.5 (35.5, 43.6)[3]; 40.9 (36.7, 45.1)[4]	783	11.2	–	746[3]; 733[5]	23.9 (20.1, 28.1)[3]; 24.2 (20.2, 28.2)[5]	8.4 (6.4, 11.1)[3]; 8.6 (6.2, 11.0)[5]	41.3 (37.7, 44.0)	–
CMR-09	Cameroon (2009)	12–59; 6-22	838	20.6	Categorical for elevated CRP and AGP	792[3]; 774[4]	14.8 (12.3, 17.8)[3]; 14.9 (12.1, 17.6)[4]	34.9 (30.8, 39.2)[3]; 35.0 (30.8, 39.1)[4]	838	68.4	Unadjusted	792[3]; 774[5]	68.5 (63.9, 72.8)[3]; 68.2 (63.7, 72.7)[5]	44.7 (40.3, 49.1)[3]; 44.9 (40.4, 49.4)[5]	37.7 (34.4, 40.9)	69.9 ± 1.4
COL-05	Colombia (2005)	12–48	4188	12.5	Samples with CRP 12 mg/L were excluded	–	–	–	–	–	–	–	–	–	17.6 (11.0, 24.6)	–
COL-10	Colombia (2010)	12–48	3542	10.6	Samples with CRP 12 mg/L were excluded	3866[4]	10.1 (8.8, 11.4)[4]	–	–	–	–	–	–	–	15.2 (8.9, 21.9)	–
CRI-09	Costa Rica (2009)	12–72	399	5.9	Not specified	–	–	–	–	–	–	–	–	–	14.8 (8.4, 21.9)	–

Supplemental Table S7, continued

Country survey code	Survey (Year)[2]	Age Range (mo)	Ferritin						sTfR						GBD 2019 Study[9]	Dietary iron inadequacy[10]
			VMNIS			BRINDA			VMNIS			BRINDA				
			n	Prevalence (%)	Adjustment[8]	*n*	Prevalence, unadjusted (%)	Prevalence adjusted IRC – CRP + AGP (%)	*n*	Prevalence (%)	Adjustment[8]	*n*	Prevalence, unadjusted (%)	Prevalence adjusted IRC – AGP (%)	Prevalence (%)	Prevalence (%)
DOM-09	Dominican Republic (2009)	6–59	330	27.4	Samples with AGP >1 g/L excluded	–	–	–	–	–	–	–	–	–	18.0 (13.4, 22.9)	–
ECU-12	Ecuador (2012)	6–59	2045	9.9	Unadjusted, also presented w/o inflammation	–	–	–	–	–	–	–	–	–	10.2 (7.7, 13.2)	–
ETH-15	Ethiopia (2015)	6–59	1140	17.8	Categorical for elevated CRP and AGP	–	–	–	1138	29.6	Not specified	–	–	–	37.5 (34.9, 39.9)	–
GBR-12	United Kingdom of Great Britain and Northern Ireland (2012)	12–36	43	35.1	Not specified	–	–	–	–	–	–	–	–	–	13.2 (8.1, 21.0)	–
GBR-14	United Kingdom of Great Britain and Northern Ireland (2014)	12–36	31	31	Not specified	–	–	–	–	–	–	–	–	–	13.0 (7.9, 20.4)	–
GEO-09	Georgia (2009)	12–59	1648	0.1	Samples with CRP >5 mg/L excluded	2142[4]	0.3 (<0.1, 0.5)[4]	–	–	–	–	–	–	–	26.7 (17.0, 37.4)	–
GHA-17	Ghana (2017)	6–59	1165	21.5	Categorical for elevated CRP and AGP	–	–	–	–	–	–	–	–	–	44.8 (38.1, 50.7)	–
GMB-18	Gambia (2018)	6–59	1012	59	BRINDA using CRP and AGP	–	–	–	–	–	–	–	–	–	56.8 (51.4, 61.3)	–
GTM-10	Guatemala (2010)	6–59	985	18.6	Unadjusted, also presented with ferritin cut-off >30 µg/L when AGP >1 g/L	–	–	–	–	–	–	–	–	–	25.3 (20.7, 30.4)	–
GTM-13	Guatemala (2013)	6–59	858	8.9	Categorical for elevated CRP and AGP	–	–	–	–	–	–	–	–	–	27.5 (22.4, 33.4)	–

Supplemental Table S7, continued

Country survey code	Survey (Year)[2]	Age Range (mo)	Ferritin						sTfR						GBD 2019 Study[9]	Dietary iron inadequacy[10]
			VMNIS			BRINDA			VMNIS			BRINDA				
			n	Prevalence (%)	Adjustment[8]	*n*	Prevalence, unadjusted (%)	Prevalence adjusted IRC – CRP + AGP (%)	*n*	Prevalence (%)	Adjustment[8]	*n*	Prevalence, unadjusted (%)	Prevalence adjusted IRC – AGP (%)	Prevalence (%)	Prevalence (%)
GTM-15	Guatemala (2015)	6–59	682	12.9	BRINDA using CRP and AGP	–	–	–	682	3.5	BRINDA using CRP and AGP	–	–	–	28.7 (23.0, 35.2)	–
GTM-16	Guatemala (2016)	6–59	569	10.8	BRINDA using CRP and AGP	–	–	–	569	2.8	BRINDA using CRP and AGP	–	–	–	29.1 (23.1, 35.8)	–
GTM-18	Guatemala (2018)	6–59	551	16.6	BRINDA using CRP and AGP	–	–	–	538	13.3	BRINDA using CRP and AGP	–	–	–	29.5 (22.9, 36.3)	–
IND-18	India (2018)	12–48	7838	31.9	Samples with CRP >5 mg/L excluded	–	–	–	–	–	–	–	–	–	41.1 (39.4, 42.8)	–
IRQ-12	Iraq (2012)	12–59	2087	14.4	Not specified	–	–	–	–	–	–	–	–	–	27.2 (17.8, 38.3)	–
JOR-02	Jordan (2002)	12–59	1066	26.1	Not specified	–	–	–	–	–	–	–	–	–	23.0 (19.8, 26.2)	–
JOR-10	Jordan (2010)	12–59	940	13.7	Unadjusted	–	–	–	–	–	–	–	–	–	20.8 (17.7, 24.1)	–
KEN-11	Kenya (2011)	6–59	918	21.8	Categorical for elevated CRP and AGP	–	–	–	–	–	–	–	–	–	24.0 (22.1, 25.9)	67
KGZ-09	Kyrgyzstan (2009)	6–59	1413	44.6	Excluded samples with elevated CRP and/or AGP	–	–	–	1413	33.6	Excluded samples with elevated CRP and/or AGP	–	–	–	35.6 (30.6, 40.7)	–
KGZ-13	Kyrgyzstan (2013)	6–29	2148	48.2	BRINDA using CRP and AGP	–	–	–	2148	37.4	BRINDA using AGP	–	–	–	34.7 (29.5, 40.0)	–
KHM-14	Cambodia (2014)	6–71; 6–59	793	3.3	Categorical for elevated CRP and AGP	665[3]	3.8 (2.4, 5.9)[3]	4.9 (3.4, 7.1)[3]	793	47.5	Not specified	665[3]	49.3 (44.2, 54.5)[3]	29.8 (26.5, 33.4) [3]	39.4 (34.4, 44.3)	–
LAO-06	Lao People's Democratic Republic (2006)	6–59	483	18.4	Categorical for elevated CRP and AGP	481[3, 4]	16.6 (12.8, 21.2)[3]; 16.6 (12.4, 20.7)[4]	26.4 (21.9, 31.4)[3]; 27.0 (22.3, 31.8)[4]	483	44.7	Not specified	481[3, 5]	4.1 (2.4, 6.9)[3]; 4.1 (1.9, 6.2)[5]	3.3 (1.8, 6.1) [3]; 3.3 (1.3, 5.4)[5]	26.0 (21.6, 30.6)	–
LBR-11	Liberia (2011)	6–35	1416	29.8	Categorical for elevated CRP and AGP	1434[3, 4]	20.4 (18.0, 23.2)[3]; 20.4 (17.8, 23.0)[4]	55.6 (51.5, 59.6)[3, 4]	–	–	–	1434[3, 5]	76.7 (73.3, 79.9)[3]; 76.7 (73.5, 80.0)[5]	55.9 (52.2, 59.5) [3,5]	38.0 (33.3, 42.7)	–

Country survey code	Survey (Year)[2]	Age Range (mo)	Ferritin						sTfR						GBD 2019 Study[9]	Dietary iron inadequacy[10]
			VMNIS			BRINDA			VMNIS			BRINDA				
			n	Prevalence (%)	Adjustment[8]	*n*	Prevalence, unadjusted (%)	Prevalence adjusted IRC – CRP + AGP (%)	*n*	Prevalence (%)	Adjustment[8]	*n*	Prevalence, unadjusted (%)	Prevalence adjusted IRC – AGP (%)	Prevalence (%)	Prevalence (%)
LKA-12	Sri Lanka (2012)	6–59	5766	33.6	Samples with CRP >5 mg/L excluded	–	–	–	–	–	–	–	–	–	13.7 (10.5, 16.9)	–
MAR-00	Morocco (2000)	6–59	644	50.8	Not specified	–	–	–	–	–	–	–	–	–	29.8 (21.1, 39.1)	–
MDV-08	Maldives (2008)	0–60	1268	57.3	Unadjusted, also presented adjusted for CRP >65 mg/L	–	–	–	–	–	–	–	–	–	37.0 (26.8, 47.1)	–
MEX-06	Mexico (2006)	12–48	1710	26	Not specified	1590[4]	23.4 (20.3, 26.6)[4]	–	1707	15.7	Not specified	–	–	–	14.8 (13.6, 16.2)	–
MEX-12	Mexico (2012)	12–59	2591	13.9	Categorical for elevated CRP	2538[4]	13.5 (11.1, 15.9)[4]	–	–	–	–	–	–	–	15.2 (14.0, 16.7)	4.8 ± 0.2
MNG-04	Mongolia (2004)	6–59	386	22.3	Not specified	–	–	–	–	–	–	–	–	–	25.0 (21.8, 28.3)	–
MNG-10	Mongolia (2010)	6–59	433	21.4	Samples with elevated CRP excluded	–	–	–	–	–	–	–	–	–	21.2 (16.6, 25.9)	–
MOZ-02	Mozambique (2002)	6–59	–	–	–	–	–	–	706	36	Not specified	–	–	–	42.3 (37.8, 46.2)	–
MWI-01	Malawi (2001)	6–36	–	–	–	–	–	–	365	61.5	Not specified	–	–	–	43.7 (37.5, 49.3)	–
MWI-09	Malawi (2009)	6–59	455	10.3	Samples with CRP >5 mg/L or AGP >1 g/L excluded	–	–	–	980	50.9	Not specified	–	–	–	41.5 (36.9, 45.9)	–
MWI-16	Malawi (2016)	6–59	1102	21.7	BRINDA using CRP and AGP	1102[3]	10.7 (7.8, 14.6)[3]	21.9 (17.0, 27.7)[3]	1102	55.2	Not specified	1102[3]	55.2 (49.0, 61.3)[3]	42.4 (36.7, 48.4)[3]–	45.3 (40.0, 50.6)	–
NGA-01	Nigeria (2001)	6–59	3091	19.4	Not specified	–	–	–	–	–	–	–	–	–	43.7 (40.7, 46.6)	–
NGI-12	Nigeria (2012)	6–59	–	–	–	547[3]	5.1 (3.4, 7.7)[3]	18.3 (14.1, 23.4)[3]	–	–	–	547[3]	59.8 (53.8, 65.5)[3]	30.0 (24.7, 35.9)[3]–	45.7 (43.3, 48.1)	–
NIC-04	Nicaragua (2004)	6–59	345	32.6	Samples with AGP >1 g/L excluded	–	–	–	–	–	–	–	–	–	14.2 (12.4, 16.3)	–
NIC-05	Nicaragua (2005)	6–59	368	42.2	Samples with AGP >1 g/L excluded	957[4]	33.2 (28.6, 37.9)[4]	–	–	–	–	–	–	–	13.9 (11.7, 16.4)	–
NIC-07	Nicaragua (2007)	6–59	536	45.1	Samples with AGP >1 g/L excluded	–	–	–	–	–	–	–	–	–	13.4 (10.8, 16.3)	–
NPL-16	Nepal (2016)	6–59	1651	27.6	BRINDA using CRP and AGP	–	–	–	1651	63.3	BRINDA using CRP and AGP	–	–	–	40.1 (32.8, 47.6)	–

Country survey code	Survey (Year)[2]	Age Range (mo)	Ferritin						sTfR						GBD 2019 Study[9]	Dietary iron inadequacy[10]
			VMNIS			BRINDA			VMNIS			BRINDA				
			n	Prevalence (%)	Adjustment[8]	*n*	Prevalence, unadjusted (%)	Prevalence adjusted IRC – CRP + AGP (%)	*n*	Prevalence (%)	Adjustment[8]	*n*	Prevalence, unadjusted (%)	Prevalence adjusted IRC – AGP (%)	Prevalence (%)	Prevalence (%)
OMN-04	Oman (2004)	6–59	199	18.5	Samples with CRP >10 mg/L excluded	–	–	–	–	–	–	–	–	–	37.0 (30.5, 43.8)	–
PAK-01	Pakistan (2001)	6–59	5594	66.5	Not specified	–	–	–	–	–	–	–	–	–	41.3 (33.7, 48.9)	–
PAK-11	Pakistan (2011)	6–59	–	–	–	7221[4]	46.9 (45.4, 48.5)[4]	–	–	–	–	–	–	–	39.9 (31.9, 47.9)	–
PNG-05	Papua New Guinea (2005)	6–59	–	–	–	–	–	–	872	27.8	Not specified	868[3, 5]	25.1 (20.9, 29.8)[3]; 25.1 (20.7, 29.5)[5]	10.5 (7.4, 14.6)[3]; 10.5 (6.9, 14.0)[5]	33.7 (30.5, 37.4)	–
RWA-10	Rwanda (2010)	6–59	–	–	–	576[3]	5.3 (3.5, 7.8)[3]	6.2 (4.3, 8.9)[3]	–	–	–	576[3]	3.1 (1.8, 5.2)[3]	2.5 (1.5, 4.2)[3]	30.0 (26.7, 33.4)	–
SEN-10	Senegal (2010)	12–59	1431	63.2	Categorical for elevated CRP and AGP	–	–	–	–	–	–	–	–	–	55.8 (53.3, 58.6)	–
SLE-13	Sierra Leone (2013)	6–59	654	5.2	Categorical for elevated CRP and AGP	–	–	–	–	–	–	–	–	–	42.7 (40.2, 45.1)	–
TJK-03	Tajikistan (2003)	6–59	–	–	–	–	–	–	1252	38.8	Not specified	–	–	–	27.9 (24.7, 31.6)	–
TJK-09	Tajikistan (2009)	6–59	1805	9.7	Samples with CRP >5 mg/L excluded	–	–	–	2104	8.6	Not specified	–	–	–	25.3 (21.8, 29.0)	–
TLS-13	Timor-Leste (2013)	6–59	547	20.5	Categorical for elevated CRP and AGP	–	–	–	547	51.7	Not specified	–	–	–	37.1 (31.8, 42.5)	–
TZA-10	United Republic of Tanzania (2010)	6–59	–	–	–	–	–	–	6397	35.3	Not specified	–	–	–	39.1 (34.8, 43.0)	–
USA-03-06	United States of America (2003–2006)	6–59	–	–	–	1138[4]	10.5 (7.9, 13.2)[4]	–	–	–	–	–	–	–	4.7 (3.8, 5.8)[7]	–
UZB-17	Uzbekistan (2017)	6–59	1736	54.7	BRINDA using CRP and AGP	–	–	–	–	–	–	–	–	–	45.0 (35.8, 54.5)	–
VMN-10	Viet Nam (2010)	10–75	568	12.9	Categorical for elevated CRP	–	–	–	–	–	–	–	–	–	16.6 (11.6, 21.8)	–

Supplemental Table S7, continued

Country survey code	Survey (Year)[2]	Age Range (mo)	Ferritin						sTfR						GBD 2019 Study[9]	Dietary iron inadequacy[10]
			VMNIS			BRINDA			VMNIS			BRINDA				
			n	Prevalence (%)	Adjustment[8]	*n*	Prevalence, unadjusted (%)	Prevalence adjusted IRC – CRP + AGP (%)	*n*	Prevalence (%)	Adjustment[8]	*n*	Prevalence, unadjusted (%)	Prevalence adjusted IRC – AGP (%)	Prevalence (%)	Prevalence (%)
ZAF-05	South Africa (2005)	12–60	821	19.7	Not specified	–	–	–	–	–	–	–	–	–	22.7 (18.8, 26.7)	–
ZAF-12	South Africa (2012)	0–59	454	10	Not specified	–	–	–	–	–	–	–	–	–	15.7 (12.1, 19.1)	–
ZMB-09	Zambia (2009)	6–59	–	–	–	410[3]	5.4 (2.7, 10.2)[3]	16.6 (11.5, 23.3)[3]	–	–	–	–	–	–	44.8 (39.7, 50.1)	–
ZWE-13	Zimbabwe (2013)	6–59	–	–	–	–	–	–	1701	72.2	Not specified	–	–	–	35.5 (30.7, 40.4)	–

Abbreviations: AGP, α 1-acid-glycoprotein; BRINDA, Biomarkers Reflecting Inflammation and Nutritional Determinants of Anemia; CRP, C-reactive protein; df, degrees of freedom; GBD, Global Burden of Disease Study; IRC, Internal Regression Correction; PSC, preschool-age children; QE, Test for Residual Heterogeneity; sTfR, soluble transferrin receptor; VMNIS, Vitamin Mineral Nutrition Information System by the World Health Organization; –, Not Available

[1] Prevalence values are either % or % (95% CI).
[2] Reported from the 'Date' column of the VMNIS database or obtained from selected BRINDA Project Publications, ranging from 2000–2019; survey data on the same row that are also reported from selected BRINDA Project Publications are from the same nationally representative surveys as reported in VMNIS, but may have slightly different 'Before BRINDA Adjustment' and 'After BRINDA Adjustment' sample size and prevalence due to data availability and adjustment methods.
[3] Namaste *et al.* (2020): Depleted iron stores were defined as a ferritin concentration <12 μg/L in PSC. Iron-deficient erythropoiesis was defined as a sTfR concentration >8.3 mg/L in PSC. Updated analysis including additional surveys.
[4] Namaste *et al.* (2017): Depleted iron stores were defined as a ferritin concentration <12 μg/L in PSC.
[5] Rohner *et al.* (2017): Elevated sTfR defined as >8.3 mg/L.
[6] Bangladesh (2011–2012) survey reported prevalence for 2011; Additional reported prevalence (2012): 36.3 (30.4, 42.5).
[7] United States of America (2003–2006) survey reported prevalence for 2006; Additional reported prevalence (2003): 4.8 (3.8, 5.9), (2004): 4.7 (3.7, 5.8), and (2005): 4.7 (3.7, 5.8).
[8] Adjustment for inflammation summarized based on information under 'Indicator Comments' in the VMNIS database. Categorical adjustments refer to the method using internal correction factor(s) suggested by Thurnham et al. (2005; 2010) and BRINDA refers to adjustments using linear regression proposed by the Biomarkers Reflecting Inflammation and Nutritional Determinants of Anemia (BRINDA) project (Namaste et al., 2017).
[9] Dietary iron deficiency estimated in the GBD 2019 Study for children 1-4 years of age (GBD 2019 Diseases and Injuries Collaborators) as a cause of anemia, modeled based on hemoglobin concentration in the GBD 2019 Study. These estimates represent only dietary iron deficiency associated with anemia and do not include iron deficiency without anemia.
[10] For Cameroon (2009), the prevalence of iron inadequacy was estimated based on absorbable iron predicted by Armah et al algorithm (7); in Kenya (2011), 8% bioavailability was assumed for iron. In Mexico (2012), the prevalence of inadequate iron intake was estimated with the use of the full-probability approached and assuming 18% iron bioavailability (8).

Supplemental Table S8. Prevalence estimates of iron deficiency or inadequate dietary iron intake among women of reproductive age in countries with nationally representative survey results[1]

Country survey code	Survey (Year)[2]	Age Range (yr)	Ferritin						sTfR						GBD 2019 Study[9]	Dietary iron inadequacy[10]
			VMNIS			BRINDA			VMNIS							
			n	Prevalence (%)	Adjustment[8]	Prevalence (%)	Unadjusted	IRC – CRP + AGP	*n*	Prevalence (%)	Adjustment[8]	*n*	Unadjusted	IRC – AGP	Prevalence (%)	Prevalence (%)
AFG-13	Afghanistan (2013)	15–49	–	24	Categorical for elevated CRP and AGP	1050[3]	25.9 (21.1, 31.3)[3]	34.0 (28.6, 39.7)[3]	–	–	–	–	–	–	4.8 (3.8, 6.0)	–
ARG-05	Argentina (2005)	10–49	5322	18.7	Not specified	–	–	–	–	–	–	–	–	–	9.8 (7.7, 12.1)	–
AUS-12	Australia (2012)	16–44	1884	12.1	Samples with CRP >10 mg/L excluded	–	–	–	2099	7.2	Not specified	–	–	–	4.5 (2.7, 6.7)	–
AUT-12	Austria (2012)	18–64	204	17.2	Not specified	–	–	–	–	–	–	–	–	–	2.8 (1.5, 4.6)	–
AZE-13	Azerbaijan (2013)	15–49	2706	34.1	Categorical for elevated CRP and AGP	2656[3]	30.7 (28.5, 33.1)[3]	44.0 (41.5, 46.5)[3]	–	–	–	2656[3]	18.5 (16.7, 20.4)[3]	16.2 (14.5, 17.9)3	19.7 (17.3, 22.0)	–
BFA-10	Burkina Faso (2010)	15–49	–	–	–	129[3]	3.1 (1.1, 8.2)[3]	11.1 (5.9, 19.8)[3]	–	–	–	129[3]	80.8 (65.2, 90.4)[3]	43.1 (32.5, 54.4) [3]–	7.9 (6.9, 9.0)	–
BGD-11-12	Bangladesh (2011–2012)	15–49	882	7.1	Categorical for elevated CRP and AGP	876[3]	7.6 (5.2, 10.9)[3]	9.5 (6.8, 13.1)[3]	–	–	–	–	–	–	18.1 (15.2, 21.4)[4]	–
BHR-02	Bahrain (2002)	14–49	384	35.4	Not specified	–	–	–	–	–	–	–	–	–	21.1 (19.0, 23.2)	–
BLZ-11	Belize (2011)	15–49	485	17.8	Samples with AGP >1 g/L excluded	–	–	–	–	–	–	–	–	–	16.6 (13.0, 20.4)	–
CAN-11	Canada (2011)	20–49	–	9.1	Not specified	–	–	–	–	–	–	–	–	–	4.4 (2.7, 6.5)	–
CIV-07	Côte d'Ivoire (2007)	15–49	906	16.7	Categorical for elevated CRP and AGP	834[3]; 816[5]	13.4 (10.8, 16.5)[3]; 13.6 (10.7, 16.4)[5]	22.5 (19.3, 26.0)[3]; 22.3 (18.9, 25.8)[5]	907	6.7	Not specified	834[3]; 816[6]	31.1 (27.2, 35.4)[3]; 31.1 (27.0, 35.3)[6]	21.4 (18.3, 24.8)[3]; 21.6 (18.2, 25.0)[6]	6.9 (6.2, 7.7)	–
CMR-09	Cameroon (2009)	15–49	872	15.3	Categorical for elevated CRP and AGP	760[3]; 751[5]	12.8 (10.4, 15.7)[3]; 12.9 (10.2, 15.6)[5]	19.3 (16.1, 22.9)[3]; 19.2 (15.8, 22.6)[5]	872	31.9	Unadjusted	760[3]; 751[6]	33.8 (29.2, 38.7)[3]; 34.0 (29.2, 38.8)[6]	20.1 (16.6, 24.2)[3]; 20.2 (16.4, 23.9)[6]	7.2 (6.4, 8.0)	94.9 ± 0.7
COL-05	Colombia (2005)	13–49	3483	15.9	Samples with CRP >12 mg/L excluded	–	–	–	–	–	–	–	–	–	6.7 (4.5, 9.1)	–

Country survey code	Survey (Year)[2]	Age Range (yr)	Ferritin						sTfR						GBD 2019 Study[9]	Dietary iron inadequacy[10]
			VMNIS			BRINDA			VMNIS							
			n	Prevalence (%)	Adjustment[8]	Prevalence (%)	Unadjusted	IRC – CRP + AGP	*n*	Prevalence (%)	Adjustment[8]	*n*	Unadjusted	IRC – AGP	Prevalence (%)	Prevalence (%)
COL-10	Colombia (2010)	13–49; 15–49	9600	17.1	Samples with CRP >12 mg/L excluded	9083[5]	22.9 (21.8, 24.1)[5]	–	–	–	–	–	–	–	4.7 (3.0, 6.6)	–
CRI-09	Costa Rica (2009)	15–44	873	8.7	Not specified	–	–	–	–	–	–	–	–	–	7.5 (5.4, 9.8)	–
DOM-09	Dominican Republic (2009)	15–49	541	49.4	Samples with AGP >1 g/L excluded	–	–	–	–	–	–	–	–	–	12.7 (10.9, 14.7)	–
ECU-12	Ecuador (2012)	20–49	6957	14.6	Not specified	–	–	–	–	–	–	–	–	–	5.9 (4.7, 7.3)	–
ETH-05	Ethiopia (2005)	15–49	970	29.4	Unadjusted	–	–	–	–	–	–	–	–	–	4.9 (4.4, 5.5)	–
ETH-15	Ethiopia (2015)	15–49	1700	10	Categorical for elevated CRP and AGP	–	–	–	1726	16.4	Not specified	–	–	–	4.3 (3.9, 4.8)	–
FJI-04	Fiji (2004)	15–44	738	22.9	Unadjusted	–	–	–	–	–	–	–	–	–	23.2 (21.2, 25.3)	–
FJI-10	Fiji (2010)	15–45	869	7.9	Not specified	–	–	–	–	–	–	–	–	–	21.9 (19.8, 24.1)	–
FRA-07	France (2007)	18–49	–	13.5	Not specified	–	–	–	–	–	–	–	–	–	1.5 (0.8, 2.5)	–
GBR-01	United Kingdom of Great Britain and Northern Ireland (2001)	19–64	670	11	Not specified	–	–	–	–	–	–	–	–	–	4.6 (3.7, 5.7)	–
GBF-10	United Kingdom of Great Britain and Northern Ireland (2010)	19–64	348	16	Not specified	–	–	–	–	–	–	–	–	–	4.0 (3.2, 4.8)	–
GBR-12	United Kingdom of Great Britain and Northern Ireland (2012)	19–64	775	15.5	Not specified	–	–	–	761	–	Not specified	–	–	–	3.9 (3.1, 4.8)	–

Supplemental Table S8, continued

Country survey code	Survey (Year)[2]	Age Range (yr)	Ferritin						sTfR						GBD 2019 Study[9]	Dietary iron inadequacy[10]
			VMNIS			BRINDA			VMNIS							
			n	Prevalence (%)	Adjustment[8]	Prevalence (%)	Unadjusted	IRC – CRP + AGP	*n*	Prevalence (%)	Adjustment[8]	*n*	Unadjusted	IRC – AGP	Prevalence (%)	Prevalence (%)
GBR-14	United Kingdom of Great Britain and Northern Ireland (2014)	19–64	323	10	Not specified	–	–	–	–	–	–	–	–	–	3.9 (3.0, 4.9)	–
GBR-16	United Kingdom of Great Britain and Northern Ireland (2016)	19–64	297	12	Not specified	–	–	–	–	–	–	–	–	–	3.9 (2.9, 5.1)	–
GBR-19	United Kingdom of Great Britain and Northern Ireland (2019)	19–64	427	15	Not specified	–	–	–	–	–	–	–	–	–	3.9 (2.7, 5.2)	–
GEO-09	Georgia (2009)	15–49	–	1.6	Samples with CRP >5 mg/L excluded	1688[5]	1.4 (0.8, 2.1)[5]	–	–	–	–	–	–	–	15.2 (13.4, 17.0)	–
GHA-17	Ghana (2017)	15–49	987	13.7	Categorical for elevated CRP and AGP	–	–	–	–	–	–	–	–	–	12.8 (10.1, 15.3)	–
GMB-18	Gambia (2018)	15–49	1401	41.4	BRINDA using CRP and AGP	–	–	–	–	–	–	–	–	–	16.6 (14.3, 19.2)	–
GTM-10	Guatemala (2010)	15–49	1418	11.2	Unadjusted, also presented with ferritin cut-off >30 µg/L when AGP >1 g/L	–	–	–	–	–	–	–	–	–	6.0 (4.7, 7.4)	–
GTM-13	Guatemala (2013)	15–49	1621	9.9	Categorical for elevated CRP and AGP	–	–	–	–	–	–	–	–	–	5.9 (4.7, 7.4)	–
GTM-15	Guatemala (2015)	15–49	1515	14.7	BRINDA using CRP and AGP	–	–	–	–	–	–	–	–	–	5.9 (4.6, 7.5)	–
GTM-16	Guatemala (2016)	15–49	1485	13.8	BRINDA using CRP and AGP	–	–	–	1484	1.6	BRINDA using CRP and AGP	–	–	–	6.1 (4.7, 7.6)	–

Supplemental Table S8, continued

Country survey code	Survey (Year)[2]	Age Range (yr)	Ferritin						sTfR						GBD 2019 Study[9]	Dietary iron inadequacy[10]
			VMNIS			BRINDA			VMNIS							
			n	Prevalence (%)	Adjustment[8]	Prevalence (%)	Unadjusted	IRC – CRP + AGP	*n*	Prevalence (%)	Adjustment[8]	*n*	Unadjusted	IRC – AGP	Prevalence (%)	Prevalence (%)
IRQ-12	Iraq (2012)	15–49	1152	24.5	Not specified	–	–	–	–	–	–	–	–	–	7.9 (5.8, 10.1)	–
JOR-02	Jordan (2002)	15–49	1303	38.7	Not specified	–	–	–	–	–	–	–	–	–	12.5 (11.0, 13.8)	–
JOR-10	Jordan (2010)	15–49	2035	35.1	Unadjusted	–	–	–	–	–	–	–	–	–	10.6 (9.5, 11.7)	–
KEN-11	Kenya (2011)	15–49	633	21.3	Categorical for elevated CRP and AGP	–	–	–	–	–	–	–	–	–	6.9 (6.6, 7.3)	91
KGZ-09	Kyrgyzstan (2009)	17–	1026	51	Excluded samples with elevated CRP and/or AGP	–	–	–	1026	23.9	Samples with elevated CRP or AGP excluded	–	–	–	20.3 (17.5, 23.0)	–
KHM-14	Cambodia (2014)	15–49	738	2.55	Categorical for elevated CRP and AGP	705[3]	2.7 (1.5, 4.6)[3]	3.5 (2.2, 5.6)[3]	738	33.89	Not specified	705[3]	33.5 (26.5, 41.3)[3]	11.2 (8.7, 14.4)[3]–	19.1 (16.3, 21.7)	–
Lao-06	Lao People's Democratic Republic (2006)	15–49	818	23.2	Categorical for elevated CRP and AGP	816[3, 5]	22.7 (17.9, 28.3)[3]; 22.7 (17.5, 27.9)[5]	26.4 (20.8, 32.9)[3]; 26.8 (20.9, 32.8)[5]	818	28	Not specified	816[3, 6]	5.8 (3.8, 8.6)[3]; 5.8 (3.5, 8.0)[6]	3.9 (2.6, 5.8)[3]; 3.9 (2.4, 5.5)[6]	13.8 (12.7, 15.0)	–
LBN-03	Lebanon (2003)	15–45	470	27.2	Not specified	–	–	–	–	–	–	–	–	–	4.5 (3.7, 5.3)	–
LBR-11	Liberia (2011)	15–49	1911	19.6	Categorical for elevated CRP and AGP	1942[3]; 1875[5]	17.9 (15.6, 20.4)[3]; 17.9 (15.5, 20.3)[5]	28.4 (25.3, 31.7)[3]; 28.6 (25.2, 31.9)[5]	–	–	–	1942[3]; 1875[6]	28.3 (25.3, 31.5)[3]; 28.6 (25.4, 31.7)[6]	21.9 (19.4, 24.7)[3]; 22.1 (19.5, 24.8)[6]	7.3 (6.1, 8.5)	–
MAR-00	Morocco (2000)	15–49	445	56.9	Not specified	–	–	–	–	–	–	–	–	–	11.5 (9.1, 14.7)	–
MDV-08	Maldives (2008)	15–49	1304	38.4	Unadjusted, also presented adjusted for CRP >65 mg/L	–	–	–	–	–	–	–	–	–	19.7 (16.6, 23.1)	–
MEX-06	Mexico (2006)	20–; 15–49	3399	18.1	Not specified	3020[5]	27.5 (24.6, 30.3)[5]	–	3390	9.5	Not specified	–	–	–	4.7 (4.4, 5.1)	–
MEX-12	Mexico (2012)	20–49; 15–49	4136	29.4	Categorical for elevated CRP	3612[5]	27.9 (24.9, 30.9)[5]	–	–	–	–	–	–	–	5.1 (4.8, 5.5)	22.2 ± 0.5

Supplemental Table S8, continued

Country survey code	Survey (Year)[2]	Age Range (yr)	Ferritin						sTfR						GBD 2019 Study[9]	Dietary iron inadequacy[10]
			VMNIS			BRINDA			VMNIS							
			n	Prevalence (%)	Adjustment[8]	Prevalence (%)	Unadjusted	IRC – CRP + AGP	*n*	Prevalence (%)	Adjustment[8]	*n*	Unadjusted	IRC – AGP	Prevalence (%)	Prevalence (%)
MNG-04	Mongolia (2004)	15–49	408	13.7	Not specified	–	–	–	–	–	–	–	–	–	15.4 (13.9, 16.8)	–
MNG-10	Mongolia (2010)	15–49	767	28.2	Samples with elevated CRP excluded	–	–	–	–	–	–	–	–	–	11.5 (9.7, 13.4)	–
MOZ-02	Mozambique (2002)	Not specified	–	–	–	–	–	–	557	12.7	Not specified	–	–	–	6.8 (6.1, 7.5)	–
MWI-01	Malawi (2001)	15–45	–	–	–	–	–	–	346	32.4	Not specified	–	–	–	7.8 (6.6, 8.9)	–
MWI-09	Malawi (2009)	15–49	431	10.4	Samples with CRP >5 mg/L or AGP >1 g/L excluded	–	–	–	509	26.1	Not specified	–	–	–	7.2 (6.2, 8.3)	–
MWI-16	Malawi (2016)	15–49	752	15.1	BRINDA using CRP and AGP	776[3]	11.4 (8.5, 15.1)[3]	12.7 (9.7, 16.3)[3]	752	24.6	Not specified	776[3]	24.5 (20.9, 28.6)[3]	22.3 (18.6, 26.5)[3]–	9.3 (7.6, 11.1)	–
NGA-01	Nigeria (2001)	Not specified	3949	12.7	Not specified	–	–	–	–	–	–	–	–	–	6.3 (5.4, 7.2)	–
NGA-12	Nigeria (2012)	15–49	–	–	–	620[3]	8.7 (6.2, 12.2)[3]	15.5 (12.2, 19.5)[3]	–	–	–	620[3]	36.1 (32.3, 40.1)[3]	26.6 (22.8, 30.9)[3]–	10.8 (9.6, 12.0)	–
NIC-04	Nicaragua (2004)	15–49	310	30.3	Samples with AGP >1 g/L excluded	–	–	–	–	–	–	–	–	–	5.1 (4.5, 5.7)	–
NIC-05	Nicaragua (2005)	15–49	363	31.2	Samples with AGP >1 g/L excluded	–	–	–	–	–	–	–	–	–	4.8 (4.2, 5.5)	–
NIC-07	Nicaragua (2007)	15–49	–	33	Samples with AGP >1 g/L excluded	–	–	–	–	–	–	–	–	–	4.3 (3.7, 5.1)	–
NPL-16	Nepal (2016)	15–49	2129	18.7	BRINDA using CRP and AGP	–	–	–	2129	13.4	BRINDA using CRP and AGP	–	–	–	17.4 (15.1, 19.7)	–
NZL-09	New Zealand (2009)	15–	1878	8.4	Samples with CRP >8 mg/L excluded	–	–	–	–	–	–	–	–	–	3.1 (2.0, 4.5)	–
OMN-04	Oman (2004)	15–49	307	33.9	Samples with CRP >10 mg/L excluded	–	–	–	–	–	–	–	–	–	10.0 (7.9, 12.5)	–
PAK-01	Pakistan (2001)	15–49	447	48.3	Not specified	–	–	–	–	–	–	–	–	–	21.7 (18.8, 24.7)	–

Supplemental Table S8, continued

Country survey code	Survey (Year)[2]	Age Range (yr)	Ferritin						sTfR						GBD 2019 Study[9]	Dietary iron inadequacy[10]
			VMNIS			BRINDA			VMNIS							
			n	Prevalence (%)	Adjustment[8]	Prevalence (%)	Unadjusted	IRC – CRP + AGP	*n*	Prevalence (%)	Adjustment[8]	*n*	Unadjusted	IRC – AGP	Prevalence (%)	Prevalence (%)
PAK-11	Pakistan (2011)	15–49	–	–	–	5988[3]	35.6 (34.1, 37.1)[3]	41.8 (40.1, 43.4)[3]	–	–	–	4968[3]	16.5 (15.2, 17.9)[3]	14.2 (13.0, 15.6)[3]–	22.1 (19.3, 25.1)	–
PNG-05	Papua New Guinea (2005)	15–49	–	–	–	–	–	–	753	19.5	Not specified	746[3,6]	17.7 (14.4, 21.6)[3]; 17.7 (14.2, 21.3)[6]	7.8 (5.8, 10.3)[3]; 7.8 (5.5, 10.0)[6]	14.0 (11.8, 16.5)	–
RWA-10	Rwanda (2010)	15–49	–	–	–	596[3]	3.9 (2.5, 6.1)[3]	5.2 (3.4, 7.7)[3]	–	–	–	596[3]	3.0 (1.8, 4.8)[3]	1.5 (0.7, 3.0)[3]	5.5 (4.4, 6.5)	–
SEN-10	Senegal (2010)	15–49	983	44.3	Categorical for elevated CRP and AGP	–	–	–	–	–	–	–	–	–	13.0 (11.6, 14.5)	–
SLE-13	Sierra Leone (2013)	15–49	774	8.3	Categorical for elevated CRP and AGP	–	–	–	–	–	–	–	–	–	7.7 (6.4, 8.9)	–
TJK-03	Tajikistan (2003)	15–49	1195	12.8	Samples with CRP >5 mg/L excluded	–	–	–	1415	29.2	Not specified	–	–	–	13.4 (11.7, 15.1)	–
TJK-09	Tajikistan (2009)	15–49	2036	9.3	Samples with CRP >5 mg/L excluded	–	–	–	2073	4.8	Not specified	–	–	–	12.9 (11.4, 14.4)	–
TLS-13	Timor-Leste (2013)	14–60	592	21.3	Categorical for elevated CRP and AGP	–	–	–	592	29.4	Not specified	–	–	–	8.8 (6.6, 11.4)	–
TZA-10	United Republic of Tanzania (2010)	15–49	–	–	–	–	–	–	5871	28.9	Not specified	–	–	–	7.2 (6.3, 8.3)	–
USA-03-06	United States of America (2003–2006)	15–49	–	–	–	3183[5]	13.1 (11.4, 14.8)[5]	–	–	–	–	–	–	–	4.9 (4.2, 5.6)[7]	–
UZB-08	Uzbekistan (2008)	15–49	2582	47.5	Categorical for elevated CRP	–	–	–	–	–	–	–	–	–	33.8 (29.8, 37.6)	–
UZB-17	Uzbekistan (2017)	15–49	2077	48.1	BRINDA using CRP and AGP	–	–	–	–	–	–	–	–	–	31.3 (26.7, 35.7)	–
VNM-10	Viet Nam (2010)	15–49	1523	13.7	Categorical for elevated CRP	–	–	–	–	–	–	–	–	–	8.4 (6.5, 10.5)	–
ZAF-05	South Africa (2005)	16–35	1906	18.6	Not specified	–	–	–	–	–	–	–	–	–	10.1 (8.8, 11.5)	–

Supplemental Table S8, continued

Country survey code	Survey (Year)[2]	Age Range (yr)	Ferritin						sTfR						GBD 2019 Study[9]	Dietary iron inadequacy[10]
			VMNIS			BRINDA			VMNIS							
			n	Prevalence (%)	Adjustment[8]	Prevalence (%)	Unadjusted	IRC – CRP + AGP	*n*	Prevalence (%)	Adjustment[8]	*n*	Unadjusted	IRC – AGP	Prevalence (%)	Prevalence (%)
ZAF-12	South Africa (2012)	16–35	1223	15.3	Not specified	–	–	–	–	–	–	–	–	–	9.8 (8.8, 10.9)	–
ZWE-13	Zimbabwe (2013)	15–49	–	–	–	–	–	–	891	61.8	Not specified	–	–	–	8.6 (7.3, 10.1)	–

Abbreviations: AGP, α 1-acid-glycoprotein; BRINDA, Biomarkers Reflecting Inflammation and Nutritional Determinants of Anemia; CRP, C-reactive protein; df, degrees of freedom; GBD, Global Burden of Disease Study; IRC, Internal Regression Correction; QE, Test for Residual Heterogeneity; sTfR, soluble transferrin receptor; VMNIS, Vitamin Mineral Nutrition Information System by the World Health Organization; WRA, women of reproductive age; –, Not Available

[1] Prevalence values are either % or % (95% CI).
[2] Reported from the 'Date' column of the VMNIS database or obtained from selected BRINDA Project Publications, ranging from 2000–2019; Surveys that are reported from selected BRINDA Project Publications have a corresponding 'Before BRINDA Adjustment' and 'After BRINDA Adjustment' sample size and prevalence.
[3] Namaste *et al.* (2020): Depleted iron stores were defined as a ferritin concentration <15 µg/L in WRA. Iron-deficient erythropoiesis was defined as a sTfR concentration >8.3 mg/L in WRA. Updated analysis including additional surveys.
[4] Bangladesh (2011–2012) survey reported prevalence for 2011; Additional reported prevalence (2012): 18.1 (15.2, 21.4).
[5] Namaste *et al.* (2017): Depleted iron stores were defined as a ferritin concentration <15 µg/L in WRA.
[6] Rohner *et al.* (2017): Elevated sTfR defined as >8.3 mg/L.
[7] United States of America (2003–2006) survey reported prevalence for 2006; Additional reported prevalence (2003): 4.9 (4.2, 5.6), (2004): 4.8 (4.0, 5.5), and (2005): 4.8 (4.0, 5.5).
[8] Adjustment for inflammation summarized based on information under 'Indicator Comments' in the VMNIS database. Categorical adjustments refer to the method using internal correction factor(s) suggested by Thurnham et al. (2005; 2010) and BRINDA refers to adjustments using linear regression proposed by the BRINDA project (Namaste et al., 2017).
[9] Dietary iron deficiency among women of reproductive age estimated in the GBD 2019 Study (GBD 2019 Diseases and Injuries Collaborators) as a cause of anemia, as modeled based on hemoglobin concentration in the GBD 2019 Study. These estimates represent only dietary iron deficiency associated with anemia and do not include iron deficiency without anemia.
[10] For Cameroon (2009), the prevalence of iron inadequacy was estimated based on absorbable iron predicted by Armah et al algorithm (7); in Kenya (2011), 8% bioavailability was assumed for iron. In Mexico (2012), the prevalence of inadequate iron intake was estimated with the use of the full-probability approached and assuming 18% iron bioavailability (8).

Supplemental Table S9. Prevalence estimates of vitamin A deficiency or inadequate dietary vitamin A intake among young children in countries with nationally representative survey results[1]

Country survey code	Survey (Year)[2]	Age Range (mo)	Retinol						RBP						GBD 2019 Study[11]	Dietary vitamin A inadequacy
			VMNIS			BRINDA			VMNIS			BRINDA				
			n	Prevalence (%)	Adjustment[10]	*n*	Prevalence, unadjusted (%)	Prevalence adjusted IRC – CRP + AGP (%)	*n*	Prevalence (%)	Adjustment[10]	*n*	Prevalence, unadjusted (%)	Prevalence adjusted IRC – CRP + AGP (%)	Prevalence (%)	Prevalence (%)
AFG-13	Afghanistan (2013)	6–59	–	50	Categorical for elevated CRP and AGP	657[3, 4]	47.6 (42.1, 53.1)[3]; 47.6 (42.2, 53.1)[4]	37.4 (32.2, 42.7)[3]; 37.9 (32.5, 43.7)[4]	–	–	–	–	–	–	64.1 (50.0, 76.2)	–
ARG-05	Argentina (2005)	24–60	–	14	Not specified	–	–	–	–	–	–	–	–	–	16.7 (10.7, 24.7)	–
AZE-13	Azerbaijan (2013)	6–59	–	–	–	–	–	–	1075	8	Categorical for elevated CRP and AGP	1053[3, 4]	12.2 (8.9, 15.5)[3]; 12.2 (9.3, 15.8)[4]	6.1 (3.6, 8.6)[3]; 6.2 (4.2, 9.0)[4]	4.7 (2.6, 7.9)	–
BDI-05	Burundi (2005)	6–59	587	28	Samples with AGP >1 g/L excluded	–	–	–	–	–	–	–	–	–	39.8 (28.2, 52.3)	–
BFA-10	Burkina Faso (2010)	6–59	–	–	–	–	–	–	–	–	–	125[4]	8.9 (4.5, 17.0)[4]	7.3 (2.9, 17.3)[4]	42.7 (30.7, 55.0)	–
BGD-11-12	Bangladesh (2011–2012)	6–59	873	21	Categorical for elevated CRP and AGP	458[3, 4]	20.6 (14.1, 27.1)[3]; 20.6 (14.9, 27.9)[4]	4.7 (2.0, 7.3)[3]; 14.0 (9.0, 21.0)[4]	–	–	–	–	–	–	14.1 (9.1, 20.7)[6]	–
BLZ-11	Belize (2011)	6–59	971	1	Unadjusted	–	–	–	–	–	–	–	–	–	7.9 (4.3, 13.2)	–
BRA-06	Brazil (2006)	6–59	3499	17	Not specified	–	–	–	–	–	–	–	–	–	18.8 (12.5, 26.4)	–
CHN-00	China (2000)	0–72	7826	12	Not specified	–	–	–	–	–	–	–	–	–	11.0 (9.7, 12.6)	–
CIV-07	Côte d'Ivoire (2007)	6–59	–	–	–	–	–	–	782	24	Categorical for elevated CRP and AGP	733[3, 5]; 746[4]	24.0 (20.3, 27.8)[3, 5]; 24.0 (20.4, 27.9)[4]	2.7 (1.4, 4.0)[3]; 3.1 (2.0, 4.7)[4]; 3.1 (1.8, 4.5)[5]	42.5 (28.8, 56.5)	–
CMR-00	Cameroon (2000)	12–71	2375	39	Not specified	–	–	–	–	–	–	–	–	–	43.3 (36.4, 50.4)	59 ± 2
CMR-09	Cameroon (2009)	12–59; 6–59	–	–	–	–	–	–	838	35	Categorical for elevated CRP and AGP	774[3, 5]; 792[4]	28.5 (24.3, 32.7)[3, 5]; 28.7 (24.6, 33.1)[4]	9.3 (7.0, 11.7)[3]; 9.2 (7.1, 11.8)[4]; 8.7 (6.4, 11.0)[5]	34.4 (25.5, 45.2)	–

Supplemental Table S9, continued

Country survey code	Survey (Year)[2]	Age Range (mo)	Retinol						RBP						GBD 2019 Study[11]	Dietary vitamin A inadequacy
			VMNIS			BRINDA			VMNIS			BRINDA				
			n	Prevalence (%)	Adjustment[10]	*n*	Prevalence, unadjusted (%)	Prevalence adjusted IRC – CRP + AGP (%)	*n*	Prevalence (%)	Adjustment[10]	*n*	Prevalence, unadjusted (%)	Prevalence adjusted IRC – CRP + AGP (%)	Prevalence (%)	Prevalence (%)
COL-05	Colombia (2005)	12–48	4409	6	Not specified	–	–	–	–	–	–	–	–	–	6.4 (4.9, 8.2)	–
COL-10	Colombia (2010)	12–48	4394	24	Not specified	3794[3]	23.9 (21.9, 25.8)[3]	17.6 (15.9, 19.3)[3]	–	–	–	–	–	–	5.1 (3.5, 7.0)	–
CRI-09	Costa Rica (2009)	12–72	377	3	Not specified	–	–	–	–	–	–	–	–	–	4.9 (2.9, 7.9)	–
DOM-09	Dominican Republic (2009)	6–59	330	12	Samples with AGP >1 g/L excluded	–	–	–	–	–	–	–	–	–	9.0 (5.3, 14.5)	–
ECU-12	Ecuador (2012)	6–59	2044	18	Unadjusted, also presented w/o inflammation	2017[3]	22.4 (19.4, 25.4)[3]	15.9 (12.8, 19.0)[3]	–	–	–	–	–	–	9.3 (5.4, 15.0)	–
ETH-06	Ethiopia (2006)	6–71	996	38	Not specified	–	–	–	–	–	–	–	–	–	63.9 (51.8, 74.9)	–
ETH-15	Ethiopia (2015)	6–59	1148	14	Categorical for elevated CRP and AGP	–	–	–	–	–	–	–	–	–	42.2 (29.3, 57.4)	–
GBR-12	United Kingdom of Great Britain and Northern Ireland (2012)	12–36	33	0	Not specified	–	–	–	–	–	–	–	–	–	2.0 (1.1, 3.4)	–
GHA-17	Ghana (2017)	6–59	–	–	–	–	–	–	1165	21	Categorical for elevated CRP and AGP	–	–	–	28.3 (17.3, 40.7)	–
GMB-18	Gambia (2018)	6–59	–	–	–	–	–	–	1012	18	BRINDA for CRP and AGP	–	–	–	35.6 (23.6, 49.0)	–

Supplemental Table S9, continued

Country survey code	Survey (Year)[2]	Age Range (mo)	Retinol						RBP						GBD 2019 Study[11]	Dietary vitamin A inadequacy
			VMNIS			BRINDA			VMNIS			BRINDA				
			n	Prevalence (%)	Adjustment[10]	*n*	Prevalence, unadjusted (%)	Prevalence adjusted IRC − CRP + AGP (%)	*n*	Prevalence (%)	Adjustment[10]	*n*	Prevalence, unadjusted (%)	Prevalence adjusted IRC − CRP + AGP (%)	Prevalence (%)	Prevalence (%)
GTM-10	Guatemala (2010)	6–59	1198	0	Unadjusted, also presented w/o inflammation	–	–	–	–	–	–	–	–	–	11.8 (6.9, 19.1)	–
GTM-13	Guatemala (2013)	6–59	–	–	–	–	–	–	858	3	Categorical for elevated CRP and AGP	–	–	–	10.5 (6.2, 17.4)	–
GTM-15	Guatemala (2015)	6–59	–	–	–	–	–	–	682	1	BRINDA for CRP and AGP	–	–	–	10.0 (5.8, 16.5)	–
GUY-12	Guyana (2012)	–59	212	11	Not specified	–	–	–	–	–	–	–	–	–	8.4 (4.8, 13.9)	–
HTI-05	Haiti (2005)	6–59	780	32	Not specified	–	–	–	–	–	–	–	–	–	29.2 (24.6, 34.6)	–
IND-18	India (2018)	12–48	6694	18	Samples with CRP >5 mg/L excluded	–	–	–	–	–	–	–	–	–	18.3 (11.3, 28.4)	–
IRQ-12	Iraq (2012)	12–59	2024	15	Not specified	–	–	–	–	–	–	–	–	–	8.9 (4.9, 14.9)	–
JOR-02	Jordan (2002)	12–59	1036	15	Not specified	–	–	–	–	–	–	–	–	–	15.0 (13.1, 17.2)	–
JOR-10	Jordan (2010)	12–59	915	18	Unadjusted	–	–	–	–	–	–	–	–	–	11.2 (7.6, 15.9)	–
KEN-11	Kenya (2011)	6–59	–	–	–	–	–	–	918	9	Categorical for elevated CRP and AGP	–	–	–	58.4 (45.3, 70.4)	48
KGZ-09	Kyrgyzstan (2009)	6–59	–	–	–	–	–	–	1413	2	Samples with elevated CRP and/or AGP excluded	–	–	–	8.9 (4.9, 14.8)	–
KGZ-13	Kyrgyzstan (2013)	6–29	–	–	–	–	–	–	2148	4	BRINDA for CRP and AGP	–	–	–	7.3 (4.0, 12.2)	–
KHM-14	Cambodia (2014)[7]	6–71; 6–59	–	–	–	–	–	–	775	9	Not specified	665[3, 4]	9.9 (6.5, 13.3)[3]; 9.9 (7.0, 13.8)[4]	6.5 (4.5, 9.5)[4]	22.5 (13.9, 32.7)	–

Supplemental Table S9, continued

Country survey code	Survey (Year)[2]	Age Range (mo)	Retinol						RBP						GBD 2019 Study[11]	Dietary vitamin A inadequacy
			VMNIS			BRINDA			VMNIS			BRINDA				
			n	Prevalence (%)	Adjustment[10]	*n*	Prevalence, unadjusted (%)	Prevalence adjusted IRC – CRP + AGP (%)	*n*	Prevalence (%)	Adjustment[10]	*n*	Prevalence, unadjusted (%)	Prevalence adjusted IRC – CRP + AGP (%)	Prevalence (%)	Prevalence (%)
LAO-00	Lao People's Democratic Republic (2000)	6–59	419	45	Not specified	–	–	–	–	–	–	–	–	–	45.0 (38.8, 51.5)	–
LBR-11	Liberia (2011)	6–35; 6–59	–	–	–	–	–	–	1416	13	Categorical for elevated CRP and AGP	1434[3, 4, 5]	24.7 (21.2, 28.1)[3, 5]; 24.7 (21.4, 28.3)[4]	5.3 (3.8, 6.8)[3]; 5.4 (4.1, 7.1)[4]; 5.4 (3.9, 6.9)[5]	31.2 (20.7, 43.1)	–
LKA-06	Sri Lanka (2006)	6–60	768	29	Not specified	–	–	–	–	–	–	–	–	–	16.7 (11.1, 24.4)	–
MDG-00	Madagascar (2000)	6–59	589	42	Not specified	–	–	–	–	–	–	–	–	–	56.3 (43.1, 68.1)	–
MDV-08	Maldives (2008)	0–60	1247	5	Unadjusted	–	–	–	–	–	–	–	–	–	10.9 (6.5, 16.8)	–
MEX-12	Mexico (2012)	12–59	2593	16	Unadjusted	2512[3]	15.9 (13.4, 18.4)[3]	7.3 (5.7, 8.9)[3]	–	–	–	–	–	–	16.5 (12.3, 21.7)	8.0 ± 2.4
MNG-06	Mongolia (2006)	6–59	–	–	–	202[3]	36.1 (29.5, 42.8)[3, 9]	26.2 (20.3, 32.3)[3, 9]	–	–	–	–	–	–	5.3 (3.6, 7.3)	–
MNG-10	Mongolia (2010)	6–59	–	–	–	–	–	–	433	32	Samples with elevated CRP excluded	–	–	–	3.6 (2.2, 5.4)	–
MOZ-02	Mozambique (2002)	6–59	705	69	Not specified	–	–	–	–	–	–	–	–	–	69.4 (59.5, 78.3)	–
MWI-01	Malawi (2001)	6–36	476	59	Not specified	–	–	–	–	–	–	–	–	–	60.7 (52.9, 68.1)	–
MWI-09	Malawi (2009)	6–59	–	–	–	–	–	–	981	22	Unadjusted	–	–	–	44.8 (34.3, 55.5)	–
MWI-16	Malawi (2016)	6–59	–	–	–	–	–	–	1102	4	Unadjusted	1084[3]; 1102[4]	23.0 (19.1, 26.8)[3, 8]; 24.1 (20.2, 28.5)[4]	7.7 (5.1, 10.2)[3, 8]; 8.1 (5.8, 11.2)[4]	34.4 (22.8, 47.3)	–
NGA-01	Nigeria (2001)	0–59	3099	30	Not specified	–	–	–	–	–	–	–	–	–	30.7 (25.5, 36.4)	–
NGA-05	Nigeria (2005)	6–59	–	–	–	1420[3]	1.9 (1.0, 2.7)[3]	0.8 (0.3, 1.3)[3]	–	–	–	–	–	–	26.5 (20.5, 33.8)	–
NGA-12	Nigeria (2012)	6–59	–	–	–	–	–	–	–	–	–	547[4]	25.6 (20.9, 30.9)[4]	11.5 (8.5, 15.4)[4]	12.3 (7.8, 18.2)	–

Supplemental Table S9, continued

Country survey code	Survey (Year)[2]	Age Range (mo)	Retinol						RBP						GBD 2019 Study[11]	Dietary vitamin A inadequacy
			VMNIS			BRINDA			VMNIS			BRINDA				
			n	Prevalence (%)	Adjustment[10]	*n*	Prevalence, unadjusted (%)	Prevalence adjusted IRC − CRP + AGP (%)	*n*	Prevalence (%)	Adjustment[10]	*n*	Prevalence, unadjusted (%)	Prevalence adjusted IRC − CRP + AGP (%)	Prevalence (%)	Prevalence (%)
NIC-00	Nicaragua (2000)	6–59	2381	9	Unadjusted, also presented w/o inflammation	–	–	–	–	–	–	–	–	–	7.7 (6.9, 8.4)	–
NIC-03	Nicaragua (2003)	6–59	470	0	Not specified	–	–	–	–	–	–	–	–	–	3.9 (3.4, 4.4)	–
NIC-04	Nicaragua (2004)	6–59	344	1	Samples with AGP >1 g/L excluded	–	–	–	–	–	–	–	–	–	3.1 (2.7, 3.6)	–
NIC-05	Nicaragua (2005)	6–59	365	2	Samples with AGP >1 g/L excluded	–	–	–	–	–	–	–	–	–	2.7 (2.3, 3.2)	–
NIC-07	Nicaragua (2007)	6–59	–	10	Not specified	–	–	–	–	–	–	–	–	–	2.1 (1.6, 2.6)	–
NPL-16	Nepal (2016)	6–59	657	11	BRINDA using CRP and AGP	–	–	–	1651	3	BRINDA for CRP and AGP	–	–	–	11.5 (7.0, 17.3)	–
OMN-04	Oman (2004)	6–59	152	6	Samples with CRP >10 mg/L excluded	–	–	–	–	–	–	–	–	–	5.4 (3.1, 8.8)	–
PAK-01	Pakistan (2001)	6–59	5682	13	Not specified	–	–	–	–	–	–	–	–	–	16.2 (13.7, 19.2)	–
PAK-11	Pakistan (2011)[7]	6–59	–	–	–	7318[3]	52.3 (50.1, 54.6)[3]	–	–	–	–	–	–	–	9.0 (5.8, 13.6)	–
PER-00	Peru (2000)	0–48	657	13	Not specified	–	–	–	–	–	–	–	–	–	18.0 (12.6, 24.8)	–
PER-01	Peru (2001)	0–48	734	15	Not specified	–	–	–	–	–	–	–	–	–	17.6 (12.1, 24.6)	–
PHL-03	Philippines (2003)	6–59	3544	40	Not specified	–	–	–	–	–	–	–	–	–	38.0 (31.7, 45.7)	–
PHL-08	Philippines (2008)	0–60	2408	15	Not specified	–	–	–	–	–	–	–	–	–	39.6 (30.0, 50.2)	–
PHL-14	Philippines (2014)	6–71	3139	20	Not specified	–	–	–	–	–	–	–	–	–	31.0 (21.1, 43.3)	–

Country survey code	Survey (Year)[2]	Age Range (mo)	Retinol						RBP						GBD 2019 Study[11]	Dietary vitamin A inadequacy
			VMNIS			BRINDA			VMNIS			BRINDA				
			n	Prevalence (%)	Adjustment[10]	*n*	Prevalence, unadjusted (%)	Prevalence adjusted IRC − CRP + AGP (%)	*n*	Prevalence (%)	Adjustment[10]	*n*	Prevalence, unadjusted (%)	Prevalence adjusted IRC − CRP + AGP (%)	Prevalence (%)	Prevalence (%)
PNG-05	Papua New Guinea (2005)	6–59	–	–	–	–	–	–	875	26	Unadjusted, also presented w/o inflammation	871[3, 5]; 868[4]	25.0 (20.8, 29.3)[3, 5]; 24.7 (20.7, 29.2)[4]	9.9 (7.7, 12.0)[3]; 9.8 (7.8, 12.3)[4]; 9.1 (7.1, 11.2)[5]	26.7 (17.3, 37.1)	–
RWA-10	Rwanda (2010)	6–59	–	–	–	–	–	–	–	–	–	576[4]	10.7 (8.1, 14.0)[4]	4.5 (2.9, 6.9)[4]	35.2 (22.8, 49.0)	–
SEN-10	Senegal (2010)	12–59	1418	18	Categorical for elevated CRP and AGP	–	–	–	–	–	–	–	–	–	21.3 (12.5, 32.4)	–
SLE-13	Sierra Leone (2013)	6–59	–	–	–	–	–	–	654	17	Categorical for elevated CRP and AGP	–	–	–	48.8 (34.7, 62.3)	–
TLS-13	Timor-Leste (2013)	6–59	546	8	Categorical for elevated CRP and AGP	–	–	–	547	10	Categorical for elevated CRP and AGP	–	–	–	27.5 (16.8, 40.3)	–
TZA-10	United Republic of Tanzania (2010)	6–59	–	–	–	–	–	–	6294	38	Unadjusted, also presented categorical for elevated CRP	–	–	–	32.4 (27.4, 37.8)	–
UGA-01	Uganda (2001)	6–59	859	28	Not specified	–	–	–	–	–	–	–	–	–	29.6 (25.2, 34.6)	–
UGA-06	Uganda (2006)	6–59	–	–	–	–	–	–	2460	20	Not specified	–	–	–	27.5 (23.5, 32.1)	–
UGA-11	Uganda (2011)	6–59	–	–	–	–	–	–	2091	38	Unadjusted, also presented categorical for elevated CRP	–	–	–	30.7 (26.0, 35.8)	–
UGA-16	Uganda (2016)	6–59	–	–	–	–	–	–	4694	9	Categorical for elevated CRP	–	–	–	27.2 (20.5, 34.8)	–
UZB-17	Uzbekistan (2017)	6–59	1665	6	BRINDA for CRP and AGP	–	–	–	–	–	–	–	–	–	5.0 (2.7, 8.4)	–

Supplemental Table S9, continued

Country survey code	Survey (Year)[2]	Age Range (mo)	Retinol						RBP						GBD 2019 Study[11]	Dietary vitamin A inadequacy
			VMNIS			BRINDA			VMNIS			BRINDA				
			n	Prevalence (%)	Adjustment[10]	*n*	Prevalence, unadjusted (%)	Prevalence adjusted IRC – CRP + AGP (%)	*n*	Prevalence (%)	Adjustment[10]	*n*	Prevalence, unadjusted (%)	Prevalence adjusted IRC – CRP + AGP (%)	Prevalence (%)	Prevalence (%)
VNM-10	Viet Nam (2010)	10–75	546	10	Categorical for elevated CRP	360[3]	6.9 (4.3, 9.6)[3]	5.6 (3.1, 8.0)[3]	–	–	–	–	–	–	18.8 (14.0, 24.4)	–
ZAF-05	South Africa (2005)	12–71	833	65	Categorical for elevated CRP	–	–	–	–	–	–	–	–	–	18.8 (14.2, 24.4)	–
ZAF-12	South Africa (2012)	0–59	438	44	Unadjusted	–	–	–	–	–	–	–	–	–	13.5 (8.6, 20.0)	–
ZMB-03	Zambia (2003)	6–59	659	54	Unadjusted	–	–	–	–	–	–	–	–	–	56.0 (44.7, 66.9)	–
ZMB-09	Zambia (2009)	6–59	–	–	–	389[4]	56.6 (49.4, 63.4)[4]	43.7 (36.1, 51.6)[4]	–	–	–	–	–	–	43.1 (30.4, 55.3)	–
ZWE-13	Zimbabwe (2013)	6–59	–	–	–	–	–	–	1727	21	Categorical for elevated CRP	–	–	–	40.5 (28.2, 53.7)	–

Abbreviations: AGP, α 1-acid-glycoprotein; BRINDA, Biomarkers Reflecting Inflammation and Nutritional Determinants of Anemia; CRP, C-reactive protein; GBD, Global Burden of Disease Study; IRC, Internal Regression Correction; RBP, retinol binding protein; VMNIS, Vitamin Mineral Nutrition Information System by the World Health Organization; –, Not Available

[1] Prevalence values are either % or % (95% CI).
[2] Reported from the 'Date' column of the VMNIS database or obtained from selected BRINDA Project Publications, ranging from 2000–2019; Surveys that are reported from selected BRINDA Project Publications have a corresponding 'Before BRINDA Adjustment' and 'After BRINDA Adjustment' sample size and prevalence.
[3] Larson *et al.* (2018): Vitamin A deficiency is defined as either RBP or Retinol concentrations <0.70 μmol/L in PSC.
[4] Namaste *et al.* (2020): Vitamin A deficiency is defined as either RBP or Retinol concentrations <0.70 μmol/L in PSC. Updated analysis including additional surveys.
[5] Larson *et al.* (2017): Vitamin A deficiency is defined as an RBP concentration <0.70 μmol/L.
[6] Bangladesh (2011–2012) survey reported prevalence for 2011; Additional reported prevalence (2012): 13.3 (8.4, 19.7).
[7] Larson *et al.* (2018): No BRINDA adjustments were made for Pakistan (2011) and Cambodia (2014) surveys, because of poor correlation between vitamin A measures and inflammation.
[8] Larson *et al.* (2018): Vitamin A deficiency among the subset of samples with non-elevated inflammatory biomarkers.
[9] Larson *et al.* (2018): Mongolia did not apply complex survey design, so a binomial proportion test was used to calculate 95% CI.
[10] Adjustment for inflammation summarized based on information under 'Indicator Comments' in the VMNIS database. Categorical adjustments refer to the method using internal correction factor(s) suggested by Thurnham et al. (2005; 2010) and BRINDA refers to adjustments using linear regression proposed by the Biomarkers Reflecting Inflammation and Nutritional Determinants of Anemia (BRINDA) project (Larson et al., 2017); Namaste et al, 2020).
[11] The primary source of vitamin A deficiency data for the GBD 2019 Study were from the WHO VMNIS database. The GBD 2019 Study used Spatiotemporal Gaussian Process Regression (ST-GPR) model to estimate for each year and location (GBD 2019 Risk Factor Collaborators).

Supplemental Table S10. Prevalence estimates of vitamin A deficiency or inadequate dietary vitamin A intake among women of reproductive age in countries with nationally representative survey results[1]

Country survey code	Survey (Year)[2]	Age Range (yr)	Retinol					RBP					GBD 2019 Study[8]	Dietary vitamin A inadequacy
			VMNIS			BRINDA		VMNIS						
			n	Prevalence (%)	Adjustment[7]	Prevalence (%)	Prevalence, unadjusted (%)	*n*	Prevalence (%)	Adjustment[10]	*n*	Prevalence, unadjusted (%)	Prevalence (%)	Prevalence (%)
AFG-13	Afghanistan (2013)	15–49	–	11	Categorical for elevated CRP and AGP	–	10.1 (7.5, 12.7)[3]	–	–	–	–	–	24.5 (20.1, 29.3)	
AZE-13	Azerbaijan (2013)	15–49	–	–	–	–	–	–	1	Categorical for elevated CRP and AGP	2656[3]	0.4 (0.1, 0.7)[3]	2.8 (2.2, 3.5)	
BGD-11-12	Bangladesh (2011–2012)	15–49	918	5	Categorical for elevated CRP and AGP	–	7.2 (3.7, 10.7)[3]	–	–	–	–	–	6.7 (5.4, 8.2)[4]	
BLZ-11	Belize (2011)	15–49	937	1	Unadjusted	–	–	–	–	–	–	–	5.5 (4.4, 6.9)	
BRA-06	Brazil (2006)	15–49	5698	12	Not specified	–	–	–	–	–	–	–	14.8 (12.0, 18.0)	
CIV-07	Côte d'Ivoire (2007)	15–49	–	–	–	–	–	907	1	Categorical for elevated CRP and AGP	816[3]	0.7 (0.2, 1.3)[3]	15.3 (12.5, 18.6)	
CMR-09	Cameroon (2009)	15–49	–	–	–	–	6.8 (1.6, 12.0)[3]	872	3	Categorical for elevated CRP and AGP	751[3]	1.5 (0.6, 2.4)[3]	35.5 (30.8, 40.2)	
ECU-12	Ecuador (2012)	15–49	–	–	–	5979[3]	2.6 (1.5, 3.7)[3]	–	–	–	–	–	3.9 (3.0, 5.0)	
ETH-15	Ethiopia (2015)	15–49	1619	3	Categorical for elevated CRP and AGP	–	–	–	–	–	–	–	28.8 (24.0, 34.0)	
FJI-04	Fiji (2004)	15–44	731	3	Not specified	–	–	–	–	–	–	–	9.0 (6.6, 11.8)	
GBR-01	United Kingdom of Great Britain and Northern Ireland (2001)	19–64	616	0	Not specified	–	–	–	–	–	–	–	0.5 (0.4, 0.7)	
GBR-12	United Kingdom of Great Britain and Northern Ireland (2012)	19–64	756	1	Not specified	–	–	–	–	–	–	–	0.5 (0.4, 0.6)	

Country survey code	Survey (Year)[2]	Age Range (yr)	Retinol					RBP					GBD 2019 Study[8]	Dietary vitamin A inade-quacy
			VMNIS			BRINDA		VMNIS						
			n	Prevalence (%)	Adjustment[7]	Prevalence (%)	Prevalence, unadjusted (%)	*n*	Prevalence (%)	Adjustment[10]	*n*	Prevalence, unadjusted (%)	Prevalence (%)	Prevalence (%)
GBR-14	United Kingdom of Great Britain and Northern Ireland (2014)	15–49	–	–	–	875[3]	1.0 (0, 2.1)[3]	–	–	–	–	–	0.4 (0.3, 0.5)	
GHA-17	Ghana (2017)	15–49	–	–	–	–	–	987	2	Unadjusted	–	–	13.9 (11.0, 17.4)	
GMB-18	Gambia (2018)	15–49	–	–	–	–	–	139 1	2	Unadjusted	–	–	19.5 (15.6, 23.8)	
GTM-13	Guatemala (2013)	15–49	–	–	–	–	–	162 1	0	Categorical for elevated CRP and AGP	–	–	6.6 (5.2, 8.3)	
GTM-15	Guatemala (2015)	15–49	–	–	–	–	–	151 5	0	Unadjusted	–	–	6.1 (4.9, 7.7)	
IRQ-12	Iraq (2012)	15–49	1066	4	Not specified	–	–	–	–	–	–	–	4.5 (3.5, 5.8)	
JOR-10	Jordan (2010)	15–49	2032	5	Unadjusted	–	–	–	–	–	–	–	6.3 (5.0, 7.8)	
KEN-11	Kenya (2011)	15–49	–	–	–	–	–	632	1	Categorical for elevated CRP and AGP	–	–	34.7 (29.9, 39.6)	
KGZ-09	Kyrgyzstan (2009)	17–	–	–	–	–	–	102 6	1	Samples with elevated CRP and/or AGP excluded	–	–	5.0 (4.0, 6.4)	
KHM-14	Cambodia (2014)[5]	15–49	–	–	–	–	–	739	3	Not specified	705[3]	2.9 (1.6, 4.1)[3]	6.9 (5.4, 8.9)	
LAO-00	Lao People's Democratic Republic (2000)	19–25	172	22	Not specified	–	–	–	–	–	–	–	24.5 (22.1, 26.8)	
LBR-11	Liberia (2011)	15–49	–	–	–	–	–	191 1	2	Categorical for elevated CRP and AGP	1875[3]	2.3 (1.4, 3.1)[3]	16.0 (12.5, 19.7)	
LKA-06	Sri Lanka (2006)	–49	290	15	Not specified	–	–	–	–	–	–	–	5.8 (4.6, 7.1)	
MDV-08	Maldives (2008)	15–49	1297	5	Unadjusted	–	–	–	–	–	–	–	4.3 (3.2, 5.6)	
MOZ-02	Mozambique (2002)	Not specified	557	11	Not specified	–	–	–	–	–	–	–	46.1 (40.8, 51.2)	

Supplemental Table S10, continued

Country survey code	Survey (Year)[2]	Age Range (yr)	Retinol					RBP					GBD 2019 Study[8]	Dietary vitamin A inade-quacy
			VMNIS			BRINDA		VMNIS						
			n	Prevalence (%)	Adjustment[7]	Prevalence (%)	Prevalence, unadjusted (%)	*n*	Prevalence (%)	Adjustment[10]	*n*	Prevalence, unadjusted (%)	Prevalence (%)	Prevalence (%)
MWI-01	Malawi (2001)	15–45	464	57	Not specified	–	–	–	–	–	–	–	47.9 (43.7, 52.5)	
MWI-09	Malawi (2009)	15–49	–	–	–	–	–	509	2	Unadjusted	–	–	37.6 (33.2, 42.7)	
MWI-16	Malawi (2016)	15–49	–	–	–	–	–	752	0	Unadjusted	753[3]	3.0 (1.3, 4.7)[3, 6]	29.5 (25.1, 34.3)	
NGA-01	Nigeria (2001)	Not specified	3148	4	Unadjusted	–	–	–	–	–	–	–	13.8 (11.2, 16.7)	
NIC-00	Nicaragua (2000)	Not specified	2221	1	Unadjusted	–	–	–	–	–	–	–	4.9 (3.8, 6.3)	
NPL-16	Nepal (2016)	15–49	527	5	BRINDA for CRP and AGP	–	–	2129	1	BRINDA for CRP and AGP	–	–	5.9 (4.8, 7.2)	
OMN-04	Oman (2004)	15–49	341	0	Samples with CRP >10 mg/L excluded	–	–	–	–	–	–	–	2.5 (1.9, 3.3)	
PAK-01	Pakistan (2001)	15–49	484	5	Not specified	–	–	–	–	–	–	–	6.4 (5.1, 7.9)	
PAK-11	Pakistan (2011)[5]	15–49	–	–	–	5929[3]	39.1 (36.8, 41.4)[3]	–	–	–	–	–	4.1 (3.3, 5.1)	
PER-00	Peru (2000)	15–49	968	10	Not specified	–	–	–	–	–	–	–	11.0 (9.2, 13.2)	
PER-01	Peru (2001)	15–49	1228	9	Not specified	–	–	–	–	–	–	–	10.8 (9.0, 13.0)	
PNG-05	Papua New Guinea (2005)	15–49	–	–	–	–	–	–	–	–	749[3]	0.6 (0, 1.1)[3]	14.4 (11.5, 17.6)	
SEN-10	Senegal (2010)	15–49	983	2	Categorical for elevated CRP and AGP	–	–	–	–	–	–	–	17.4 (14.0, 21.3)	
SLE-13	Sierra Leone (2013)	15–49	–	–	–	–	–	817	2	Categorical for elevated CRP and AGP	–	–	22.0 (17.8, 26.5)	
TLS-13	Timor-Leste (2013)	14–60	616	3	Categorical for elevated CRP and AGP	–	–	592	5	Categorical for elevated CRP and AGP	–	–	5.2 (3.9, 6.9)	

Country survey code	Survey (Year)[2]	Age Range (yr)	Retinol					RBP					GBD 2019 Study[8]	Dietary vitamin A inade-quacy
			VMNIS			BRINDA		VMNIS						
			n	Prevalence (%)	Adjustment[7]	Prevalence (%)	Prevalence, unadjusted (%)	*n*	Prevalence (%)	Adjustment[10]	*n*	Prevalence, unadjusted (%)	Prevalence (%)	Prevalence (%)
TZA-10	United Republic of Tanzania (2010)	15–49	–	–	–	–	–	570 7	42	Unadjusted, also presented categorical for elevated CRP	–	–	18.4 (15.3, 22.0)	
UGA-01	Uganda (2001)	15–49	461	22	Not specified	–	–	–	–	–	–	–	25.5 (21.8, 29.4)	
UGA-06	Uganda (2006)	15–49	–	–	–	–	–	154 2	21	Not specified	–	–	18.7 (15.6, 22.0)	
UGA-11	Uganda (2011)	15–49	–	–	–	–	–	154 7	37	Unadjusted, also presented categorical for elevated CRP	–	–	15.2 (12.4, 18.2)	
USA-06	United States of America (2006)	15–49	–	–	–	3145[3]	0.3 (0.1, 0.6)[3]	–	–	–	–	–	0.4 (0.3, 0.5)	
UZB-17	Uzbekistan (2017)	15–49	1990	3	BRINDA for CRP and AGP	–	–	–	–	–	–	–	2.9 (2.3, 3.6)	
VNM-10	Viet Nam (2010)	15–49	1475	2	Categorical for elevated CRP	1434[3]	1.3 (0.7, 1.9)[3]	–	–	–	–	–	0.8 (0.5, 1.1)	
ZAF-05	South Africa (2005)	16–35	1834	27	Categorical for elevated CRP	–	–	–	–	–	–	–	3.9 (3.0, 4.9)	
ZAF-12	South Africa (2012)	16–35	1158	13	Unadjusted	–	–	–	–	–	–	–	2.8 (2.2, 3.6)	
ZMB-03	Zambia (2003)	15–49	–	13	Unadjusted, also presented categorical for elevated CRP and AGP	–	–	–	–	–	–	–	28.6 (24.1, 32.9)	
ZWE-13	Zimbabwe (2013)	15–49	–	–	–	–	–	838	23	Categorical for elevated CRP	–	–	21.7 (18.3, 25.8)	

Abbreviations: AGP, α 1-acid-glycoprotein; BRINDA, Biomarkers Reflecting Inflammation and Nutritional Determinants of Anemia; CRP, C-reactive protein; GBD, Global Burden of Disease Study; IRC, Internal Regression Correction; RBP, retinol binding protein; VMNIS, Vitamin Mineral Nutrition Information System by the World Health Organization; –, Not Available

[1] Prevalence values are either % or % (95% CI).
[2] Reported from the 'Date' column of the VMNIS database or obtained from selected BRINDA Project Publications, ranging from 2000–2019; Surveys that are reported from selected BRINDA Project Publications have a corresponding 'Before BRINDA Adjustment' and 'After BRINDA Adjustment' sample size and prevalence.
[3] Larson *et al.* (2018): Vitamin A deficiency is defined as either RBP or retinol concentrations <0.70 µmol/L in PSC.

[4] Bangladesh (2011–2012) survey reported prevalence for 2011; Additional reported prevalence (2012): 6.3 (5.1, 7.7).
[5] Larson *et al.* (2018): No BRINDA adjustments were made for Pakistan (2011) and Cambodia (2014) surveys, because of poor correlation between vitamin A measures and inflammation.
[6] Larson *et al.* (2018): Vitamin A deficiency among the subset of samples with non-elevated inflammatory biomarkers.
[7] Adjustment for inflammation summarized based on information under 'Indicator Comments' in the VMNIS database. Categorical adjustments refer to the method using internal correction factor(s) suggested by Thurnham et al. (2005; 2010) and BRINDA refers to adjustments using linear regression proposed by the Biomarkers Reflecting Inflammation and Nutritional Determinants of Anemia (BRINDA) project (Larson et al., 2017); Namaste et al, 2020).
[8] The primary source of vitamin A deficiency data for the GBD 2019 study were from the WHO VMNIS database. GBD 2019 study used Spatiotemporal Gaussian Process Regression (ST-GPR) model to estimate for each year and location (GBD 2019 Risk Factor Collaborators).

Supplemental Table S11. Prevalence estimates of zinc deficiency or inadequate dietary zinc intake among young children in countries with nationally representative survey results[1]

Country survey code	Survey (Year)[2]	Age Range (mo)	Zinc						GBD 2019 Study[6]
			VMNIS			BRINDA			
			n	Prevalence (%)	Adjustment[5]	*n*	Prevalence, unadjusted (%)	Prevalence, adjusted IRC − CRP + AGP (%)	Prevalence (%)
AFG-13	Afghanistan (2013)	6–59	728	15.1	Categorical for elevated CRP and AGP	658[3]	25.5 (20.6, 30.3)[3]	21.4 (17.0, 25.8)[3]	41.6 (31.0, 48.5)
AZE-13	Azerbaijan (2013)	6–59	1040	10.7	Unadjusted	1016[3]	14.0 (11.5, 16.4)[3]	7.0 (5.2, 8.7)[3]	6.7 (0.4, 17.8)
BGD-11-12	Bangladesh (2011–2012)	6–59	662	44.6	Categorical for elevated CRP and AGP	309[3]	–	–	13.2 (2.1, 27.7)[4]
CMR-09	Cameroon (2009)	12–59	680	82.6	Categorical for elevated CRP and AGP	776[3]	80.0 (76.7, 83.3)[3]	61.8 (57.8, 65.8)[3]	5.5 (0.1, 15.5)
COL-05	Colombia (2005)	12–48	3974	26.9	Not specified	–	–	–	8.7 (0.9, 21.5)
COL-10	Colombia (2010)	12–48	4279	43.3	Not specified	3573[3]	–	–	8.6 (0.9, 21.0)
CRI-09	Costa Rica (2009)	12–72	224	23.9	Not specified	–	–	–	6.7 (0.4, 17.9)
ECU-12	Ecuador (2012)	6–59	2045	27.5	Unadjusted, also presented w/o inflammation	2017[3]	27.6 (24.7, 30.4)[3]	–	14.1 (2.7, 29.0)
ETH-15	Ethiopia (2015)	6–59	1143	35	Not specified	–	–	–	3.1 (0.0, 9.5)
GTM-10	Guatemala (2010)	6–59	1196	34.9	Not specified	–	–	–	6.9 (0.4, 18.3)
GTM-13	Guatemala (2013)	6–59	65	40	Not specified	–	–	–	6.7 (0.4, 17.4)
GTM-16	Guatemala (2016)	6–59	63	13.3	Not specified	–	–	–	6.5 (0.4, 17.0)
IND-18	India (2018)	12–48	8662	18.9	Samples with CRP >5 mg/L excluded	–	–	–	12.2 (2.3, 24.9)
KEN-11	Kenya (2011)	6–59	768	83.3	Categorical for elevated CRP and AGP	–	–	–	5.7 (0.2, 15.7)
KHM-14	Cambodia (2014)	6–72	656	67.5	Not specified	534[3]	68.3 (61.0, 75.6)[3]	62.1 (54.2, 70.0)[3]	8.1 (0.6, 20.7)
LKA-12	Sri Lanka (2012)	6–59	4463	5.1	Samples with CRP >5 g/L excluded	–	–	–	8.2 (0.8, 20.7)
MDV-08	Maldives (2008)	0–60	1255	16	Not specified	–	–	–	20.0 (5.6, 34.8)
MEX-06	Mexico (2006)	12–48	1550	28.1	Not specified	1164[3]	–	–	2.5 (0.0, 8.0)
MWI-16	Malawi (2016)	6–59	1086	60.4	Unadjusted	1071[3]	61.3 (55.2, 67.4)[3]	47.2 (40.9, 53.5)[3]	4.9 (0.0, 14.4)
NGA-01	Nigeria (2001)	0–59	2725	20	Not specified	–	–	–	8.9 (1.0, 21.5)
NPL-16	Nepal (2016)	6–59	1647	20.7	BRINDA using CRP and AGP	–	–	–	4.0 (0.0, 12.3)
PAK-01	Pakistan (2001)	6–59	5644	37.1	Not specified	–	–	–	34.6 (24.4, 43.5)
PHL-08	Philippines (2008)	6–59	2370	21.6	Not specified	–	–	–	14.7 (3.9, 28.3)
PHL-14	Philippines (2014)	6–59	3124	17.9	Not specified	–	–	–	12.1 (2.3, 25.8)

Supplemental Table S11, continued

Country survey code	Survey (Year)[2]	Age Range (mo)	Zinc						GBD 2019 Study[6]
			VMNIS			BRINDA			
			n	Prevalence (%)	Adjustment[5]	*n*	Prevalence, unadjusted (%)	Prevalence, adjusted IRC − CRP + AGP (%)	Prevalence (%)
SEN-10	Senegal (2010)	12–59	1151	40.7	Categorical for elevated CRP and AGP	–	–	–	3.4 (0.0, 10.6)
TLS-13	Timor-Leste (2013)	6–59	580	60.3	Not specified	–	–	–	8.0 (0.7, 20.2)
VNM-10	Viet Nam (2010)	10–75	563	51.9	Unadjusted	375[3]	–	–	6.0 (0.2, 16.5)
ZAF-05	South Africa (2005)	12–36	154	51.3	Not specified	–	–	–	4.6 (0.1, 13.1)

Abbreviations: AGP, α 1-acid-glycoprotein; BRINDA, Biomarkers Reflecting Inflammation and Nutritional Determinants of Anemia; CRP, C-reactive protein; GBD, FAO SUA , Food and Agriculture Organization Supply Account Utilization, Global Burden of Disease Study; IRC, Internal Regression Correction; PZC, plasma or serum zinc concentrations; VMNIS, Vitamin Mineral Nutrition Information System by the World Health Organization; –, Not Available

[1] Prevalence values are either % or % (95% CI).
[2] Reported from the 'Date' column of the VMNIS database or obtained from selected BRINDA Project Publications, ranging from 2000–2019; Surveys that are reported from selected BRINDA Project Publications have a corresponding 'Before BRINDA Adjustment' and 'After BRINDA Adjustment' sample size and prevalence.
[3] McDonald *et al*. (2020): Zinc deficiency is defined as a plasma or serum zinc concentration <57 µg/dL in the afternoon or <65 µg/dL in the morning (nonfasting) in PSC.
[4] Bangladesh (2011–2012) survey reported prevalence for 2011; Additional reported prevalence (2012): 12.7 (1.9, 27.0).
[5] Adjustment for inflammation summarized based on information under 'Indicator Comments' in the VMNIS database. Categorical adjustments refer to the method using internal correction factor(s) suggested by Thurnham et al. (2005; 2010) and BRINDA refers to adjustments using linear regression proposed by the Biomarkers Reflecting Inflammation and Nutritional Determinants of Anemia (BRINDA) project (McDonald et al., 2020).
[6] The prevalence of zinc deficiency among children 1- 4 years of age in the GBD 2019 Study is estimated based on dietary intake data from nationally and sub-nationally representative nutrition surveys and from food availability data obtained from FAO SUA (after adjusting for food waste) (GBD 2019 Risk Factor Collaborators). This information was then used to predict the mean zinc intake at the population level, and to characterize the distribution of zinc intake, as a proxy for zinc status. Zinc deficiency was defined as consumption of less than 2.5 mg of zinc per day, which is the Estimated Average Requirement (EAR) for children 1-4 years based on Dietary Reference Intakes of the US Institute of Medicine. GBD 2019 study used Spatiotemporal Gaussian Process Regression (ST-GPR) model to estimate for each year and location (GBD 2019 Risk Factor Collaborators).

Supplemental Table S12. Prevalence estimates of zinc deficiency among women of reproductive age in countries with nationally representative survey results[1]

Country survey code	Survey (Year)[2]	Age Range (yr)	Zinc		
			VMNIS		
			n	Prevalence (%)	Adjustment[4]
AFG-13	Afghanistan (2013)	15–49	1187	23.4	Categorical for elevated CRP and AGP
AUT-12	Austria (2012)	18–64	229	18.1	Not specified
BGD-11-12	Bangladesh (2011–2012)	15–49	1073	57.3	Categorical for elevated CRP and AGP
CMR-09	Cameroon (2009)	15–49	446	83.2	Categorical for elevated CRP and AGP
ECU-12	Ecuador (2012)	12–49	8186	56.1	Unadjusted, also presented w/o inflammation
ETH-15	Ethiopia (2015)	15–49	1625	33.8	Not specified
FJI-04	Fiji (2004)	15–44	717	39.2	Not specified
FJI-10	Fiji (2010)	15–45	869	0	Not specified
GTM-13	Guatemala (2013)	15–49	85	34.1	Not specified
GTM-16	Guatemala (2016)	15–49	130	18.3	Not specified
KEN-11	Kenya (2011)	15–49	653	82.3	Categorical for elevated CRP and AGP
KHM-14	Cambodia (2014)	15–49	720	62.8	Not specified
MDV-08	Maldives (2008)	15–49	1282	26.8	Not specified
MEX-06	Mexico (2006)[3]	30-39	601	30.6	Not specified
MWI-16	Malawi (2016)	15–49	757	62.5	Unadjusted
NGA-01	Nigeria (2001)	Not specified	3779	28.1	Not specified
NPL-16	Nepal (2016)	15–49	2132	24.3	Unadjusted
PAK-01	Pakistan (2001)	15–49	451	40.1	Not specified
PHL-08	Philippines (2008)	20–59	2892	31.2	Not specified
PHL-14	Philippines (2014)	20–59	7139	28.4	Not specified
SEN-10	Senegal (2010)	15–49	983	67.2	Unadjusted
VNM-10	Viet Nam (2010)	15–49	1522	67.2	Unadjusted

Abbreviations: AGP, α 1-acid-glycoprotein; BRINDA, Biomarkers Reflecting Inflammation and Nutritional Determinants of Anemia; CRP, C-reactive protein; GBD, Global Burden of Disease Study; IRC, Internal Regression Correction; VMNIS, Vitamin Mineral Nutrition Information System by the World Health Organization; –, Not Available

[1] Prevalence values are either % or % (95% CI).
[2] Reported from the 'Date' column of the VMNIS database, ranging from 2000–2019.
[3] Reported here is the prevalence of low plasma zinc concentration in 30-39 yr old women. The survey in Mexico 2006, also reported low plasma zinc concentrations for 20-29 yr olds (19.3%) and for 40-49 yr olds (33.1%), respectively.
[4] Adjustment for inflammation summarized based on information under 'Indicator Comments' in the VMNIS database. Categorical adjustments refer to the method using internal correction factor(s) suggested by Thurnham et al. (2005; 2010) and BRINDA refers to adjustments using linear regression proposed by the Biomarkers Reflecting Inflammation and Nutritional Determinants of Anemia (BRINDA) project (McDonald et al., 2020).
