# Supplementary material for: Comparison of Published Estimates of the National Prevalence of Iron, Vitamin A, and Zinc Deficiency and Sources of Inconsistencies
Source: Adv Nutr. 2023 Aug 25;14(6):1466–78. doi: 10.1016/j.advnut.2023.08.011 (PMC10721533; doi:10.1016/j.advnut.2023.08.011)
Supplement: Multimedia component3 [file mmc3.pdf]

Comparison of sources of inconsistency in available estimates of the national prevalence of iron, vitamin A and zinc deficiency

by Hess SY, Wessells KR, Haile D, Rogers LM, Tan X, Barros JG, Bourassa MW, Gorstein J, Brown KH

Supplemental table S13: Prevalence of inadequate iron, zinc and vitamin A intake among young children and women of reproductive age in nationally representative surveys that also measured iron, zinc and vitamin A status by biomarker

Survey	Children					Women					Refs
	N	Age (mo)	Prevalence of inadequate intake (%)			N	Age (yr)	Prevalence of inadequate intake (%)			
			Iron[1]	Zinc[2]	Vitamin A			Iron[1]	Zinc[2]	Vitamin A	
Cameroon (2009)	NBF 677	12 – 59	69.9 ± 1.4	19.1 ± 2.7	59 ± 2	912	15-49	94.9 ± 0.7	54.3 ± 0.9	53 ± 3	(1-4)
Kenya (2011)	NBF &BF, 223	12 – 59	67	17	48	NPNL, 232	19-49	91	34	33	(5)
Mexico (2012)	NBF, 2113	12 – 59	4.8 ± 0.2	1.0 ± 0.9	8.0 ± 2.4	NPNL, 1799	≥ 20	22.2 ± 0.5	10.2 ± 13.6	53.8 ± 1.9	(6, 7)

BF, Breast fed; NBF, non-breastfed; NPNL, non-pregnant and non-lactating

[1] For Cameroon (2009), the prevalence of inadequate iron intake was for all children regardless of breastfeeding status (4). The prevalence of inadequate iron intake was calculated by applying the full probability method to the usual intake distribution estimated according to National Cancer Institute Method. This study assumed only 10% of the total dietary iron intake is heme iron and 25% absorption of heme iron. The absorption proportion of nonheme iron was predicted by Armah et al algorithm (8); In Kenya (2011), 8% bioavailability was assumed for iron (5). The IOM EAR values (8) were used to estimate the prevalence of inadequate intake using EAR cutpoint method. Usual intake distribution was estimated by Iowa State University (ISU) method using PC-SIDE. In Mexico (2012), the prevalence of inadequate iron intake was estimated with the use of the full-probability approach as proposed by the IOM and assuming 18% iron bioavailability (9). If bioavailability was based on the Mexican Dietary Recommendations for traditional diets, bioavailability of 5.5% was assumed for children <4 yr of age which resulted in a prevalence of inadequate iron intake of 52.2 ± 0.6% and iron bioavailability of 7.5% for women which resulted in a prevalence of inadequate iron intake of 89.3 ± 0.4% (6). Usual intake distribution was estimated by Iowa State University (ISU) method using PC-SIDE.

[2] For zinc, data shown for all children regardless of breastfeeding status for Cameroon and Kenya. For Cameroon, absorbable zinc intake of 0.145 mg/day from breast milk was assumed. IOM EAR values were used to estimate the prevalence of zinc inadequate intake in all the three countries.

References

1. Engle-Stone R, Ndjebayi AO, Nankap M, Killilea DW, Brown KH. Stunting prevalence, plasma zinc concentrations, and dietary zinc intakes in a nationally representative sample suggest a high risk

of zinc deficiency among women and young children in Cameroon. J Nutr 2014;144(3):382-91. doi: 10.3945/jn.113.188383.

2. Haile D, Brown KH, McDonald CM, Luo H, Jarvis M, Teta I, Ndjebayi A, Martial GAJ, Vosti SA, Engle-Stone R. Applying Zinc Nutrient Reference Values as Proposed by Different Authorities Results in Large Differences in the Estimated Prevalence of Inadequate Zinc Intake by Young Children and Women and in Cameroon. Nutrients 2022;14(4):883.
3. Engle-Stone R, Nankap M, Ndjebayi AO, Brown KH. Simulations based on representative 24-h recall data predict region-specific differences in adequacy of vitamin A intake among Cameroonian women and young children following large-scale fortification of vegetable oil and other potential food vehicles. J Nutr 2014;144(11):1826-34. doi: 10.3945/jn.114.195354.
4. Haile D, Luo H, Vosti SA, Dodd KW, Arnold CD, Engle-Stone R. Micronutrient Fortification of Commercially Available Biscuits Is Predicted to Have Minimal Impact on Prevalence of Inadequate Micronutrient Intakes: Modeling of National Dietary Data From Cameroon. Curr Dev Nutr 2020;4(9):nzaa132. doi: 10.1093/cdn/nzaa132.
5. Ministry of Health. The Kenya National Micronutrient Survey. Nairobi: Ministry of Health, 2011.
6. Sanchez-Pimienta TG, Lopez-Olmedo N, Rodriguez-Ramirez S, Garcia-Guerra A, Rivera JA, Carriquiry AL, Villalpando S. High Prevalence of Inadequate Calcium and Iron Intakes by Mexican Population Groups as Assessed by 24-Hour Recalls. J Nutr 2016;146(9):1874S-80S. doi: 10.3945/jn.115.227074.
7. Pedroza-Tobias A, Hernandez-Barrera L, Lopez-Olmedo N, Garcia-Guerra A, Rodriguez-Ramirez S, Ramirez-Silva I, Villalpando S, Carriquiry A, Rivera JA. Usual Vitamin Intakes by Mexican Populations. J Nutr 2016;146(9):1866S-73S. doi: 10.3945/jn.115.219162.
8. Armah SM, Carriquiry A, Sullivan D, Cook JD, Reddy MB. A complete diet-based algorithm for predicting nonheme iron absorption in adults. J Nutr 2013;143(7):1136-40. doi: 10.3945/jn.112.169904.
9. US Institute of Medicine. Dietary reference intakes for vitamin A, vitamin K, arsenic, boron, chromium, iodine, iron, manganese, molybdenum, nickel, silicon, vanadium, and zinc. Washington, D.C.: National Academy Press, 2001.
